# Mechanistic Features in Al(I)-Mediated Oxidative Addition of Aryl C–F Bonds: Insights From Density Functional Theory Calculations

**DOI:** 10.3389/fchem.2019.00596

**Published:** 2019-09-03

**Authors:** Xiangfei Zhang, Ping Li, Binju Wang, Zexing Cao

**Affiliations:** ^1^State Key Laboratory of Physical Chemistry of Solid Surfaces, Fujian Provincial Key Laboratory of Theoretical and Computational Chemistry, and College of Chemistry and Chemical Engineering, Xiamen University, Xiamen, China; ^2^School of Chemistry and Pharmaceutical Engineering, Huanghuai University, Zhumadian, China

**Keywords:** fluorobenzene, NacNacAl, density functional theory, C–F bond, reaction mechanism

## Abstract

The oxidative addition of a range of robust aryl C–F bonds to a single Al(I) center supported by a (NacNac)^−^ bidentate ligand ((NacNac)^−^ = [ArNC(Me)CHC(Me)NAr]^−^ and Ar = 2,6–${\text{Pr}}_{2}^{\text{i}}$C_6_H_3_) have been explored by density functional theory calculations. Our calculations demonstrate that the Al(I) center-mediated C–F insertion generally proceeds *via* the concerted mechanism that involve both the donation (${\text{n}}_{\text{Al}}\to \sigma_{\text{C}-\text{F}}^{*}$) and back-donation ($\sigma_{\text{F}\left(\text{p}\right)}\to \pi_{\text{Al}\left(\text{p}\right)}^{*}$) interactions. In addition, the predicted free energy barriers for the C–F bond activation show good agreement with the experimental information available. Finally, the comparative studies show that B(I) is the most active among group III metals (B, Al, Ga), thus supplying a testable prediction for experiments.

## Introduction

In natural organic halides, the fluorinated compounds have relatively low abundance (Harper et al., 2003), but their importance has been increasingly recognized in pharmaceuticals, advanced materials, agrochemicals, and polymer chemistry (Hiyama and Yamamoto, 2000; Müller et al., 2007; Purser et al., 2008; O'Hagan, 2010). This is mainly because the introduction of fluorine can significantly modify the electron-density distribution in a molecule or a building block, resulting in a dramatic change in their reactivity and properties, but inducing little effects in their steric hindrance.

The C–F bond is one of the strongest σ covalent single bonds, and its activation typically requires the transition metal catalysis (TMs) (Huang et al., 2018; Lin et al., 2019). In last decades, the functionalization of C–F bonds has received considerable interest, and extensive efforts have been made to develop various strategies for the C–F activation (Mazurek and Schwarz, 2003; Panetier et al., 2011; Johnson et al., 2012; Klahn and Rosenthal, 2012; Nova et al., 2012; Kuehnel et al., 2013). In the transition metal-mediated C–F activations, the strong repulsion interactions between the d-occupied orbital of TM and the electron-rich F atom would raise the activation energies for the concerted oxidative addition reactions. As such, the C–F activation would have a preference for the stepwise manner (Choi et al., 2011), which usually involves σ-bond metathesis or insertion–elimination reactions during these processes (Watson et al., 2001; Kraft et al., 2010; Nova et al., 2010).

**Scheme 1:**
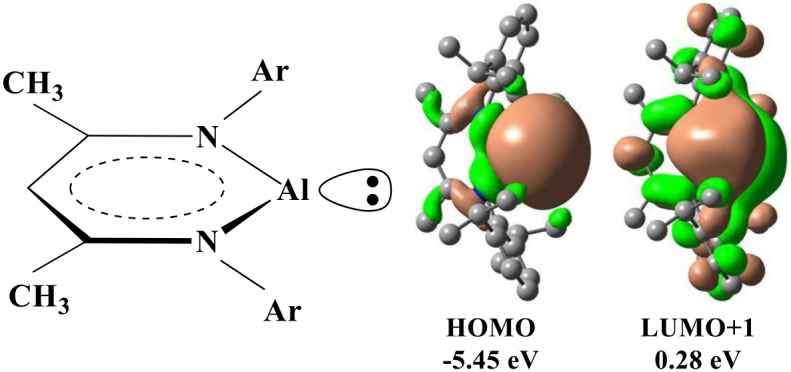
Schematic drawings for the structure of NacNacAl and its representative molecular orbitals.

In addition to the transition metals, the compounds of the some main-group elements may have the transition-metal-like reactivity and catalyze the oxidative addition of the C–F bond (Jana et al., 2010; Stahl et al., 2013; Swamy et al., 2017; Chu and Nikonov, 2018). In particular, recent experimental studies demonstrated that a monomeric Al(I) center supported by the NacNac^−^ ligand (NacNac^− =^ [ArNC(Me)CHC(Me)NAr]^−^ and Ar = 2,6–${\text{Pr}}_{2}^{\text{i}}$C_6_H_3_) could catalyze the oxidative addition of the C–F bond (Chu et al., 2015; Crimmin et al., 2015). As aluminum is non-toxic and the most abundant metal in nature, Al(I) compound represents a promising strategy for the C–F activation. In NacNacAl (1), the conjugate bidentate ligand NacNac^−^ can combine with Al^+^ through the N→ Al dative bond, in which HOMO is basically composed of an sp^2^-like lone pair occupied orbital at Al, while LUMO+1 is mainly contributed by the p-type orbital of Al (Scheme 1) (Cui et al., 2000; Schoeller and Frey, 2013).

Our previous studies show that the aluminum center of NacNacAl (1), as the N-heterocyclic carbene (NHC) analog, has similar electronic characters with the transition metal (Zhang and Cao, 2016). Although the C–F bond activation by the main-group element centers has been investigated, both experimentally (Meier and Braun, 2009; Caputo et al., 2013; Stahl et al., 2013; Chen et al., 2017; Bayne and Stephan, 2019; Pait et al., 2019) and theoretically (Mondal et al., 2017), the detailed mechanisms for the oxidative addition of C–F bonds at the Al(I) of NacNacAl (1) are still largely unknown. Herein, we have performed extensive density functional theory (DFT) calculations on the C–F bond activation, and the possible mechanisms for the diversity of oxidative addition reactions and dependence of the ease of C–F oxidative addition on the fluorination and position have been explored.

## Computational Details

**Scheme 2:**
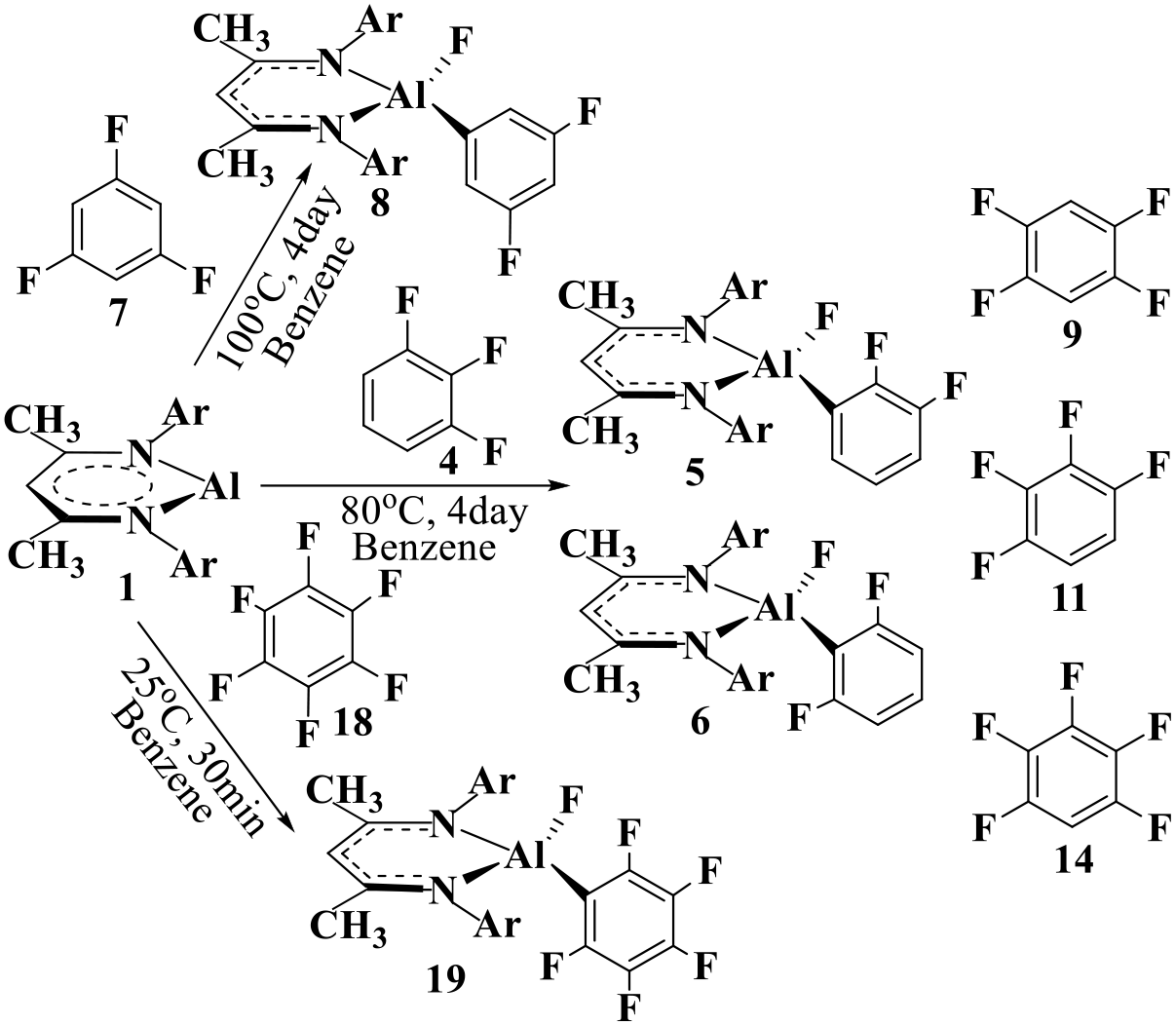
C–F addition reactions of NacNacAl (1) complex with the fluorinated benzenes. **4**, **7**, and **18** are from experiments, while the additional fluorobenzenes **9**, **11**, and **14** are also considered in this study.

For convenience, the aryl C(sp^2^)–F substrates are labeled as **4**, **7**, **9**, **11**, **14**, and **18** [Scheme 2 and Figure SI1], while the intermediates and transition states are labeled as **IM**_**1−Y**_ (**IM**_**1−Yiso**_) and **TS**_**1−X**_ (**TS**_**1−Yiso**_), respectively, where **Y** is the substrate label (Scheme 2 and **Figures 3**, **4**). The reactant complex is labeled as **R**_**1−Y**_, and the corresponding products are labeled with **Y+1**, **Y+2**, etc. for multiple reaction channels.

The geometries of reactants, intermediates, transition states, and products have been fully optimized by the DFT calculations with the functionals M06-2X (Zhao and Truhlar, 2008), and a comparison of structures by experiment and theory with different functionals is shown in Figure SI2. In particular, based on previous computational investigations and the evaluation of functional performance (Zhang and Cao, 2016), several selected functionals have been used for the aryl C–F bond systems (Figure SI3).

Frequency calculations at the same level of theory have been carried out to confirm if the optimized structures are local minima without any imaginary frequency, or transition states (**TS**) with only one imaginary frequency on the potential-energy surface (**PES**). The intrinsic reaction coordinate (**IRC**) (Fukui, 1970, 1981) analysis is used to track the minimum energy path correlating the transition state with the corresponding reactant and product. The natural bond orbital (**NBO**) analysis (Reed et al., 1988) has been also carried out to further examine the electronic and bonding properties of the optimized structures.

Here, two different basis sets are considered for all atoms. The relatively small basis set 6-31G(d) (Hehre et al., 1972; Francl et al., 1982) (**BS1**) is used for all of the geometry optimizations and frequency calculations, and the larger 6-311+G(d,p) basis set (**BS2**) is used for the single-point calculations with the SMD solvation model for estimation of the solvent effect of benzene (Marenich et al., 2009). The corrections of Gibbs free energy and zero-point energy (**ZPE**) from the gas-phase frequency calculations are used to determine the relative reaction energetics. In consideration of overestimation of the entropic effect from the gas-phase calculation, a correction of −2.6 (2.6) kcal/mol (Benson, 1982) (at *T* = 298.15 K) was applied to calibrate the relative free energies for the reaction step with the molecular number ratio of reactant to product of 2:1 (or 1:2) according to the free volume theory and previous theoretical calculations (Schoenebeck and Houk, 2010; Ariafard et al., 2011; Liu et al., 2012; Wang and Cao, 2013). Here, all calculations have been performed by the Gaussian 09 program (Frisch et al., 2009).

## Results and Discussion

Herein, NacNacAl (1) catalyzed oxidative addition of the aryl C–F bonds from various fluorobenzene derivatives has been investigated (see Scheme 2). In particular, substrates **4**, **7**, and **18** have been studied in experiments. For comparison, we also considered additional fluorobenzene derivatives **9**, **11**, and **14** in this study. It is seen from Scheme 2 that the reaction conditions for the C–F activation in substrate **7** is much harsher than that in substrate **18**, suggesting that substrate **18** is much more reactive than substrate **7**. Based on DFT calculations, the predicted relative energies are collected in Table SI1. The Cartesian coordinates of all molecules, intermediates, and transition states for their optimized structures are compiled into Table SI2.

### Addition of the Aryl σ C–F Bond

NacNacAl (1)-mediated oxidative additions of the aryl C–F bonds in **4**, **7**, **9**, **11**, **14**, and **18** have been explored.

**Figure 1:**
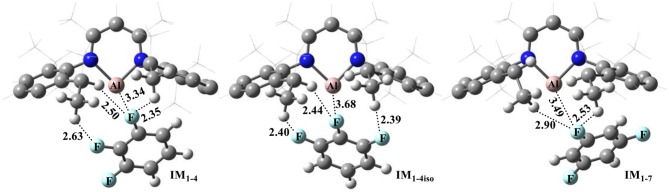
Optimized structures of reactant complexes of NacNacAl (1) with the trifluorobenzene derivatives (**4** and **7**). For clarity, only the key atoms are highlighted with the ball-and-stick model.

Figure 1 shows the optimized structures of complexes of NacNacAl (1) with substrates **4** and **7**; the optimized structures for other substrates are summarized in the Supporting Information (Table SI2). Note that F atoms from the substrates maintain a long distance with the Al center, but a relatively short distance with methyl H atoms. All these suggest that interactions between NacNacAl (1) and substrates are dominated by C–H···F-type H-bond interactions (Saha et al., 2018). For substrate **4**, two binding conformations (**IM**_**1−4**_ and **IM**_**1−4iso**_) are located in calculation, which would lead to two distinct products as observed in experiments (Scheme 2).

**Figure 2:**
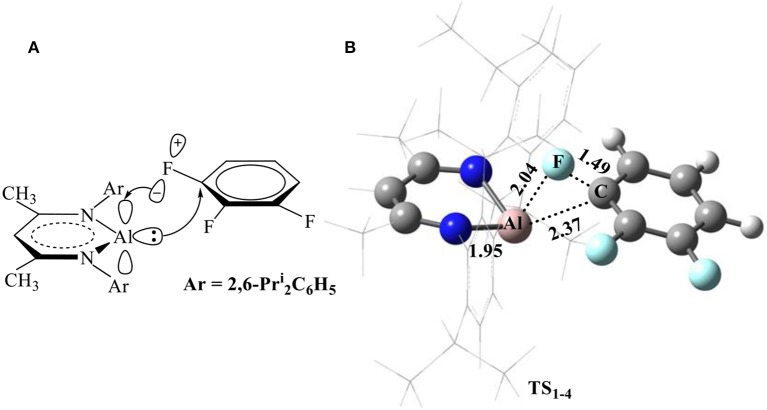
Proposed mechanism for the C–F bond activation **(A)** and the optimized transition-state structure for the C–F activation of substrate **4** (**B**, bond lengths in Å) in reactions **R**_**1−4**_.

Considering that the electronic structure of the Al(I) center in NacNacAl (1) resembles that of a transition metal (Cui et al., 2000; Schoeller and Frey, 2013), the NacNacAl (1)-mediated oxidative addition reactions may proceed *via* the concerted insertion mechanism. Indeed, the concerted insertion transition states have been located for all fluorobenzene substrates in this study (Figure SI4). Figure 2 presents the mechanism and the representative transition state structures for the C–F activation of substrate **4**.

It is seen from Figure 2B that NacNacAl (1)-mediated oxidative C–F addition involves a transition state of the three-membered ring structure **TS**_**1**__−**4**_, in which the Al–F and C–F distances are 2.04 and 1.49 Å, respectively. It was found that the C–F bond can be efficiently activated by the donation ($n_{Al}\to \sigma_{C-F}^{*}$) and back-donation ($\sigma_{F\left(p\right)}\to \pi_{Al\left(p\right)}^{*}$) interactions (Figure 2A) (Schoeller and Frey, 2013). In contrast to transition metals (Clot et al., 2011), the Al center in NacNacAl (1) has the additional empty p-orbital, which renders the donation ($n_{Al}\to \sigma_{C-F}^{*}$) and back-donation ($\sigma_{F\left(p\right)}\to \pi_{Al\left(p\right)}^{*}$) interactions simultaneously, thus resulting in the concerted mechanism for the C–F bond insertion reactions. The predicted free energy barrier for substrate **4** is 26.1 kcal/mol relative to **1** + **4**, and this reaction is remarkably exothermic, with the Gibbs free energies of the reaction Δ*G* of −91.9 kcal/mol (298.15 K). The C–F bond insertion reaction leads to the formation of the fluorinated species NacNacAlF(C_6_H_3_F_2_) (O'Hagan, 2010), in which the Al center has been converted to sp^3^ hybridization, from the initial sp^2^ hybridization.

**Figure 3:**
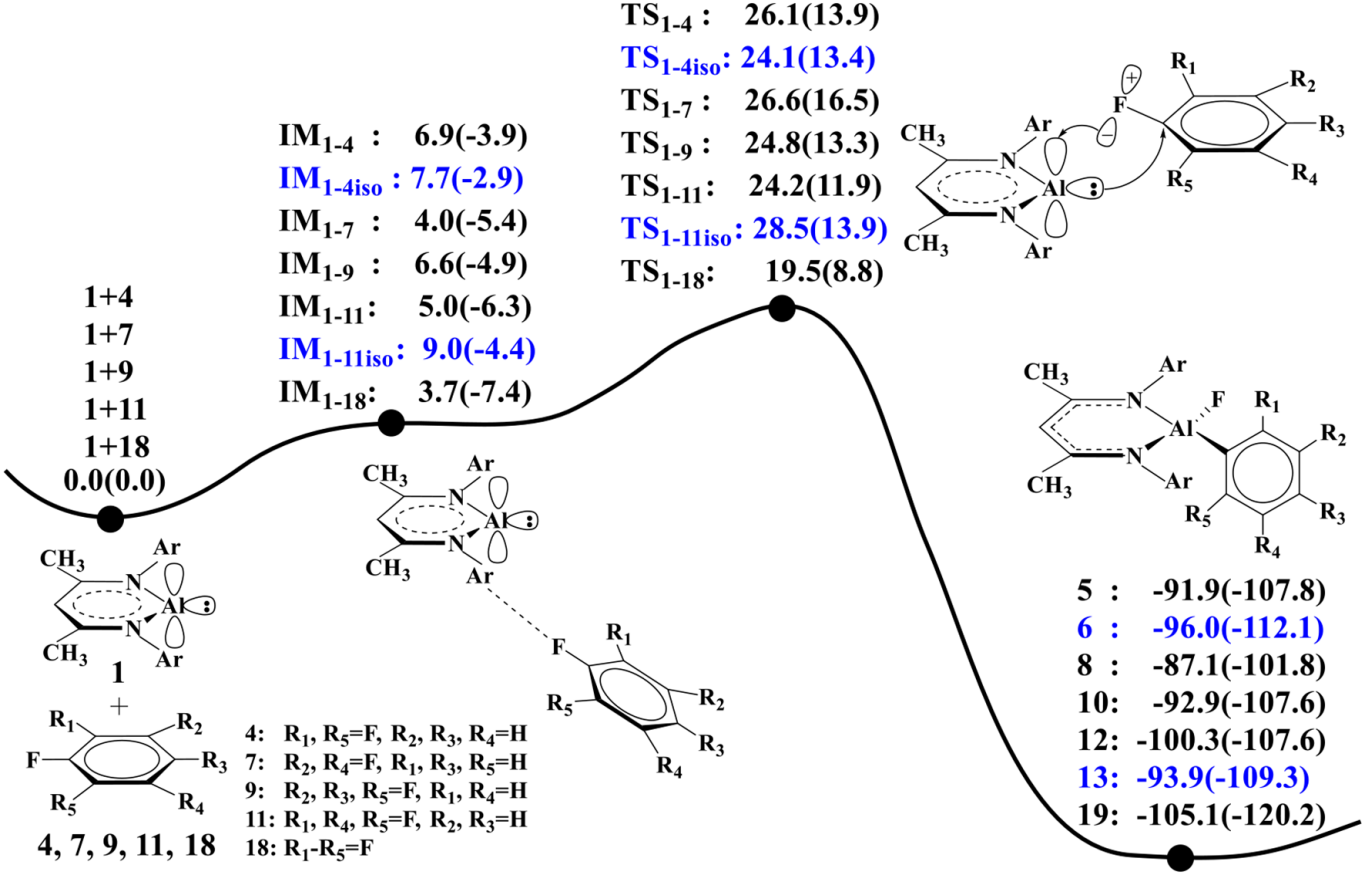
The predicted relative free energies and energies [Δ*G*(Δ*E*) in kcal/mol, the same below] here (the ZPE correction was included in relative energy in parentheses) for these oxidative additions are calculated by the M06-2X approach. The reactions of **1** with **4** and **11** produce two isomers **5**, **6**, and **12**, **13**.

Figure 3 compares the calculated relative energy profiles for all the fluorinated benzene substrates. We note that substrate **18** has the lowest overall free energy barrier (Δ*G*^≠^ = 19.5 kcal/mol) among these substrates, indicating that it is the most reactive. This is in accordance with the experimental findings (Scheme 2). For substrates **4** and **7**, the calculated lowest free energy barriers are 24.1 and 26.6 kcal/mol, respectively. The predicted reactivity order **18** > **4** > **7** is indeed in good agreement with experiments. In addition, our calculations predict that substrates **9** and **11** have similar reactivity with **4** and **7**, as all these substrates have similar free energy barriers for C–F activation. For comparison, we also considered the C–F activations of other isomers of **4** and **11** (**20** and **24** in Figure SI5), and the calculated lowest barriers are 26.8 kcal/mol for **20** and 24.5 kcal/mol for **24** (Figure SI6). It was found that the predicted reactivities for the C–F activation are correlated with the C–F bond strength of different substrates. Among all these substrates, **18** has the lowest bond dissociation energy (BDE = 122.4 kcal/mol, Figure SI5), while **7** has the highest bond dissociation energy (BDE = 126.5 kcal/mol). These BDE values show good correlations with the predicted reactivity of **18** and **7**.

**Figure 4:**
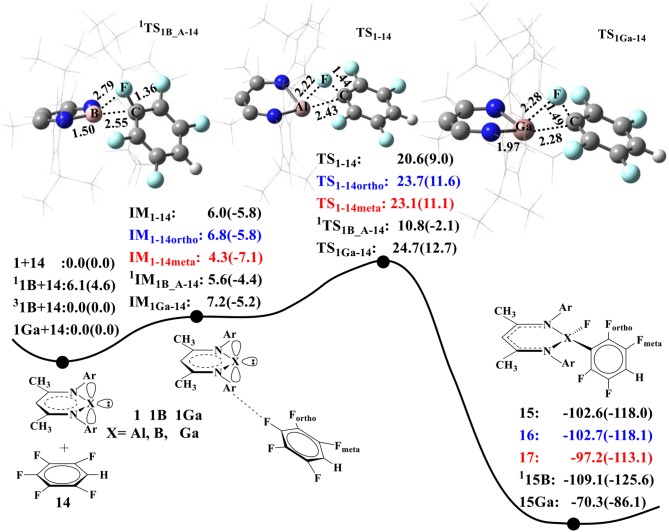
The predicted relative free energies and energies [Δ*G*(Δ*E*) in kcal/mol]. Optimized transition-state structures involved in the oxidative additions of 1,2,4,5-tetrafluorobenzene (Choi et al., 2011) to the NacNacAl (1), NacNacB (**1B**), and NacNacGa (**1Ga**) by M06-2X.

As B and Ga atoms in Group III have similar electronic configurations with Al, we also compared their performance in C–F activation. Figure 4 summarizes the calculated energy profiles for C–F activation of **14** by NacNacAl (1), NacNacB (**1B**), and NacNacGa (**1Ga**) (Asay et al., 2011). For NacNacAl (1), we have compared C–F activations at three different positions: para-, meta-, and ortho-positions, and the calculated barriers are 20.6 (**TS**_**1−14**_), 23.7 (**TS**_**1−14ortho**_), and 23.1 kcal/mol (**TS**_**1−14meta**_), respectively. All these indicate that the C–F bond at the para-position is the most active. As such, all the NacNacB (**1B**)- and NacNacGa (**1Ga**)-mediated C–F activations occur at the para-position. It is seen from Figure 4 that the calculated barriers for the para C–F activation are 20.6 kcal/mol for NacNacAl (1), 10.8 kcal/mol for triangle singlet transition state NacNacB (**1B**), and 24.7 kcal/mol for NacNacGa (**1Ga**), indicating that the electron-deficient compound **1B** has the highest reactivity toward C–F activation. Among these three elements, the B atom has the smallest atomic radius (Kutzelnigg, 1984), which results in stronger σ donor interactions than Al and Ga atoms. Meanwhile, we found that the ground state of NacNacB (**1B**) is actually the triplet state, instead of the singlet state as in NacNacAl (1) or NacNacB (**1Ga**). In the triplet state, we located both the linear and the triangle transition states that are characterized as “diradical” TS; the calculated barriers are 33 and 19.9 kcal/mol, respectively (Figures SI7, SI8). Clearly, the triangle transition state in the closed shell singlet state, with a barrier of 10.8 kcal/mol (Figure 4), is preferred over all other pathways (Table SI1). As such, we located a two-state reactivity for the NacNacB (**1B**)-mediated C–F activations.

## Conclusions

Extensive density functional calculations have been used to explore the oxidative additions of robust σ C(sp^2^)–F bonds to the Al(I) center, and plausible reaction mechanisms and optimized structures of reactants, intermediates, transition states, and products have been predicted. Our calculations demonstrate that all Al(I) center-mediated C–F insertions proceed *via* the concerted mechanism, which is governed by both the donation (${\text{n}}_{\text{Al}}\to \sigma_{\text{C}-\text{F}}^{*}$) and back-donation ($\sigma_{\text{F}\left(\text{p}\right)}\to \pi_{\text{Al}\left(\text{p}\right)}^{*}$) interactions. All the calculated C–F insertion mechanisms resemble that of the conventional transition-metal-like catalysis, and the predicted free energy barriers show good agreement with experiments.

## Data Availability

The datasets generated for this study are available on request to the corresponding author.

## Author Contributions

All authors listed have made a substantial, direct, and intellectual contribution to the work, and approved it for publication.

### Conflict of Interest Statement

The authors declare that the research was conducted in the absence of any commercial or financial relationships that could be construed as a potential conflict of interest.
