# Supplementary material for: Mechanistic Features in Al(I)-Mediated Oxidative Addition of Aryl C–F Bonds: Insights From Density Functional Theory Calculations
Source: Front Chem. 2019 Sep 3;7:596. doi: 10.3389/fchem.2019.00596 (PMC6733912; doi:10.3389/fchem.2019.00596)
Supplement: Supplementary file 1 [file Data_Sheet_1.PDF]

Supporting Information

Mechanistic Features in Al(I)−mediated Oxidative Addition of aryl C−F Bonds: Insights From DFT Calculations

Xiangfei Zhang[1,2], Ping Li[1] , Bingju Wang*[1], and Zexing Cao[*1]

[1] State Key Laboratory of Physical Chemistry of Solid Surfaces, Fujian Provincial Key Laboratory of Theoretical and Computational Chemistry, and College of Chemistry and Chemical Engineering, Xiamen University, Xiamen 360015, China

[2] School of Chemistry and Pharmaceutical Engineering, Huanghuai University, Zhumadian 463000, China

TABLE OF CONTENT

Figure SI1 ········S2
Figure SI2 ········S2
Figure SI3 ········S3
Figure SI4 ········S3
Figure SI5 ········S4
Figure SI6 ········S4
Figure SI7 ········S5
Figure SI8 ········S5
Table SI1 ········S6-S8
Table SI2 ········S9-S82

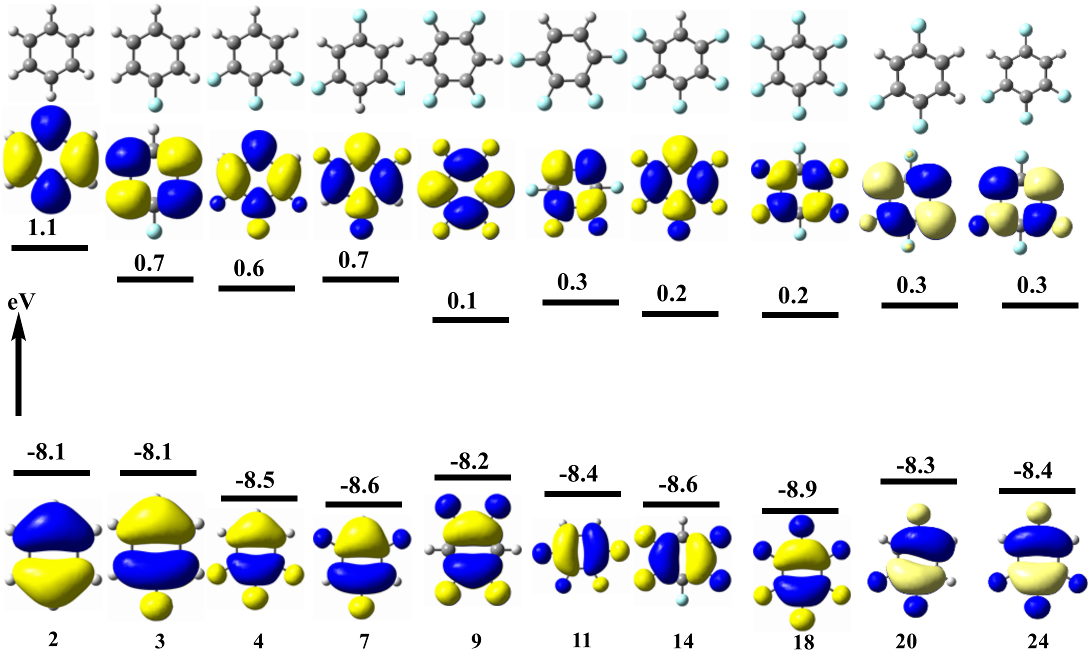

Figure SI1 | The selected HOMO and LUMO orbitals and their energy levels of reactants predicted by M06−2X/6−31G(d) (isovalue = 0.02).

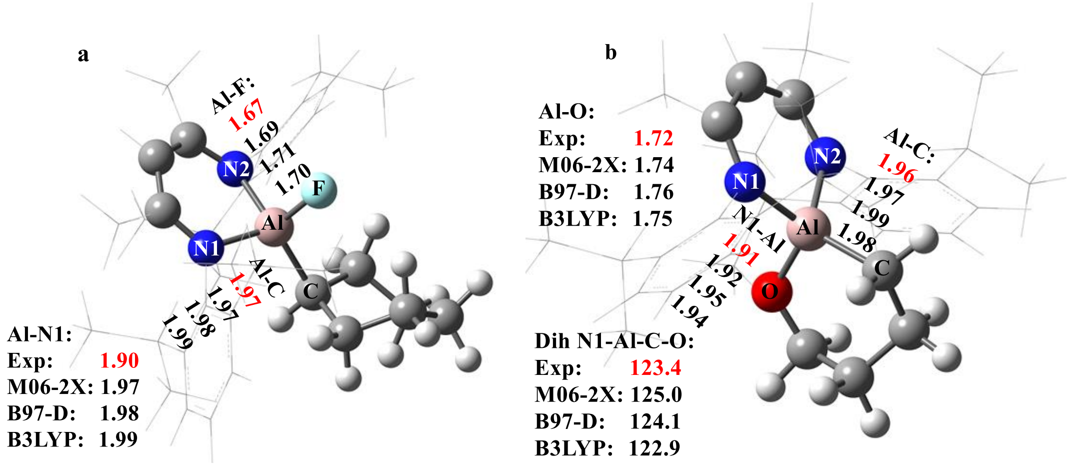

Figure SI2 | Predicted structure of the compound **NacNacAlF(C_6H_{11})** (a) and **NacNacAl(−$OCH_2CH_2CH_2CH_2$−)** (b) by different approaches (the experimental values are in read). Bond length in Å, Dihedral in angle.

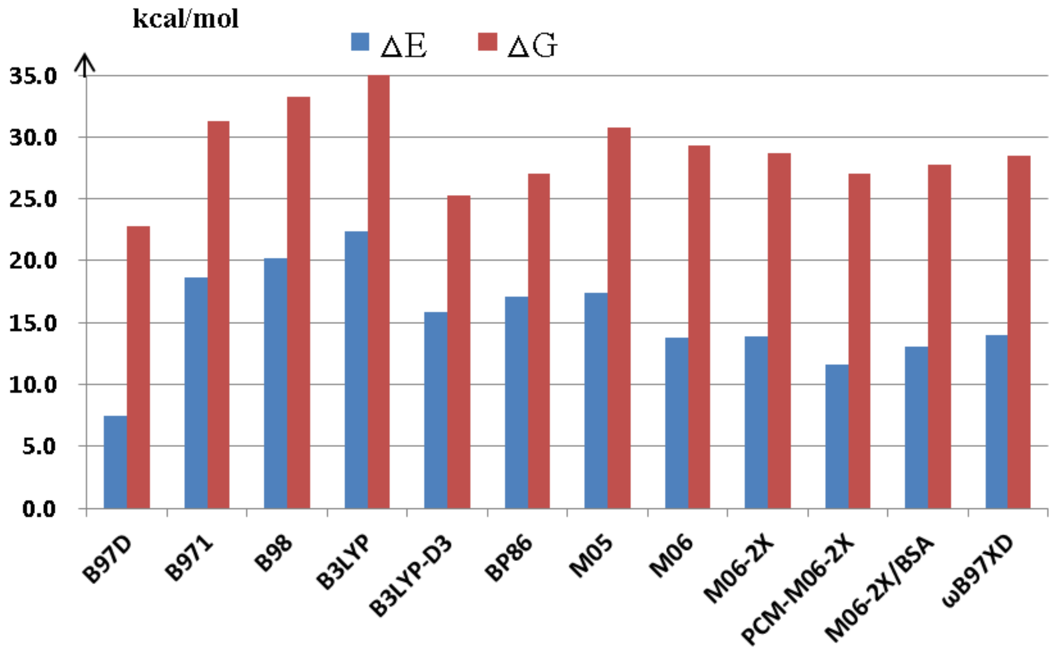

Figure SI3 | Related Gibbs free energies by selected functionals for the transition states involving in the reaction of $C_6H_3F_3$(**4**) to **NacNacAl**(**1**). (BSA: Aug−CC−PVQZ−DK for Al atom, 6−311G(2df) for F atom, and 6−31G(d) for other atoms.)

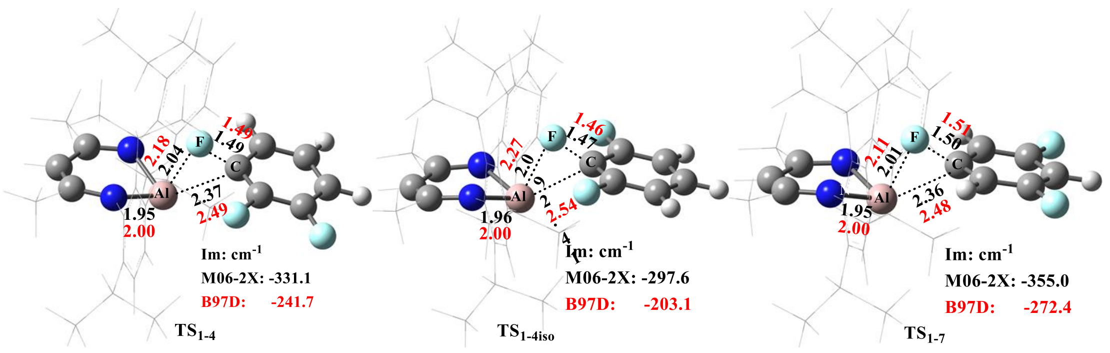

Figure SI4 | Optimized structures (Å) of the transition states involved in the oxidative additions of $C_6H_3F_3$(**4**,**7**) to NacNacAl(**1**) by M06−2X (in black) and B97−D (in red) methods.

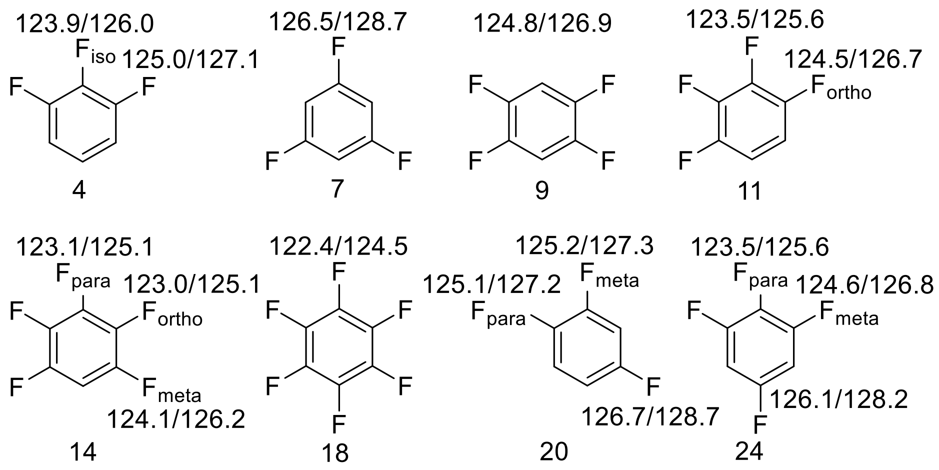

Figure SI5 | Selected bond dissociation energy (BDE, the unit of Δ*H*/Δ*E* is in kcal/mol, BDE=E(F−Aryl) − E(F·) − E(Aryl·)) of F−Aryl bonds predicted by M06−2X/6−311++G(2df,2p).

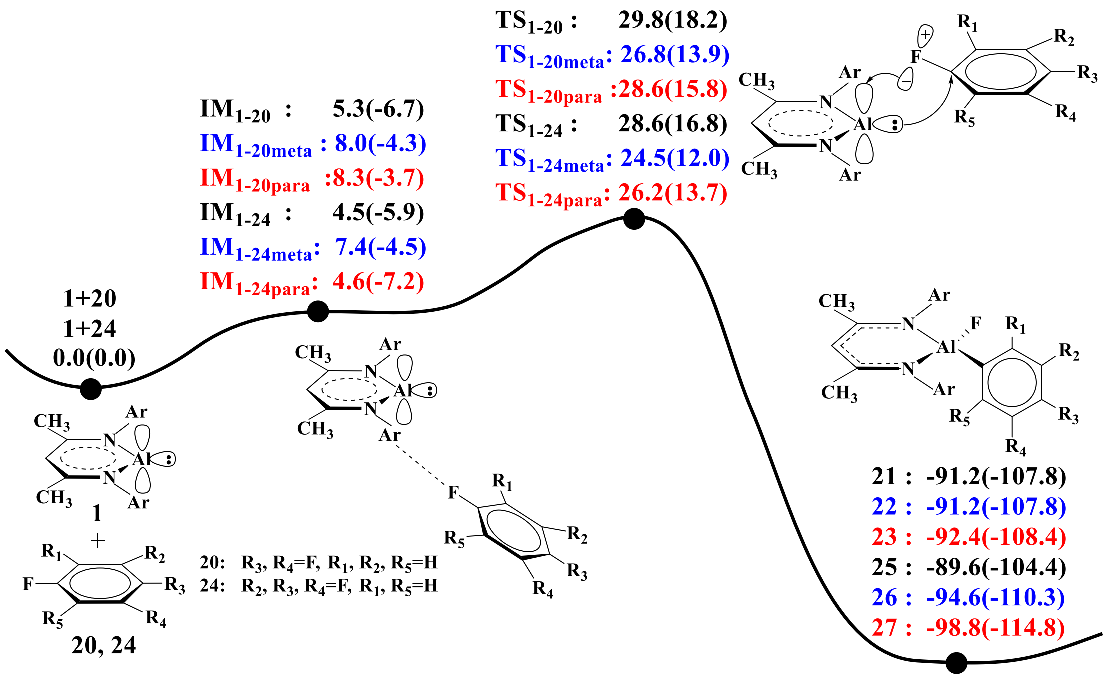

Figure SI6 | The predicted relative free energies and energies for these oxidative additions of **1** with **20** and **24** are calculated by the M06−2X approach.

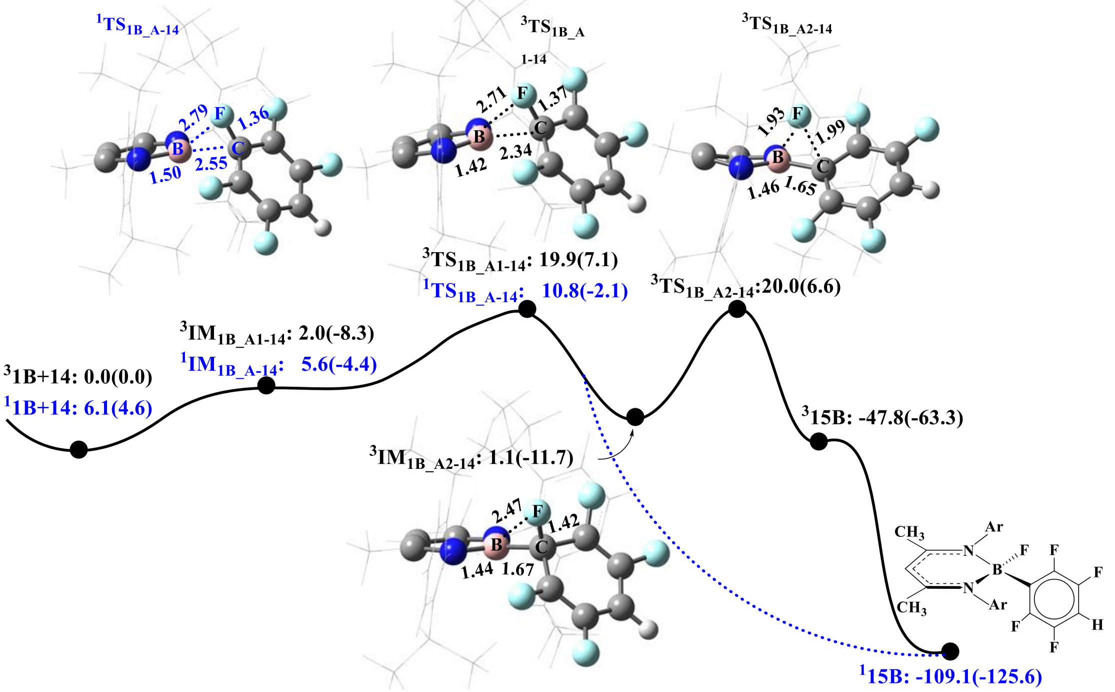

Figure SI7 | The predicted relative free energies and energies (ΔG(ΔE)) in kcal/mol. Optimized the shape of "triangle" transition-state structures involved in the oxidative additions of **14** to the NacNacB (**1B, ^{1}1B** is for single state, and **^{3}1B** is for Triple state) by M06−2X.

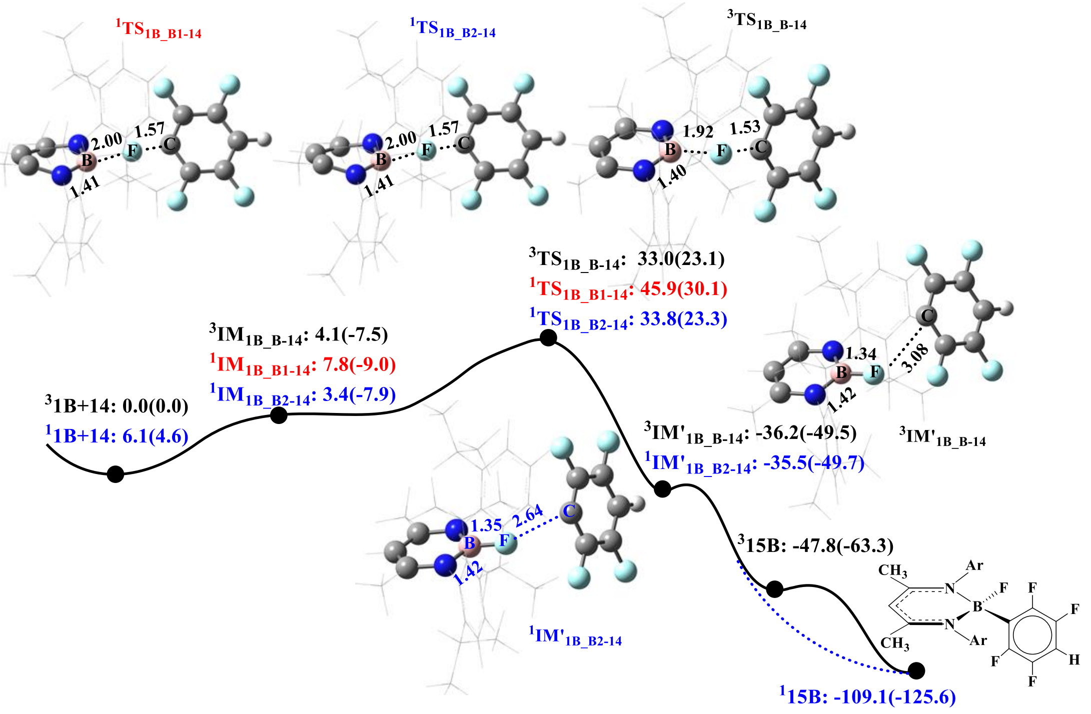

Figure SI8 | The predicted relative free energies and energies (ΔG(ΔE)) in kcal/mol. Optimized the shape of "linear" transition-state structures involved in the oxidative additions of **14** to the NacNacB (**1B, $^{1}TS_{1B\_B2-14}$** is calculated by open-shell singlet state using the function wave of triple state) by M06−2X.

Table SI1 | Predicted detailed thermodynamic data and selected bond lengths for the oxidative additions of F−Aryl bonds to the NacNacAl (**1**).

	ΔE [a]	ΔG [b]	dX−F [c] (X= B, Al, Ga)	dX−C [c]	dF−C [c]
**1**+**4**	0.0/0.0 [d]	0.0/0.0	–	–	1.33
**IM1-4**	-3.9/-2.8	6.9/9.3	3.34	3.43	1.34
**TS1-4**	13.9/7.5	26.1/20.7	2.04	2.37	1.49
**5**	-107.8/104.4	-91.9/87.4	1.67	1.97	3.12
**1**+**4**	0.0/0.0	0.0/0.0	–	–	1.33
**IM1-4iso**	-2.9/-2.8	7.7/9.1	3.68	3.59	1.34
**TS1-4iso**	13.4/7.1	24.1/18.9	2.09	2.41	1.47
**6**	-112.1/-107.8	-96.0/-91.6	1.67	1.97	3.05
**1**+**7**	0.0/0.0	0.0/0.0	–	–	1.34
**IM1-7**	-5.4/-4.1	4.0/6.6	3.49	3.65	1.34
**TS1-7**	16.5/10.2	26.6/21.6	2.01	2.36	1.50
**8**	-101.8/-99.4	-87.1/-84.2	1.68	1.96	3.01
**1**+**9**	0.0/0.0	0.0/0.0	–	–	1.33
**IM1-9**	-4.9/-4.9	6.6/6.3	4.04	3.62	1.34
**TS1-9**	13.3/6.6	24.8/18.6	2.04	2.37	1.49
**10**	-107.6/-104.9	-92.9/-89.7	1.67	1.97	3.11
**1**+**11**	0.0/0.0	0.0/0.0	–	–	1.33
**IM1-11**	-6.3/-4.1	5.0/8.4	4.59	3.99	1.33
**TS1-11**	11.9/5.3	24.2/17.1	2.14	2.42	1.45
**12**	-115.9/-112.6	-100.3/-96.7	1.67	1.97	3.05
**1**+**11**	0.0/0.0	0.0/0.0	–	–	1.33
**IM1-11orth**	-4.4/-4.6	9.0/10.4	3.62	3.58	1.34
**TS1-11orth**	13.9/6.7	28.5/21.7	2.04	2.38	1.49
**13**	-109.3/-104.8	-93.9/-87.7	1.68	1.98	3.03
**1**+**14**	0.0/0.0/0.0 [e]	0.0/0.0/0.0	–	–	1.33
**IM1-14**	-5.8/-5.4/0.4	6.0/7.3/8.3	4.56	4.05	1.33
**TS1-14**	9.0/3.1/16.2	20.6/14.8/26.0	2.22	2.43	1.44
**15**	-118.0/-115.6/-102.1	-102.6/-100.3/-88.2	1.67	1.98	3.05
**1**+**14**	0.0/0.0	0.0/0.0	–	–	1.33
**IM1-14ortho**	-5.8/-5.7	6.8/6.6	3.94	3.59	1.33
**TS1-14ortho**	11.6/4.5	23.7/16.5	2.13	2.42	1.46
**16**	-118.1/-115.1	-102.7/-99.3	1.67	1.97	3.05
**1**+**14**	0.0/0.0	0.0/0.0	–	–	1.33
**IM1-14meta**	-7.1/-5.4	4.3/6.8	3.88	3.76	1.33
**TS1-14meta**	11.1/4.4	23.1/16.8	2.09	2.38	1.47
**17**	-113.1/-110.8	-97.2/-94.6	1.68	1.96	2.94
^{1}**1B**+**14**	0.0	0.0	–	–	1.33
^{1}**IM1B_A-14**	-9.8 [f]	-0.5	3.17	3.16	1.33
^{1}**TS1B_A-14**	-6.7	4.7	2.79	2.55	1.36

^{1}**15B**	-130.2	-115.2	1.39	1.64	2.48
^{1}**1B+14**	0.0/0.0/0.0	0.0/0.0/0.0	–	–	1.33
^{1}**IM**$_{\mathbf{1B\_B1-14}}$	-9.0/-7.1/2.5	1.7/5.1/9.0	4.19	3.75	1.34
^{1}**TS**$_{\mathbf{1B\_B1-14}}$	30.1/9.3/22.4[e]	39.8/18.6/31.1	1.77	3.28	1.59
^{1}**15B**	-130.2/-119.9/-105.2	-115.2/-103.3/-89.1	1.39	1.64	2.48
^{1}**1B+14**	0.0	0.0	–	–	1.33
^{1}**IM**$_{\mathbf{1B\_B2-14}}$	-12.5	-2.7	3.60	3.46	1.33
^{1}**TS**$_{\mathbf{1B\_B2-14}}$	18.7	27.7	1.92	3.34	1.52
^{1}**IM’**$_{\mathbf{1B\_B2-14}}$	-54.1	-41.6	1.34	4.09	3.08
^{1}**15B**	-130.2	-115.2	1.39	1.64	2.48
^{3}**1B+14**	0.0	0.0	–	–	1.33
^{3}**IM**$_{\mathbf{1B\_A1-14}}$	-8.3	2.0	3.16	3.28	1.33
^{3}**TS**$_{\mathbf{1B\_A1-14}}$	7.1	19.9	2.71	2.34	1.37
^{3}**IM**$_{\mathbf{1B\_A2-14}}$	-11.7	1.1	2.47	1.70	1.42
^{3}**TS**$_{\mathbf{1B\_A2-14}}$	6.6	20.0	1.93	1.65	1.99
^{3}**15B**	-63.3	-47.8	1.40	1.62	2.50
^{1}**15B**	-125.6	-109.1	1.39	1.64	2.48
^{3}**1B+14**	0.0	0.0	–	–	1.33
^{3}**IM**$_{\mathbf{1B\_B-14}}$	-7.5	4.1	3.17	3.37	1.33
^{3}**TS**$_{\mathbf{1B\_B-14}}$	23.1	33.0	1.92	3.34	1.53
^{3}**IM’**$_{\mathbf{1B\_B-14}}$	-49.5	-36.2	1.34	4.09	3.08
^{3}**15B**	-63.3	-47.8	1.40	1.62	2.50
^{1}**15B**	-125.6	-109.1	1.39	1.64	2.48
**1Ga+14**	0.0/0.0	0.0/0.0	–	–	1.33
**IM**$_{\mathbf{1Ga-14}}$	-5.2/-5.8	7.2/6.2	4.60	4.09	1.33
**TS**$_{\mathbf{1Ga-14}}$	12.7/7.6	24.7/19.3	2.28	2.28	1.49
**15Ga**	-86.1/-83.5	-70.3/-67.5	1.72	1.99	3.11
**1+18**	0.0/0.0	0.0/0.0	–	–	1.33
**IM**$_{\mathbf{1-18}}$	-7.4/-6.8	3.7/4.8	3.84	3.66	1.33
**TS**$_{\mathbf{1-18}}$	8.8/2.0	19.5/12.2	2.22	2.43	1.45
**19**	-120.2/-117.6	-105.1/-102.8	1.67	1.98	3.05
**1+20**	0.0/0.0	0.0/0.0	–	–	1.34
**IM**$_{\mathbf{1-20}}$	-6.7/-3.8	5.3/8.2	4.60	3.96	1.34
**TS**$_{\mathbf{1-20}}$	18.2/11.6	29.8/23.8	2.04	2.25	1.48
**21**	-107.8/-99.4	-91.2/-83.4	1.68	1.97	3.07
**1+20**	0.0/0.0	0.0/0.0	–	–	1.34
**IM**$_{\mathbf{1-20mete}}$	-4.3/-3.5	8.0/9.0	3.40	3.46	1.34
**TS**$_{\mathbf{1-20meta}}$	13.9/7.2	26.8/20.7	2.04	2.38	1.48
**22**	-107.8/-104.9	-91.2/-87.9	1.68	1.97	3.07
**1+20**	0.0/0.0	0.0/0.0	–	–	1.34
**IM**$_{\mathbf{1-20para}}$	-3.7/-3.0	8.3/8.7	3.21	3.42	1.35
**TS**$_{\mathbf{1-20para}}$	15.8/8.0	28.6/20.4	2.01	2.37	1.51
**23**	-108.4/-104.6	-92.4/-87.4	1.68	1.97	3.08

**1+24**	0.0/0.0	0.0/0.0	–	–	1.34
**IM$_{1\text{-}24}$**	-5.9/-4.1	4.5/8.0	3.66	3.74	1.34
**TS$_{1\text{-}24}$**	16.8//9.8	28.6/22.0	2.00	2.37	1.50
**25**	-104.4/-101.7	-89.6/-85.8	1.68	1.97	3.06
**1+24**	0.0/0.0	0.0/0.0	–	–	1.33
**IM$_{1\text{-}24meta}$**	-4.5/-4.2	7.4/8.4	3.70	3.51	1.34
**TS$_{1\text{-}24meta}$**	-12.0/5.1	24.5/17.8	2.08	2.39	1.47
**26**	-110.3/-107.1	-94.6/-90.7	1.68	1.98	3.07
**1+24**	0.0/0.0	0.0/0.0	–	–	1.33
**IM$_{1\text{-}20para}$**	-7.2/-4.7	4.6/5.9	4.66	4.06	1.34
**TS$_{1\text{-}20para}$**	13.7/6.4	26.2/18.5	2.07	2.41	1.47
**27**	-114.8/-110.0	-98.8/-92.7	1.68	1.98	3.04

[a] The relative energies with the zero-point energy (ZPE) correction in kcal/mol; [b] The relative Gibbs free energies in kcal/mol; [c] Bond length in Å (optimized structures by M06−2X); [d] the relative energies by M06−2X/B97−D. [e] the relative energies by M06−2X/B97−D/B3LYP. [f] the relative energies by M06−2X.

Table SI2 | The M06−2X/6−31G(d) level predicted optimized geometries in terms of Cartesian coordinates for the reactants, products, intermediates and transition states located for all reactions presented.

1

Final structure in terms of initial Cartesian coordinates:

Al -0.000002 0.000230 -0.971042
N 1.388217 -0.000154 0.461550
N -1.388219 -0.000212 0.461553
C 1.246729 -0.000516 1.786553
C -1.246728 -0.000575 1.786555
C 0.000001 -0.000729 2.422057
H 0.000002 -0.001041 3.504085
C 2.721137 0.000099 -0.080043
C 3.345808 1.226186 -0.373310
C 3.346241 -1.225731 -0.373474
C 4.606201 1.201506 -0.972424
C 4.606643 -1.200519 -0.972552
C 5.235884 0.000625 -1.272695
H 5.098897 2.139949 -1.213657
H 5.099694 -2.138753 -1.213870
H 6.214603 0.000829 -1.742923
C -2.721140 -0.000037 -0.080038
C -3.346118 -1.225902 -0.373588
C -3.345934 1.226015 -0.373191
C -4.606518 -1.200762 -0.972672
C -4.606328 1.201264 -0.972301
C -5.235885 0.000347 -1.272694
H -5.099469 -2.139024 -1.214088
H -5.099123 2.139679 -1.213439
H -6.214604 0.000496 -1.742923
C 2.677504 2.561318 -0.084182
H 1.780836 2.371602 0.515959
C 2.678473 -2.561147 -0.084405
H 1.781583 -2.371790 0.515517
C -2.678200 -2.561275 -0.084672
H -1.781371 -2.371893 0.515333
C -2.677778 2.561190 -0.083914
H -1.781056 2.371501 0.516155
C -2.477387 -0.000865 2.661840
H -2.207307 -0.001183 3.718411
H -3.095930 0.877384 2.449755
H -3.095895 -0.879010 2.449215
C 2.477390 -0.000763 2.661835
H 2.207312 -0.001102 3.718406
H 3.095934 -0.878880 2.449200
H 3.095896 0.877513 2.449757
C -2.234597 -3.233032 -1.390644
H -3.102552 -3.452113 -2.023110
H -1.560002 -2.584177 -1.959214
H -1.717876 -4.176336 -1.182701
C -3.586420 -3.498487 0.719640
H -3.939493 -3.026965 1.642535
H -4.466130 -3.796596 0.139393
H -3.044217 -4.411104 0.987183
C -2.233875 3.233111 -1.389697
H -1.559331 2.584230 -1.958299
H -3.101703 3.452482 -2.022235
H -1.716997 4.176275 -1.181507
C -3.585908 3.498427 0.720471
H -4.465468 3.796855 0.140161
H -3.939221 3.026786 1.643213
H -3.043543 4.410868 0.988290
C 2.235041 -3.233158 -1.390306
H 1.560402 -2.584476 -1.959020
H 3.103066 -3.452225 -2.022679
H 1.718426 -4.176496 -1.182253
C 3.586759 -3.498123 0.720107
H 4.466544 -3.796210 0.139963
H 3.939712 -3.026417 1.642954
H 3.044653 -4.410768 0.987752
C 3.585562 3.498781 0.720021
H 4.465054 3.797235 0.139620
H 3.043099 4.411193 0.987741
H 3.938985 3.027315 1.642810
C 2.233445 3.232998 -1.390036
H 3.101210 3.452385 -2.022654
H 1.558950 2.583950 -1.958506
H 1.716462 4.176127 -1.181952

4

Final structure in terms of initial Cartesian coordinates:

C 0.000000 1.196085 0.014479
C 0.000000 0.000000 0.720030
C 0.000000 -1.196085 0.014479
C 0.000000 -1.210933 -1.371389
C 0.000000 0.000000 -2.058756
C 0.000000 1.210933 -1.371389
F 0.000000 0.000000 2.051459
F 0.000000 -2.335843 0.709040
F 0.000000 2.335843 0.709040
H 0.000000 -2.165884 -1.883801
H 0.000000 0.000000 -3.142981
H 0.000000 2.165884 -1.883801

IM$_{1-4}$

Final structure in terms of initial Cartesian coordinates:

Al 0.255981 0.029374 -0.807846
N 1.850125 -1.032440 -0.236575
N -0.858513 -1.568335 -0.349434
C 1.968603 -2.354804 -0.177116
C -0.462532 -2.841962 -0.413482
C 0.883664 -3.221422 -0.387860
H 1.097898 -4.281756 -0.417842
C 2.990809 -0.212937 0.062532
C 3.026093 0.454815 1.304197
C 3.996856 -0.020079 -0.900851
C 4.128466 1.261431 1.587969
C 5.077659 0.801129 -0.572266
C 5.154456 1.425648 0.664410
H 4.181084 1.783474 2.537813
H 5.864331 0.963973 -1.304005
H 6.003130 2.059451 0.902995
C -2.242093 -1.303831 -0.073512
C -3.161924 -1.104385 -1.117834
C -2.640065 -1.205705 1.279672
C -4.497240 -0.850341 -0.788458
C -3.989067 -0.979262 1.557213
C -4.916934 -0.808180 0.533414
H -5.219268 -0.693239 -1.585773
H -4.319700 -0.917901 2.589455
H -5.961335 -0.627088 0.769712
C 1.886713 0.300928 2.301827
H 0.952720 0.309471 1.726288
C 3.895586 -0.604778 -2.300956
H 3.132795 -1.390492 -2.294987
C -2.753998 -1.140184 -2.581210
H -1.711143 -1.472431 -2.634811
C -1.632309 -1.346953 2.414411
H -0.681387 -0.944337 2.048065
C -1.498135 -3.937854 -0.525320
H -2.252626 -3.863548 0.261975
H -2.024263 -3.837588 -1.481542
H -1.031059 -4.922903 -0.482898
C 3.307685 -2.979399 0.137785
H 3.727561 -2.540714 1.048372
H 3.210362 -4.057563 0.271142
H 4.024450 -2.786600 -0.666669
C -2.831165 0.264200 -3.193540
H -3.861449 0.639491 -3.163913
H -2.195222 0.967318 -2.646180
H -2.507910 0.243946 -4.239866
C -3.604893 -2.124750 -3.393072
H -3.598956 -3.127872 -2.954688
H -4.647752 -1.794625 -3.450669
H -3.225205 -2.195121 -4.417282
C -2.029282 -0.556525 3.665341
H -2.315686 0.469216 3.423545
H -2.858716 -1.037157 4.196501
H -1.182146 -0.522088 4.358873
C -1.393348 -2.813836 2.804928
H -2.345204 -3.311304 3.026167
H -0.879467 -3.375051 2.020724
H -0.770043 -2.863414 3.704621
C 3.428360 0.480384 -3.280981
H 2.470119 0.907356 -2.967379
H 4.161087 1.294086 -3.325714
H 3.313612 0.066442 -4.288591
C 5.209636 -1.228612 -2.783790
H 5.979498 -0.466078 -2.940690
H 5.605725 -1.957369 -2.068934
H 5.054585 -1.735560 -3.741389
C 1.960307 -1.037456 3.048392

H 2.930763 -1.143961 3.547033
H 1.178802 -1.087659 3.815950
H 1.819435 -1.890164 2.375923
C 1.777586 1.459328 3.293120
H 2.602484 1.457838 4.014472
H 1.761623 2.422379 2.776011
H 0.844657 1.363695 3.857804
C -1.613494 2.440425 0.765801
C -2.876449 2.250357 0.233460
C -0.757329 3.417747 0.284038
C -1.185827 4.224935 -0.759491
C -3.285977 3.073422 -0.813018
C -2.446572 4.065054 -1.314417
F -1.158070 1.646604 1.749928
H -3.501927 1.454301 0.623720
F -0.351069 5.159319 -1.220535
F 0.472776 3.543289 0.781000
H -4.268835 2.929967 -1.249122
H -2.742252 4.712560 -2.131724

TS$_{1-4}$

Final structure in terms of initial Cartesian coordinates:
Al 0.004898 0.001100 -0.801974
N 1.386635 -1.294946 -0.325816
N -1.387895 -1.322769 -0.461142
C 1.252676 -2.615520 -0.436126
C -1.238234 -2.638304 -0.600992
C 0.019938 -3.249916 -0.661852
H 0.039207 -4.323370 -0.795653
C 2.681595 -0.740079 -0.026946
C 2.994265 -0.426893 1.308620
C 3.579667 -0.455912 -1.073084
C 4.246527 0.125468 1.582673
C 4.810988 0.116113 -0.750233
C 5.152348 0.395552 0.566106
H 4.509137 0.361016 2.610472
H 5.512164 0.349605 -1.547275
H 6.117676 0.835544 0.797283
C -2.670601 -0.778594 -0.120187
C -3.509529 -0.257026 -1.120301
C -3.018724 -0.696647 1.245018
C -4.716395 0.331001 -0.732480
C -4.242335 -0.115364 1.580499
C -5.088232 0.397462 0.603428
H -5.373207 0.744526 -1.493393
H -4.533198 -0.053563 2.625714
H -6.032456 0.853724 0.885231
C 2.018894 -0.661599 2.451151
H 1.043919 -0.904318 2.019545
C 3.240566 -0.706210 -2.534133
H 2.301527 -1.269496 -2.579832
C -3.135588 -0.276018 -2.592602
H -2.202072 -0.839601 -2.700539
C -2.110220 -1.224753 2.346286
H -1.130113 -1.428887 1.903970
C -2.462546 -3.511348 -0.729661
H -2.198603 -4.568760 -0.687821
H -3.200372 -3.289816 0.044376
H -2.944782 -3.307625 -1.693535
C 2.478710 -3.490081 -0.338788
H 2.207245 -4.546092 -0.345677
H 3.145072 -3.288759 -1.184414
H 3.047407 -3.266402 0.567733
C -2.884335 1.151069 -3.096879
H -3.800390 1.749564 -3.030518
H -2.106874 1.647266 -2.506082
H -2.564598 1.135691 -4.144104
C -4.206074 -0.970075 -3.443165
H -4.421878 -1.979114 -3.077349
H -5.144949 -0.405864 -3.436364
H -3.873309 -1.044421 -4.483344
C -1.909465 -0.197982 3.466259
H -1.549291 0.754263 3.069080
H -2.838669 -0.016502 4.017068
H -1.173881 -0.575911 4.185016
C -2.647834 -2.538162 2.930291
H -3.648790 -2.392431 3.352279
H -2.711205 -3.327242 2.175351
H -1.991481 -2.892995 3.732005
C 3.023388 0.625640 -3.265569
H 2.242013 1.218529 -2.780058
H 3.946380 1.216563 -3.269082
H 2.729048 0.447660 -4.305518
C 4.318541 -1.531278 -3.248028
H 5.262712 -0.979923 -3.309477

H 4.521006 -2.476538 -2.734220
H 4.003390 -1.758274 -4.271513
C 2.450702 -1.846108 3.323531
H 3.437913 -1.666261 3.763961
H 1.737188 -1.997028 4.141201
H 2.503069 -2.774730 2.746050
C 1.844966 0.602255 3.300753
H 2.757238 0.836728 3.860076
H 1.600021 1.461807 2.672215
H 1.039441 0.462249 4.028656
C -0.490820 2.069870 0.251045
C -1.777938 2.629204 0.295388
C 0.626705 2.913793 0.251507
C 0.458729 4.269484 0.063358
C -1.914791 4.002930 0.134910
C -0.807945 4.842819 0.017980
F -0.298567 0.823986 1.043088
H -2.646283 1.983097 0.381728
F 1.551252 5.034277 -0.053026
F 1.851119 2.365125 0.234670
H -2.912197 4.430619 0.118327
H -0.898847 5.915927 -0.096440

5

Final structure in terms of initial Cartesian coordinates:
Al 0.052073 -0.526348 -0.733414
N -1.150897 -1.197154 0.582770
N 1.615988 -1.110113 0.160646
C -0.866078 -2.284303 1.286839
C 1.594501 -2.270115 0.833978
C 0.420159 -2.862513 1.302272
H 0.523440 -3.800134 1.832578
C -2.448150 -0.580249 0.647344
C -3.516430 -1.113823 -0.091068
C -2.566215 0.625761 1.364443
C -4.732481 -0.427499 -0.067604
C -3.801117 1.274045 1.353244
C -4.878586 0.752357 0.646526
H -5.571492 -0.816213 -0.638612
H -3.922653 2.207103 1.893386
H -5.829456 1.276437 0.643706
C 2.861662 -0.406686 0.033319
C 3.318310 0.361294 1.124676
C 3.568243 -0.443977 -1.181347
C 4.509156 1.073523 0.976987
C 4.744464 0.302397 -1.286246
C 5.219209 1.050586 -0.217891
H 4.878759 1.668006 1.808896
H 5.295357 0.293634 -2.222878
H 6.139175 1.619039 -0.315378
C -3.378401 -2.352248 -0.962375
H -2.413316 -2.824900 -0.752177
C -1.396052 1.186662 2.160340
H -0.471153 0.918570 1.636172
C 2.543264 0.475979 2.431394
H 1.619473 -0.106714 2.340558
C 3.067354 -1.234923 -2.377150
H 2.229681 -1.859610 -2.054676
C 2.902636 -2.966438 1.115842
H 3.425632 -3.152213 0.170966
H 3.564157 -2.342738 1.722015
H 2.737533 -3.914955 1.627317
C -1.942254 -2.949498 2.107807
H -2.600147 -3.529780 1.451424
H -1.505061 -3.625122 2.843826
H -2.564659 -2.208625 2.616159
C 2.150023 1.934122 2.701796
H 3.038397 2.563491 2.827459
H 1.555776 2.347374 1.881055
H 1.562580 2.004096 3.624057
C 3.337025 -0.075478 3.622666
H 3.606483 -1.126904 3.487605
H 4.262944 0.490819 3.772522
H 2.744148 0.004298 4.539927
C 2.540771 -0.281951 -3.458346
H 1.787547 0.404074 -3.057588
H 3.359006 0.322778 -3.867384
H 2.083041 -0.847104 -4.275598
C 4.144694 -2.162098 -2.950130
H 4.985068 -1.595839 -3.365780
H 4.541832 -2.838516 -2.186141
H 3.723860 -2.767818 -3.758692
C -1.422981 2.711812 2.264558
H -1.501717 3.171282 1.273408
H -2.258251 3.063538 2.879918

H -0.502427 3.068971 2.735103
C -1.334798 0.541368 3.550913
H -2.268004 0.717522 4.097959
H -1.177091 -0.540410 3.483207
H -0.510850 0.963974 4.137010
C -4.487302 -3.374766 -0.683337
H -5.461181 -3.000802 -1.016622
H -4.289989 -4.303018 -1.228767
H -4.572201 -3.610984 0.382774
C -3.372052 -1.963383 -2.447100
H -4.295736 -1.435459 -2.709948
H -2.522347 -1.319375 -2.679381
H -3.301479 -2.861546 -3.069936
C -0.078277 1.422977 -1.016966
C 0.916323 2.384535 -0.753156
C -1.282861 1.918572 -1.494889
C -1.527605 3.275211 -1.680782
C 0.692459 3.747967 -0.933426
C -0.541654 4.203321 -1.397277
F -0.157495 -1.448121 -2.119217
H 1.896352 2.062561 -0.409000
F -2.720688 3.667120 -2.143798
F -2.280243 1.067695 -1.809250
H 1.481418 4.462869 -0.721579
H -0.750934 5.256236 -1.553159

IM$_{1-4iso}$

Final structure in terms of initial Cartesian coordinates:
Al 0.162567 -0.341583 -0.699030
N 1.699761 -1.100688 0.315187
N -1.035035 -1.539614 0.352309
C 1.745258 -2.234056 1.014866
C -0.711251 -2.660640 0.993849
C 0.614497 -3.022600 1.265267
H 0.771100 -3.950392 1.799577
C 2.905783 -0.326835 0.189356
C 3.053532 0.826787 0.988470
C 3.879616 -0.689780 -0.757751
C 4.231950 1.563711 0.865335
C 5.038100 0.085174 -0.849023
C 5.223943 1.192444 -0.034892
H 4.373341 2.452349 1.471073
H 5.801077 -0.180258 -1.575993
H 6.132897 1.781314 -0.113857
C -2.417864 -1.153834 0.284995
C -3.243183 -1.638031 -0.743974
C -2.893876 -0.217570 1.229298
C -4.568814 -1.199647 -0.793667
C -4.226591 0.185149 1.140128
C -5.062752 -0.303902 0.142562
H -5.219174 -1.561731 -1.585797
H -4.616194 0.906501 1.850538
H -6.095421 0.027523 0.089509
C 1.949951 1.269663 1.941759
H 1.003247 1.181567 1.392657
C 3.671227 -1.845017 -1.724436
H 2.846229 -2.462061 -1.353254
C -2.729593 -2.576446 -1.823806
H -1.726961 -2.912601 -1.537037
C -1.986590 0.332786 2.324522
H -1.002418 0.515097 1.873784
C -1.800342 -3.596507 1.463444
H -2.571263 -3.058751 2.021595
H -2.292255 -4.052854 0.597518
H -1.389155 -4.389965 2.089167
C 3.058839 -2.705684 1.593732
H 3.523877 -1.908689 2.182146
H 2.911595 -3.581326 2.227208
H 3.762482 -2.961768 0.795105
C -2.609286 -1.833456 -3.161124
H -3.594673 -1.488169 -3.494663
H -1.956150 -0.959705 -3.069765
H -2.201970 -2.495233 -3.933167
C -3.616868 -3.816802 -1.987405
H -3.773651 -4.337232 -1.037294
H -4.602578 -3.548902 -2.382821
H -3.160754 -4.518254 -2.693310
C -2.479092 1.661887 2.901979
H -2.735618 2.374934 2.116522
H -3.356343 1.515118 3.542978
H -1.692026 2.102893 3.522484
C -1.791980 -0.672564 3.470252
H -2.763927 -1.009615 3.849647
H -1.213446 -1.547719 3.164229
H -1.254212 -0.197855 4.298135

C 3.256518 -1.300269 -3.098348
H 2.350384 -0.690218 -3.021804
H 4.052647 -0.671960 -3.514043
H 3.066710 -2.120472 -3.799158
C 4.905440 -2.743593 -1.859259
H 5.728500 -2.220953 -2.357428
H 5.271176 -3.087950 -0.886376
H 4.664368 -3.622538 -2.465562
C 1.853746 0.381284 3.189731
H 2.814546 0.360565 3.718013
H 1.101030 0.784847 3.876807
H 1.564438 -0.646172 2.952965
C 2.064676 2.733671 2.367632
H 2.919102 2.891997 3.035947
H 2.164686 3.394893 1.503995
H 1.161707 3.023231 2.913499
C -0.833412 3.100598 -0.459826
C -2.113884 3.137687 -0.990113
C 0.254161 3.230165 -1.309272
C 0.081981 3.416296 -2.669468
C -2.317618 3.316287 -2.348900
C -1.211715 3.456759 -3.183095
F -0.643529 2.896043 0.848542
F 1.478116 3.137048 -0.780790
H -3.333186 3.336064 -2.726369
H -1.360446 3.593116 -4.248470
F -3.150724 2.994522 -0.157694
H 0.958422 3.496820 -3.301425

$TS_{1\text{-}4iso}$

Final structure in terms of initial Cartesian coordinates:

Al -0.004882 -0.003641 -0.806995
N 1.384097 -1.263448 -0.247031
N-1.402282 -1.249711 -0.236681
C 1.232983 -2.587536 -0.218211
C-1.262377 -2.574964 -0.202368
C-0.018175 -3.215913 -0.275386
H-0.023703 -4.297625 -0.279741
C 2.692692 -0.715400 -0.006891
C 3.052486 -0.392570 1.315175
C 3.562434 -0.463920 -1.082631
C 4.322477 0.138276 1.544052
C 4.813999 0.089439 -0.805596
C 5.200969 0.378557 0.495657
H 4.622736 0.379056 2.560302
H 5.495446 0.297707 -1.626347
H 6.181988 0.800356 0.691963
C-2.708266 -0.690407 -0.006766
C-3.578011 -0.461582 -1.088029
C-3.066862 -0.336481 1.307303
C-4.828853 0.099208 -0.823136
C-4.336811 0.199511 1.524805
C-5.215550 0.416633 0.471722
H-5.510214 0.290061 -1.648161
H-4.636117 0.463882 2.535433
H-6.196390 0.842969 0.658917
C 2.105418 -0.597808 2.487481
H 1.114322 -0.824129 2.083378
C 3.173001 -0.732738 -2.527035
H 2.219808 -1.273439 -2.531330
C-3.189863 -0.764535 -2.526283
H-2.240962 -1.312650 -2.518607
C-2.116878 -0.509684 2.482224
H-1.125763 -0.740679 2.081433
C-2.491766 -3.445995 -0.103826
H-3.107118 -3.314894 -1.000742
H-2.218240 -4.497543 -0.013080
H-3.114854 -3.158494 0.746690
C 2.456029 -3.469841 -0.143217
H 3.094076 -3.195801 0.700468
H 2.174910 -4.519893 -0.058206
H 3.059283 -3.335503 -1.047984
C-2.971335 0.541063 -3.303049
H-3.902257 1.117484 -3.351476
H-2.209291 1.161789 -2.821575
H-2.647623 0.328790 -4.327550
C-4.230081 -1.638509 -3.237912
H-4.435738 -2.561273 -2.686051
H-5.179492 -1.106360 -3.358883
H-3.875076 -1.908742 -4.237454
C-1.997672 0.782297 3.298415
H-1.750690 1.629537 2.654536
H-2.931070 1.010769 3.824117
H-1.212514 0.678712 4.054293
C-2.541048 -1.675617 3.383683

H-3.550378 -1.513558 3.778673
H-2.538278 -2.627309 2.843371
H-1.856133 -1.769825 4.233323
C 2.965339 0.591266 -3.275041
H 2.211047 1.208897 -2.777795
H 3.901754 1.159494 -3.313515
H 2.636823 0.404468 -4.302943
C 4.206733 -1.599998 -3.255943
H 5.161796 -1.074683 -3.361482
H 4.401162 -2.537200 -2.724720
H 3.852303 -1.843519 -4.262550
C 2.537340 -1.782861 3.360286
H 3.547096 -1.625320 3.755997
H 1.855506 -1.899752 4.209512
H 2.537284 -2.721922 2.798539
C 1.982472 0.672090 3.337090
H 2.918132 0.894782 3.861193
H 1.722132 1.534030 2.718313
H 1.204904 0.541149 4.096819
C 0.025445 2.261441 0.012981
C-1.160824 3.002929 -0.057676
C 1.221361 2.983884 -0.087640
C 1.242809 4.320725 -0.429397
C-1.170496 4.339929 -0.398692
C 0.039529 5.012038 -0.577550
F 0.027355 1.131966 0.950662
F 2.367952 2.294136 0.027515
H-2.127706 4.837704 -0.507313
H 0.044777 6.066583 -0.826276
F-2.314581 2.330607 0.088981
H 2.204650 4.803389 -0.562585

6

Final structure in terms of initial Cartesian coordinates:
Al -0.039897 -0.654351 -0.639400
N -1.306446 -0.870988 0.774171
N 1.478747 -1.028017 0.434412
C -1.089578 -1.725207 1.766231
C 1.368900 -1.972350 1.384435
C 0.156981 -2.349167 1.965944
H 0.200229 -3.111135 2.733157
C -2.575776 -0.211689 0.637702
C -3.668999 -0.901281 0.088137
C -2.645408 1.154927 0.966969
C -4.857822 -0.193261 -0.098788
C -3.855488 1.817514 0.760184
C -4.955680 1.150324 0.234142
H -5.715427 -0.700768 -0.532179
H -3.937937 2.872081 1.002712
H -5.888351 1.683006 0.074479
C 2.786259 -0.469279 0.216638
C 3.239243 0.525982 1.102623
C 3.567305 -0.902847 -0.864945
C 4.516222 1.050118 0.904493
C 4.830399 -0.331768 -1.037258
C 5.309970 0.627440 -0.155342
H 4.890037 1.812636 1.583353
H 5.447382 -0.646468 -1.874289
H 6.298412 1.053589 -0.298296
C -3.584294 -2.349177 -0.371493
H -2.656017 -2.786271 0.010039
C -1.446539 1.881643 1.560291
H -0.538599 1.447215 1.125364
C 2.371193 1.070838 2.229604
H 1.376142 0.619311 2.145672
C 3.040866 -1.923269 -1.858915
H 2.179120 -2.427241 -1.410424
C 2.621422 -2.661552 1.870129
H 3.327218 -1.952837 2.309058
H 2.380975 -3.430371 2.604948
H 3.131887 -3.124423 1.017894
C -2.199884 -2.058592 2.731509
H -2.903453 -2.758727 2.267825
H -1.796643 -2.520253 3.633579
H -2.766146 -1.163958 3.002720
C 2.203793 2.590692 2.105448
H 3.164328 3.104688 2.221708
H 1.793116 2.860424 1.129371
H 1.533792 2.964841 2.887585
C 2.930790 0.715999 3.612679
H 2.992381 -0.365678 3.764901
H 3.935230 1.133674 3.745708
H 2.288171 1.128546 4.398074
C 2.548132 -1.210077 -3.125281
H 1.824290 -0.422273 -2.890489

H 3.389799 -0.740054 -3.647656
H 2.064694 -1.919355 -3.803681
C 4.073553 -2.997668 -2.211821
H 4.921874 -2.578708 -2.763701
H 4.466047 -3.487426 -1.314493
H 3.615034 -3.761553 -2.847502
C -1.428203 3.372887 1.223339
H -1.558179 3.532908 0.147445
H -2.216035 3.918988 1.754108
H -0.470484 3.808782 1.523707
C -1.376524 1.657019 3.076320
H -2.297656 2.002770 3.559281
H -1.239214 0.597419 3.317665
H -0.535510 2.209461 3.510180
C -4.757887 -3.185890 0.153809
H -5.697354 -2.897271 -0.329513
H -4.591594 -4.245197 -0.065547
H -4.894796 -3.072624 1.234584
C -3.516534 -2.429001 -1.902861
H -4.376534 -1.919756 -2.352403
H -2.600856 -1.970566 -2.279050
H -3.532273 -3.476610 -2.222256
C -0.236860 1.114180 -1.518636
C 0.683042 2.156344 -1.563313
C -1.437702 1.421066 -2.155298
C -1.745410 2.636904 -2.750007
C 0.460325 3.399508 -2.140464
C -0.775146 3.634315 -2.736104
F -0.244091 -1.929980 -1.709274
F -2.383284 0.459788 -2.206831
H 1.246096 4.146002 -2.120739
H -0.979475 4.595923 -3.195979
F 1.897089 1.956564 -1.005527
H -2.716577 2.775755 -3.210704

7

Final structure in terms of initial Cartesian coordinates:

C 0.000000 1.365434 0.000000
C 1.219088 0.703841 0.000000
C 1.182500 -0.682717 0.000000
C 0.000000 -1.407681 0.000000
C -1.182500 -0.682717 0.000000
C -1.219088 0.703841 0.000000
F 2.339841 -1.350908 0.000000
F 0.000000 2.701815 0.000000
H 0.000000 -2.490002 0.000000
H -2.156405 1.245001 0.000000
H 2.156405 1.245001 0.000000
F -2.339841 -1.350908 0.000000

IM_{1-7}

Final structure in terms of initial Cartesian coordinates:

Al -0.415049 0.399198 0.263350
N -2.206098 -0.366122 0.701647
N 0.386338 -1.320430 0.896811
C -2.504909 -1.415410 1.463508
C -0.167276 -2.245605 1.683406
C -1.528925 -2.258021 2.009233
H -1.872072 -3.054470 2.656251
C -3.276389 0.327071 0.039900
C -3.617637 -0.063829 -1.268420
C -3.911424 1.414474 0.666067
C -4.614506 0.650232 -1.935990
C -4.898942 2.101225 -0.042362
C -5.253404 1.725125 -1.331758
H -4.889177 0.362600 -2.947898
H -5.395710 2.948265 0.423671
H -6.024548 2.271558 -1.866148
C 1.758174 -1.515353 0.513267
C 2.799178 -1.108053 1.367235
C 2.027405 -2.099649 -0.742607
C 4.116392 -1.366993 0.979418
C 3.358047 -2.375018 -1.067608
C 4.396499 -2.025882 -0.209816
H 4.934097 -1.049796 1.620214
H 3.590518 -2.848303 -2.015985
H 5.424799 -2.240335 -0.484935
C -2.927939 -1.224063 -1.971002
H -2.241467 -1.698190 -1.259749
C -3.530745 1.872785 2.064072
H -2.875088 1.113147 2.503536
C 2.528689 -0.364388 2.665420

H 1.471194 -0.494821 2.919673
C 0.903813 -2.411775 -1.724008
H 0.174183 -1.593769 -1.646723
C 0.684012 -3.361016 2.246537
H 1.207687 -3.894059 1.447097
H 1.452233 -2.954358 2.912582
H 0.072381 -4.068619 2.807463
C -3.951757 -1.744274 1.742460
H -4.482896 -1.945868 0.806608
H -4.036433 -2.614247 2.394731
H -4.455183 -0.893319 2.212509
C 2.783699 1.136794 2.469850
H 3.828728 1.310328 2.189006
H 2.148668 1.546641 1.676467
H 2.579179 1.684835 3.396100
C 3.362104 -0.894902 3.837356
H 3.266614 -1.979876 3.951348
H 4.424891 -0.665408 3.707695
H 3.038604 -0.422176 4.769973
C 1.382514 -2.445583 -3.176714
H 1.942881 -1.542041 -3.430931
H 2.013332 -3.319414 -3.374719
H 0.520827 -2.513000 -3.847661
C 0.172017 -3.716904 -1.385248
H 0.873312 -4.559723 -1.384170
H -0.320684 -3.673652 -0.409980
H -0.599239 -3.919211 -2.137892
C -2.745067 3.188778 2.000857
H -1.841515 3.079244 1.392531
H -3.359157 3.979897 1.555590
H -2.451572 3.510143 3.005916
C -4.753367 2.023259 2.976869
H -5.398483 2.844269 2.646141
H -5.360603 1.112470 2.999226
H -4.435441 2.248587 3.999831
C -3.931173 -2.290834 -2.424425
H -4.615650 -1.896548 -3.183312
H -3.404157 -3.144440 -2.863885
H -4.534743 -2.655815 -1.587307
C -2.097598 -0.726056 -3.160452
H -2.736670 -0.231445 -3.900825
H -1.331435 -0.009126 -2.843639
H -1.599347 -1.567608 -3.655216
C 2.191414 1.672661 -1.946793
C 3.308241 1.181014 -1.291400
C 1.801347 3.001637 -1.891758
C 2.587358 3.852675 -1.130220
C 4.049270 2.088160 -0.551446
C 3.718654 3.430094 -0.445563
F 1.445593 0.813525 -2.657495
H 3.582634 0.134752 -1.341497
F 2.239060 5.141620 -1.044892
H 4.310896 4.115994 0.146265
H 0.907645 3.350552 -2.392174
F 5.129051 1.642454 0.108418

TS$_{1\text{-}7}$

Final structure in terms of initial Cartesian coordinates:

Al 0.035297 -0.150702 -0.829244
N 1.477322 -1.343758 -0.287797
N -1.295988 -1.478921 -0.328039
C 1.399529 -2.672533 -0.318199
C -1.096927 -2.793488 -0.363865
C 0.183459 -3.363549 -0.438706
H 0.237074 -4.442620 -0.498076
C 2.733338 -0.695113 -0.027365
C 3.049292 -0.335926 1.299097
C 3.581921 -0.350067 -1.097254
C 4.251697 0.334170 1.533845
C 4.763611 0.337181 -0.811610
C 5.105632 0.671398 0.491598
H 4.519288 0.603543 2.551805
H 5.424363 0.617044 -1.628134
H 6.031436 1.200822 0.694588
C -2.595612 -0.936765 -0.047181
C -3.446952 -0.562014 -1.103128
C -2.944986 -0.688362 1.296506
C -4.659480 0.056907 -0.789317
C -4.176568 -0.086204 1.558770
C -5.029303 0.290188 0.528202
H -5.322740 0.361268 -1.595008
H -4.466929 0.103465 2.588512
H -5.977052 0.770197 0.751779
C 2.140320 -0.663771 2.473992
H 1.172624 -0.981671 2.075131

C 3.243082 -0.663098 -2.545988
H 2.339439 -1.282854 -2.562593
C -3.086488 -0.781231 -2.562831
H -2.140004 -1.332239 -2.603773
C -2.034647 -1.059974 2.456910
H -1.055962 -1.326512 2.045462
C -2.288363 -3.719217 -0.359900
H -3.001638 -3.451969 0.422936
H -2.816877 -3.622892 -1.315848
H -1.981153 -4.757764 -0.232643
C 2.670212 -3.482904 -0.249686
H 2.454218 -4.550313 -0.196018
H 3.274122 -3.286754 -1.142841
H 3.278956 -3.192025 0.610220
C -2.878967 0.561051 -3.276603
H -3.809628 1.139661 -3.293272
H -2.111781 1.160242 -2.774676
H -2.563856 0.397303 -4.312569
C -4.148782 -1.616857 -3.287874
H -4.326414 -2.572889 -2.784850
H -5.105024 -1.084840 -3.335287
H -3.832969 -1.821852 -4.315805
C -1.833172 0.113117 3.422407
H -1.456939 0.996414 2.899509
H -2.767010 0.380961 3.928350
H -1.112265 -0.165079 4.198322
C -2.576203 -2.278355 3.216347
H -3.572158 -2.069362 3.623129
H -2.653960 -3.159516 2.572672
H -1.915925 -2.530800 4.052894
C 2.940465 0.630419 -3.314315
H 2.130084 1.191790 -2.837866
H 3.827826 1.272559 -3.349557
H 2.644153 0.403960 -4.343940
C 4.364314 -1.445240 -3.241604
H 5.275129 -0.842504 -3.323298
H 4.621808 -2.360600 -2.699261
H 4.059274 -1.721022 -4.255996
C 2.708799 -1.819655 3.307271
H 3.697544 -1.561987 3.703749
H 2.050510 -2.038207 4.155000
H 2.810134 -2.735323 2.716586
C 1.899715 0.561235 3.363299
H 2.819061 0.877321 3.867921
H 1.518538 1.406067 2.782801
H 1.168196 0.321618 4.141189
C -0.168116 2.007883 0.105675
C -1.445264 2.596791 0.101794
C 0.997021 2.796083 0.144058
C 0.844013 4.157938 -0.024637
C -1.507166 3.966845 -0.066309
C -0.389389 4.789439 -0.143836
F -0.082357 0.769489 0.950877
H -2.355787 2.008476 0.146828
F 1.947837 4.918533 -0.067151
H -0.473817 5.859054 -0.278124
H 1.985781 2.355439 0.214793
F -2.717491 4.539125 -0.144957

8

Final structure in terms of initial Cartesian coordinates:

Al -0.046954 -0.607760 -0.734703
N -1.368613 -1.212354 0.490324
N 1.425088 -1.305186 0.236232
C -1.198867 -2.331799 1.186003
C 1.290174 -2.437240 0.934820
C 0.049340 -2.969305 1.306899
H 0.064966 -3.905161 1.849664
C -2.629842 -0.523401 0.503059
C -3.659889 -0.923673 -0.363783
C -2.748466 0.616071 1.323547
C -4.844885 -0.180618 -0.359508
C -3.953135 1.317618 1.297514
C -4.997368 0.921684 0.468107
H -5.653544 -0.467791 -1.026361
H -4.073434 2.201243 1.915122
H -5.923398 1.488190 0.455618
C 2.718975 -0.686439 0.133814
C 3.192664 0.088582 1.211014
C 3.448351 -0.798337 -1.063443
C 4.413473 0.748395 1.062547
C 4.659543 -0.110114 -1.166939
C 5.143485 0.656970 -0.116343
H 4.790086 1.357971 1.879948
H 5.227616 -0.175929 -2.090989

H 6.085984 1.186498 -0.215992
C -3.504114 -2.076460 -1.342716
H -2.578345 -2.611875 -1.108199
C -1.588169 1.078589 2.193078
H -0.662449 0.857510 1.647612
C 2.400158 0.280735 2.495690
H 1.511188 -0.360022 2.461418
C 2.944711 -1.608241 -2.245594
H 2.052644 -2.159320 -1.936744
C 2.531241 -3.162987 1.388247
H 3.242992 -3.253953 0.562275
H 3.034277 -2.602922 2.182829
H 2.282219 -4.154784 1.766916
C -2.381209 -2.956067 1.883890
H -3.055522 -3.397921 1.141817
H -2.058834 -3.737590 2.572621
H -2.955678 -2.203371 2.430706
C 1.921358 1.733815 2.609914
H 2.774049 2.420452 2.658295
H 1.308744 2.021377 1.749647
H 1.329856 1.868544 3.522697
C 3.212915 -0.098083 3.740416
H 3.605302 -1.117993 3.683537
H 4.064439 0.577531 3.874318
H 2.587013 -0.023015 4.635670
C 2.537111 -0.676368 -3.394371
H 1.821492 0.083465 -3.063867
H 3.414732 -0.156328 -3.796407
H 2.071585 -1.248667 -4.202148
C 3.982400 -2.631649 -2.720608
H 4.880355 -2.141731 -3.112201
H 4.291486 -3.297191 -1.907987
H 3.562891 -3.244517 -3.524529
C -1.607926 2.586031 2.449468
H -1.680452 3.142185 1.508828
H -2.443892 2.878692 3.094060
H -0.687620 2.889599 2.956074
C -1.536463 0.302136 3.515575
H -2.475683 0.420348 4.068061
H -1.365960 -0.766775 3.350778
H -0.721308 0.675039 4.146484
C -4.670200 -3.068750 -1.258094
H -5.603047 -2.616524 -1.611213
H -4.468514 -3.938747 -1.890770
H -4.840270 -3.418983 -0.234682
C -3.368081 -1.537403 -2.773183
H -4.265805 -0.977357 -3.059063
H -2.499146 -0.881492 -2.867856
H -3.239701 -2.364147 -3.479200
C -0.083420 1.341475 -1.028497
C 1.058078 2.159289 -0.951000
C -1.307131 1.963926 -1.339023
C -1.364858 3.335030 -1.542431
C 0.953559 3.528258 -1.156384
C -0.248119 4.151886 -1.451224
F -0.248979 -1.516067 -2.133267
H 2.045994 1.759780 -0.733032
F -2.544530 3.904680 -1.835698
H -0.310112 5.221401 -1.610906
H -2.237883 1.407532 -1.425034
F 2.057220 4.287949 -1.068295

9

Final structure in terms of initial Cartesian coordinates:
C 0.000000 0.000000 1.400518
C 0.000000 1.192607 0.694739
C 0.000000 1.192607 -0.694739
C 0.000000 0.000000 -1.400518
C 0.000000 -1.192607 -0.694739
C 0.000000 -1.192607 0.694739
F 0.000000 2.358597 1.342680
F 0.000000 2.358597 -1.342680
F 0.000000 -2.358597 1.342680
F 0.000000 -2.358597 -1.342680
H 0.000000 0.000000 2.483662
H 0.000000 0.000000 -2.483662

IM_{1-9}

Final structure in terms of initial Cartesian coordinates:
Al 0.291982 0.018283 -0.761382
N 1.970025 -0.958276 -0.291464
N -0.700943 -1.647676 -0.294037

C 2.165133 -2.273305 -0.238814
C -0.242945 -2.898740 -0.355807
C 1.122606 -3.201902 -0.385758
H 1.395901 -4.248738 -0.417211
C 3.083415 -0.077062 -0.073119
C 3.172745 0.602412 1.160659
C 4.005705 0.161772 -1.107673
C 4.245613 1.472561 1.355900
C 5.059583 1.045274 -0.864814
C 5.189983 1.686536 0.358314
H 4.339497 2.005363 2.295991
H 5.781745 1.242096 -1.652595
H 6.015258 2.370836 0.530310
C -2.070345 -1.431743 0.073838
C -3.059480 -1.235188 -0.904756
C -2.372955 -1.346598 1.451415
C -4.366259 -0.974615 -0.481764
C -3.700258 -1.127783 1.823096
C -4.693196 -0.938299 0.865814
H -5.139480 -0.803221 -1.226016
H -3.960552 -1.078770 2.876350
H -5.717583 -0.750759 1.173402
C 2.128723 0.392964 2.250965
H 1.144265 0.386439 1.761592
C 3.840562 -0.442730 -2.493656
H 3.118573 -1.263404 -2.431868
C -2.761392 -1.313039 -2.393189
H -1.713035 -1.609969 -2.512260
C -1.284276 -1.472703 2.510855
H -0.353550 -1.105544 2.062262
C -1.222147 -4.049009 -0.414810
H -1.989981 -3.970307 0.358346
H -1.737209 -4.026291 -1.382481
H -0.709527 -5.007074 -0.316088
C 3.553955 -2.820235 -0.004004
H 4.015789 -2.334554 0.860892
H 3.522687 -3.897640 0.162481
H 4.199101 -2.615761 -0.864471
C -2.945204 0.046867 -3.078429
H -3.971282 0.410736 -2.961832
H -2.268851 0.797116 -2.657306
H -2.733342 -0.040219 -4.149500
C -3.634061 -2.372010 -3.080389
H -3.551100 -3.348286 -2.592294
H -4.690219 -2.081953 -3.063140
H -3.339148 -2.483982 -4.128702
C -1.570769 -0.626818 3.755302
H -1.828122 0.402849 3.496939
H -2.384410 -1.052653 4.352960
H -0.682407 -0.602634 4.395482
C -1.041632 -2.929338 2.933463
H -1.979167 -3.394847 3.259771
H -0.617237 -3.530427 2.125422
H -0.336831 -2.962943 3.771743
C 3.259407 0.608616 -3.449638
H 2.301355 0.989318 -3.080340
H 3.946563 1.457219 -3.543836
H 3.104587 0.180625 -4.445907
C 5.149261 -1.008731 -3.056547
H 5.869459 -0.212421 -3.271702
H 5.626077 -1.707941 -2.361627
H 4.956841 -1.535937 -3.996269
C 2.309838 -0.956432 2.959278
H 3.320932 -1.034989 3.375559
H 1.596882 -1.049123 3.786926
H 2.145018 -1.802371 2.284497
C 2.080362 1.525376 3.276931
H 2.980221 1.544941 3.901748
H 1.963955 2.497417 2.790057
H 1.222313 1.376648 3.939468
C -1.301379 2.595713 1.221084
C -2.519965 2.111406 0.774450
C -0.526098 3.427300 0.425781
C -0.952726 3.791154 -0.839731
C -2.947004 2.478547 -0.490933
C -2.166420 3.301590 -1.293819
F -0.829043 2.239305 2.424481
H -3.118366 1.439210 1.380387
F 0.658940 3.839683 0.884436
F -2.603480 3.606536 -2.518071
F -4.117591 2.040096 -0.963482
H -0.334671 4.414495 -1.473895

TS_{1-9}

Final structure in terms of initial Cartesian coordinates:

Al	0.205881	-0.093465	-0.800772
N	1.875638	-0.979819	-0.303749
N	-0.794755	-1.728902	-0.453877
C	2.087898	-2.292925	-0.383499
C	-0.308652	-2.962969	-0.575380
C	1.065431	-3.228600	-0.610643
H	1.364428	-4.262217	-0.723578
C	2.985678	-0.109405	-0.013220
C	3.192963	0.312705	1.312598
C	3.807239	0.346156	-1.061786
C	4.274391	1.155692	1.577099
C	4.865011	1.201171	-0.749028
C	5.109414	1.595744	0.559597
H	4.457768	1.478733	2.598422
H	5.505984	1.566781	-1.546842
H	5.942587	2.254725	0.784310
C	-2.175452	-1.537714	-0.113883
C	-3.125032	-1.260162	-1.112327
C	-2.526718	-1.548787	1.252894
C	-4.442656	-1.009009	-0.719292
C	-3.859445	-1.312454	1.592870
C	-4.813870	-1.041503	0.618256
H	-5.188090	-0.782882	-1.477319
H	-4.153284	-1.326120	2.638888
H	-5.843254	-0.846178	0.902652
C	2.284988	-0.112560	2.455530
H	1.408773	-0.605109	2.025641
C	3.554726	-0.018221	-2.516548
H	2.794316	-0.806719	-2.550329
C	-2.763937	-1.193548	-2.585889
H	-1.717007	-1.499562	-2.694241
C	-1.503167	-1.812034	2.348585
H	-0.506564	-1.731580	1.903408
C	-1.263492	-4.124254	-0.711582
H	-2.051864	-4.092778	0.043315
H	-1.757758	-4.061900	-1.688744
H	-0.737102	-5.077229	-0.646624
C	3.494542	-2.822623	-0.244530
H	3.964794	-2.446612	0.668158
H	3.500798	-3.912798	-0.231611
H	4.109669	-2.474287	-1.080987
C	-2.889042	0.245005	-3.104638
H	-3.925152	0.594813	-3.029729
H	-2.254394	0.927403	-2.529103
H	-2.587402	0.299213	-4.155948
C	-3.622789	-2.148393	-3.423762
H	-3.564400	-3.176045	-3.050913
H	-4.676418	-1.849378	-3.410231
H	-3.290527	-2.140165	-4.466681
C	-1.590218	-0.778170	3.476549
H	-1.522577	0.239803	3.084090
H	-2.525472	-0.871963	4.038958
H	-0.768492	-0.933058	4.184779
C	-1.649455	-3.228723	2.919887
H	-2.651493	-3.374840	3.338993
H	-1.486172	-3.995763	2.157227
H	-0.920204	-3.391727	3.720446
C	3.002671	1.196750	-3.274600
H	2.085165	1.568490	-2.807661
H	3.737555	2.009928	-3.277248
H	2.780798	0.933475	-4.314335
C	4.812351	-0.549321	-3.215038
H	5.574066	0.231635	-3.309903
H	5.263067	-1.386587	-2.672494
H	4.565039	-0.889509	-4.225582
C	2.979266	-1.120984	3.377992
H	3.887885	-0.690485	3.813951
H	2.313773	-1.408942	4.199487
H	3.262603	-2.030687	2.838752
C	1.789303	1.100133	3.251377
H	2.605262	1.571067	3.810358
H	1.353156	1.849675	2.586337
H	1.027716	0.791402	3.974624
C	-0.869576	1.770192	0.200425
C	-2.262636	1.922174	0.280403
C	-0.069931	2.923167	0.161180
C	-0.613013	4.174209	-0.024107
C	-2.802949	3.185945	0.111278
C	-1.998066	4.307182	-0.039408
F	-0.308090	0.664347	1.027564
H	-2.925999	1.070752	0.393333
F	1.263076	2.752071	0.149460
F	-2.562567	5.512610	-0.189712
F	-4.131924	3.345364	0.117022
H	0.026690	5.040592	-0.145752

10

Final structure in terms of initial Cartesian coordinates:

Al 0.114637 -0.090931 -0.448897
N -1.002381 -1.159107 0.673030
N 1.605037 -0.024756 0.729112
C -0.783367 -1.310163 1.976084
C 1.496341 -0.263861 2.038244
C 0.342432 -0.784612 2.636850
H 0.378160 -0.927232 3.708769
C -2.134011 -1.802936 0.049464
C -1.907120 -2.990829 -0.674067
C -3.422318 -1.239692 0.144690
C -3.002517 -3.622177 -1.266061
C -4.479686 -1.903241 -0.482158
C -4.280326 -3.087982 -1.175191
H -2.850731 -4.551483 -1.807122
H -5.478548 -1.479570 -0.420090
H -5.117592 -3.592462 -1.647639
C 2.913681 0.222799 0.173425
C 3.334786 1.537339 -0.090731
C 3.737409 -0.880995 -0.121891
C 4.591067 1.727834 -0.671401
C 4.983198 -0.640228 -0.701842
C 5.411474 0.652115 -0.979019
H 4.925522 2.739562 -0.886352
H 5.625238 -1.481884 -0.947889
H 6.383059 0.818197 -1.434236
C -0.532734 -3.634993 -0.784108
H 0.219692 -2.907286 -0.467788
C -3.722399 0.057137 0.880548
H -2.807288 0.391621 1.381202
C 2.472627 2.744833 0.225734
H 1.559412 2.389687 0.709937
C 3.301826 -2.315191 0.134508
H 2.370475 -2.301496 0.712338
C 2.693184 -0.024710 2.922989
H 2.417327 -0.096173 3.975429
H 3.471564 -0.765762 2.712589
H 3.130077 0.958310 2.725160
C -1.773756 -2.075152 2.817951
H -1.324757 -2.369054 3.767123
H -2.643603 -1.444590 3.028623
H -2.135743 -2.963742 2.295212
C 2.069960 3.478137 -1.060139
H 2.935637 3.982671 -1.505080
H 1.665117 2.787079 -1.804892
H 1.307284 4.235212 -0.844238
C 3.168975 3.709141 1.193157
H 3.447000 3.214737 2.129560
H 4.081777 4.123702 0.751750
H 2.505289 4.545925 1.433962
C 3.009588 -3.021291 -1.194523
H 2.232042 -2.490938 -1.751761
H 3.912524 -3.065396 -1.814184
H 2.676087 -4.049437 -1.010555
C 4.339108 -3.100481 0.945739
H 5.272470 -3.224412 0.386660
H 4.581423 -2.603524 1.890775
H 3.956999 -4.100592 1.174412
C -4.147954 1.155268 -0.103327
H -3.388600 1.318748 -0.871931
H -5.083833 0.879054 -0.602143
H -4.313340 2.098931 0.427918
C -4.819247 -0.123353 1.940515
H -5.790477 -0.306663 1.468940
H -4.619689 -0.963770 2.611845
H -4.913873 0.786134 2.542265
C -0.432479 -4.843280 0.155099
H -1.170432 -5.608416 -0.112019
H 0.563381 -5.295755 0.090929
H -0.608278 -4.555325 1.197410
C -0.204436 -4.024873 -2.228002
H -0.911717 -4.761656 -2.621996
H -0.218423 -3.141967 -2.871959
H 0.792299 -4.472609 -2.280022
C -0.722430 1.678418 -0.624042
C -1.078556 2.356077 0.556080
C -1.038427 2.331331 -1.807412
C -1.709901 3.586166 0.528131
C -1.678478 3.564986 -1.881681
F -0.693495 1.759905 -2.975925
C -2.014711 4.184154 -0.691466
F -2.052911 4.217195 1.659606
F 0.505014 -0.901984 -1.855446
H -1.902849 4.031725 -2.833448
H -0.864174 1.927572 1.535620

F -2.631896 5.367915 -0.694381

11

Final structure in terms of initial Cartesian coordinates:

C 0.000000 0.693576 0.683902
C 0.000000 -0.693576 0.683902
C 0.000000 -1.379333 -0.525905
C 0.000000 -0.696946 -1.729882
C 0.000000 0.696946 -1.729882
C 0.000000 1.379333 -0.525905
F 0.000000 1.355756 1.836589
F 0.000000 -1.355756 1.836589
F 0.000000 -2.713797 -0.493933
F 0.000000 2.713797 -0.493933
H 0.000000 -1.264574 -2.652604
H 0.000000 1.264574 -2.652604

IM_{1-11}

Final structure in terms of initial Cartesian coordinates:

Al -0.385380 -0.295496 -0.510662
N 0.621507 -1.651236 0.546935
N -2.056783 -0.948517 0.360607
C 0.155271 -2.679996 1.255283
C -2.243508 -2.013134 1.139765
C -1.206849 -2.879866 1.506419
H -1.474607 -3.741671 2.103573
C 2.044478 -1.440761 0.521863
C 2.645303 -0.767845 1.605945
C 2.800719 -1.850650 -0.589170
C 4.015312 -0.516769 1.553512
C 4.169608 -1.569051 -0.598857
C 4.776275 -0.908358 0.458145
H 4.493625 0.011994 2.372629
H 4.765372 -1.865752 -1.458297
H 5.839353 -0.690784 0.427821
C -3.182356 -0.094410 0.093949
C -4.074687 -0.419341 -0.946310
C -3.333283 1.089118 0.839463
C -5.130860 0.452537 -1.211216
C -4.410943 1.927405 0.541906
C -5.306496 1.615727 -0.470967
H -5.825033 0.220526 -2.014247
H -4.542933 2.843697 1.111654
H -6.135862 2.281179 -0.690117
C 1.820105 -0.270224 2.783985
H 0.901723 -0.864681 2.840599
C 2.188715 -2.590288 -1.767621
H 1.135058 -2.783630 -1.538742
C -3.880641 -1.655161 -1.811304
H -3.178305 -2.323751 -1.302856
C -2.364685 1.492814 1.941347
H -1.593284 0.718188 2.023103
C -3.625524 -2.318385 1.668311
H -4.027774 -1.454374 2.206090
H -4.316628 -2.524650 0.844417
H -3.605679 -3.179714 2.336793
C 1.125762 -3.665918 1.862378
H 1.707302 -3.188039 2.658571
H 0.597696 -4.523582 2.280900
H 1.843216 -4.011377 1.112202
C -3.253856 -1.260419 -3.155378
H -3.920628 -0.583098 -3.700995
H -2.298467 -0.745342 -3.008548
H -3.081303 -2.145703 -3.776801
C -5.182725 -2.431995 -2.035538
H -5.683197 -2.668684 -1.090856
H -5.887317 -1.865483 -2.653134
H -4.974069 -3.370888 -2.557924
C -1.670824 2.815678 1.595763
H -1.157131 2.751563 0.631813
H -2.396909 3.633756 1.531033
H -0.933302 3.079448 2.360704
C -3.066185 1.585995 3.301633
H -3.836087 2.365251 3.296056
H -3.547849 0.640550 3.570696
H -2.343049 1.836607 4.084929
C 2.244848 -1.734505 -3.038912
H 1.686313 -0.803332 -2.898705
H 3.279775 -1.477490 -3.291176
H 1.809648 -2.276589 -3.885489
C 2.870389 -3.945102 -1.996721
H 3.918931 -3.817176 -2.285889

H 2.846516 -4.565346 -1.095049
H 2.368242 -4.491469 -2.801624
C 2.541481 -0.417750 4.127064
H 3.391944 0.266972 4.206768
H 1.856208 -0.179263 4.946360
H 2.913687 -1.436807 4.275522
C 1.409936 1.189166 2.552617
H 2.292614 1.833020 2.484165
H 0.849705 1.301130 1.615585
H 0.777272 1.550315 3.372239
C 1.591864 3.172513 -0.439561
C 0.689750 3.414370 -1.468633
C 2.686588 2.350143 -0.664166
C 2.863457 1.776659 -1.919354
C 0.867346 2.848013 -2.717213
C 1.966089 2.023056 -2.944024
F 1.412327 3.728834 0.758248
F 3.932185 1.001091 -2.121733
F 3.570857 2.146833 0.309806
H 0.136143 3.045772 -3.491382
H 2.134932 1.556915 -3.907609
F -0.356282 4.205673 -1.213586

$TS_{1\text{-}11}$

Final structure in terms of initial Cartesian coordinates:

Al 0.10214 0.014944 0.814083
N -1.122537 -1.469239 0.501053
N 1.635581 -1.134437 0.408707
C -0.821826 -2.749402 0.721342
C 1.650632 -2.455386 0.590824
C 0.494925 -3.209945 0.839716
H 0.631562 -4.265093 1.035487
C -2.467508 -1.132924 0.119021
C -2.790255 -1.152762 -1.252024
C -3.406430 -0.741709 1.087344
C -4.086358 -0.800851 -1.629410
C -4.685901 -0.383137 0.658635
C -5.030017 -0.416564 -0.685225
H -4.358716 -0.819420 -2.681408
H -5.422293 -0.064827 1.392205
H -6.030824 -0.135275 -0.998281
C 2.870750 -0.474505 0.073023
C 3.737424 -0.048777 1.095866
C 3.159876 -0.226951 -1.281199
C 4.921859 0.594426 0.733232
C 4.367638 0.398475 -1.594941
C 5.246970 0.804139 -0.600054
H 5.599624 0.938201 1.510219
H 4.615613 0.584622 -2.636288
H 6.177832 1.297474 -0.862964
C -1.783544 -1.563091 -2.316940
H -0.795761 -1.614261 -1.847290
C -3.065729 -0.657500 2.565313
H -2.073704 -1.099824 2.714175
C 3.404453 -0.219117 2.569413
H 2.534389 -0.880288 2.652800
C 2.196460 -0.601985 -2.395745
H 1.228821 -0.828307 -1.940281
C 2.967245 -3.193474 0.557115
H 3.534086 -2.948109 -0.344730
H 3.585365 -2.889608 1.409104
H 2.809361 -4.271309 0.603990
C -1.938779 -3.753921 0.869102
H -2.617326 -3.729165 0.013210
H -1.543603 -4.763108 0.989309
H -2.537721 -3.497798 1.750718
C 3.024153 1.135006 3.183917
H 3.874796 1.824972 3.142100
H 2.188031 1.592383 2.645287
H 2.734677 1.013199 4.233240
C 4.553088 -0.855320 3.361248
H 4.880935 -1.802188 2.920123
H 5.422626 -0.191082 3.403225
H 4.238347 -1.048207 4.391787
C 1.983375 0.564630 -3.366262
H 1.720208 1.475794 -2.823347
H 2.883017 0.765331 -3.958106
H 1.174724 0.330890 -4.065642
C 2.659612 -1.856775 -3.144425
H 3.648380 -1.701135 -3.590888
H 2.721188 -2.722958 -2.477617
H 1.958123 -2.101806 -3.949925
C -3.000922 0.808137 3.015678
H -2.263098 1.367738 2.432421
H -3.975298 1.292619 2.885333

H -2.725038 0.872519 4.073710
C -4.060699 -1.439413 3.431315
H -5.060695 -0.995380 3.385200
H -4.146395 -2.482859 3.110729
H -3.742507 -1.426441 4.478641
C -2.102992 -2.955033 -2.878274
H -3.105076 -2.972633 -3.321713
H -1.382482 -3.226480 -3.657485
H -2.063100 -3.725864 -2.102944
C -1.705810 -0.543685 -3.458070
H -2.642086 -0.504368 -4.025256
H -1.489355 0.457951 -3.079013
H -0.912735 -0.831087 -4.157506
C -0.322734 2.103245 -0.336951
C 0.766459 2.988788 -0.372646
C -1.600193 2.673384 -0.305051
C -1.753729 4.034734 -0.098569
C 0.616394 4.342275 -0.175300
C -0.664155 4.889866 -0.048960
F -0.194468 0.886514 -1.123514
F -2.999078 4.512027 0.023947
F -2.672848 1.873505 -0.306605
H 1.504712 4.962447 -0.135013
H -0.825769 5.951665 0.086579
F 1.991463 2.440541 -0.454678

12

Final structure in terms of initial Cartesian coordinates:
Al -0.070773 -0.051905 -0.462814
N -1.546438 -0.239438 0.707217
N 1.232586 -0.839369 0.672122
C -1.402282 -0.464068 2.017369
C 1.038473 -1.034653 1.973901
C -0.179661 -0.770286 2.621778
H -0.195136 -0.920201 3.693223
C -2.875233 -0.220944 0.147638
C -3.501749 -1.449625 -0.140534
C -3.509824 1.002932 -0.125384
C -4.767578 -1.424374 -0.725467
C -4.778718 0.976673 -0.709094
C -5.405479 -0.223391 -1.011533
H -5.260189 -2.362538 -0.966768
H -5.278809 1.916230 -0.930307
H -6.390098 -0.225035 -1.469279
C 2.467960 -1.249535 0.051439
C 3.605931 -0.421666 0.102658
C 2.489682 -2.484834 -0.626448
C 4.774290 -0.870288 -0.517927
C 3.686867 -2.891339 -1.217642
C 4.823540 -2.095586 -1.166010
H 5.662376 -0.244545 -0.488174
H 3.729789 -3.849668 -1.726936
H 5.744608 -2.429185 -1.634309
C -2.835240 -2.789218 0.134603
H -1.942183 -2.617979 0.746518
C -2.860975 2.340593 0.181936
H -1.899222 2.146644 0.664026
C 3.627298 0.933308 0.793142
H 2.651749 1.103906 1.260940
C 1.279685 -3.406485 -0.687624
H 0.395729 -2.844803 -0.371662
C 2.165922 -1.555564 2.828877
H 1.778217 -1.991739 3.750136
H 2.767400 -2.296564 2.297855
H 2.827354 -0.724701 3.096562
C -2.629724 -0.455623 2.892896
H -2.352315 -0.471814 3.947180
H -3.242627 0.426690 2.688734
H -3.251499 -1.331622 2.678921
C 3.876191 2.059601 -0.220263
H 4.869409 1.955694 -0.671263
H 3.136306 2.049385 -1.024960
H 3.832984 3.034049 0.278384
C 4.706468 0.996741 1.884679
H 4.632700 0.168858 2.595598
H 5.707952 0.957124 1.442454
H 4.627461 1.936928 2.439657
C 1.015260 -3.899748 -2.112759
H 0.842788 -3.052710 -2.781516
H 1.850564 -4.492461 -2.500018
H 0.127297 -4.538520 -2.130650
C 1.451903 -4.580581 0.284027
H 2.326553 -5.183809 0.015438
H 1.586919 -4.231601 1.313625
H 0.570245 -5.230618 0.259162

C -2.607756 3.127184 -1.111257
H -2.122559 2.504931 -1.868246
H -3.551934 3.492650 -1.531494
H -1.964133 3.992751 -0.915096
C -3.701922 3.174119 1.156091
H -4.683346 3.410486 0.730777
H -3.864982 2.649326 2.102677
H -3.195783 4.119988 1.375155
C -3.750768 -3.744379 0.909651
H -4.611713 -4.050039 0.305841
H -3.201326 -4.651888 1.179591
H -4.134486 -3.290879 1.829413
C -2.378496 -3.433901 -1.179581
H -3.238403 -3.618293 -1.833958
H -1.673554 -2.786136 -1.708662
H -1.894368 -4.396972 -0.978836
C 0.421567 1.827903 -0.809870
C 0.634256 2.642578 0.288845
C 0.621685 2.449428 -2.039190
C 1.027181 3.972006 0.199659
F 0.450581 2.123930 1.526291
C 1.015875 3.772596 -2.190182
F 0.401633 1.738411 -3.158241
C 1.224222 4.541850 -1.047037
F 1.228075 4.680912 1.318634
F -0.291796 -0.951731 -1.852873
H 1.537435 5.578346 -1.107505
H 1.150324 4.184505 -3.183647

IM$_{1\text{-}11iso}$

Final structure in terms of initial Cartesian coordinates:

Al 0.276809 0.164097 0.378541
N -0.590154 -1.578893 0.788396
N 2.030680 -0.691925 0.761202
C -0.087212 -2.605660 1.473060
C 2.278251 -1.827562 1.412260
C 1.264524 -2.694838 1.834452
H 1.566258 -3.569202 2.395946
C -1.960308 -1.660082 0.353866
C -2.222059 -2.006905 -0.988689
C -3.001012 -1.337793 1.241362
C -3.554580 -2.100122 -1.392716
C -4.317636 -1.442098 0.787848
C -4.596506 -1.835297 -0.511748
H -3.787381 -2.369333 -2.417199
H -5.134807 -1.194068 1.459611
H -5.625421 -1.912709 -0.849936
C 3.133828 0.019853 0.177752
C 3.795147 1.029266 0.900469
C 3.481029 -0.268383 -1.155852
C 4.822782 1.731601 0.268259
C 4.518694 0.456259 -1.744476
C 5.190098 1.447790 -1.041134
H 5.340257 2.519004 0.810174
H 4.798813 0.243736 -2.773411
H 5.993781 2.003246 -1.515129
C -1.088569 -2.258138 -1.977435
H -0.339032 -1.471326 -1.811392
C -2.734394 -0.795048 2.635746
H -1.691641 -1.005387 2.897478
C 3.392052 1.394022 2.319471
H 2.700737 0.625671 2.682452
C 2.746806 -1.319227 -1.975744
H 2.014760 -1.815422 -1.327905
C 3.707515 -2.227465 1.687736
H 4.246822 -2.377443 0.746720
H 4.232227 -1.433108 2.227992
H 3.750318 -3.147384 2.272103
C -0.984042 -3.757734 1.862643
H -1.490483 -4.165232 0.982047
H -0.411023 -4.550007 2.345894
H -1.766387 -3.417642 2.548824
C 2.650052 2.737337 2.332681
H 3.305014 3.538055 1.969838
H 1.764049 2.706211 1.690221
H 2.333377 2.991711 3.349935
C 4.591616 1.438395 3.272928
H 5.163194 0.504750 3.253530
H 5.275440 2.253643 3.013687
H 4.250872 1.610707 4.298882
C 1.978794 -0.664248 -3.130335
H 1.248354 0.065457 -2.762574
H 2.664439 -0.145048 -3.809964
H 1.441523 -1.423968 -3.708785
C 3.698954 -2.396143 -2.506988

H 4.432702 -1.971707 -3.200813
H 4.248289 -2.882376 -1.695174
H 3.137665 -3.165948 -3.046643
C -2.916613 0.729524 2.629004
H -2.229508 1.204595 1.921044
H -3.936463 0.993089 2.327526
H -2.731190 1.146314 3.625030
C -3.622758 -1.436824 3.707078
H -4.672279 -1.151223 3.581356
H -3.569946 -2.530500 3.680213
H -3.311854 -1.102630 4.701910
C -0.397858 -3.611253 -1.756798
H -1.125349 -4.427767 -1.837183
H 0.369722 -3.768379 -2.524188
H 0.092791 -3.677658 -0.781995
C -1.536421 -2.151965 -3.436909
H -2.178451 -2.994475 -3.718760
H -2.077253 -1.221555 -3.625310
H -0.662275 -2.179275 -4.094178
C -1.584587 2.049712 -2.026674
C -0.775194 3.121672 -2.349724
C -2.489859 2.123235 -0.975234
C -2.573825 3.287663 -0.230207
C -0.863531 4.295913 -1.603297
C -1.757271 4.367854 -0.549741
F -1.485941 0.892236 -2.694000
F -3.423264 3.362661 0.792729
F -3.237414 1.072271 -0.652154
H -0.241200 5.155395 -1.822875
F -1.866628 5.478693 0.184480
H -0.067869 3.019059 -3.164198

TS$_{1\text{-}11iso}$

Final structure in terms of initial Cartesian coordinates:

Al -0.068258 -0.131552 0.806907
N -1.565917 -1.298235 0.341420
N 1.190152 -1.591878 0.508640
C -1.562414 -2.622292 0.484079
C 0.911518 -2.882777 0.682765
C -0.400265 -3.366653 0.747221
H -0.525861 -4.429093 0.908553
C -2.800379 -0.631192 0.017318
C -3.069237 -0.306378 -1.324747
C -3.685995 -0.264844 1.048823
C -4.269413 0.343573 -1.620147
C -4.862519 0.401848 0.704781
C -5.163551 0.694932 -0.618726
H -4.499922 0.587298 -2.653873
H -5.552313 0.700935 1.489702
H -6.087251 1.208468 -0.867202
C 2.519411 -1.189857 0.149957
C 3.405520 -0.706654 1.128800
C 2.874019 -1.205588 -1.216128
C 4.660993 -0.250092 0.717812
C 4.146153 -0.756552 -1.574064
C 5.036157 -0.278412 -0.618723
H 5.354798 0.134601 1.460841
H 4.440391 -0.771613 -2.620209
H 6.018118 0.075337 -0.917907
C -2.105543 -0.629819 -2.455278
H -1.167370 -0.980527 -2.015288
C -3.384893 -0.523966 2.516814
H -2.505909 -1.175262 2.580942
C 3.037018 -0.634050 2.600603
H 2.056817 -1.106209 2.730635
C 1.922405 -1.694731 -2.298718
H 0.934512 -1.828228 -1.846614
C 2.044032 -3.867581 0.842269
H 2.797078 -3.747496 0.060489
H 2.548098 -3.678425 1.797828
H 1.677157 -4.894546 0.837473
C -2.863433 -3.379656 0.378440
H -3.396270 -3.114674 -0.538760
H -2.690808 -4.455977 0.399367
H -3.520265 -3.110047 1.212518
C 2.917939 0.827167 3.052779
H 3.883027 1.339349 2.963560
H 2.184296 1.368719 2.446216
H 2.603140 0.877852 4.100351
C 4.043452 -1.390823 3.475551
H 4.151122 -2.433263 3.158803
H 5.034436 -0.926158 3.432056
H 3.719120 -1.380280 4.520941
C 1.783652 -0.677587 -3.436998
H 1.498281 0.306002 -3.055936

H 2.719404 -0.574916 -3.997377
H 1.015614 -1.014085 -4.142583
C 2.369295 -3.050603 -2.862076
H 3.372203 -2.976642 -3.297747
H 2.392591 -3.827067 -2.091946
H 1.682729 -3.379087 -3.649488
C -3.044058 0.793787 3.226345
H -2.208075 1.300766 2.734471
H -3.907443 1.468703 3.212854
H -2.772533 0.608105 4.271066
C -4.543395 -1.227202 3.235027
H -5.427437 -0.583101 3.286605
H -4.838194 -2.152884 2.730301
H -4.256142 -1.473257 4.262194
C -2.647959 -1.749281 -3.351503
H -3.600758 -1.456358 -3.807037
H -1.940470 -1.967909 -4.159054
H -2.814165 -2.673135 -2.788106
C -1.792040 0.621410 -3.284560
H -2.669262 0.952054 -3.851253
H -1.471595 1.444918 -2.641427
H -0.994203 0.410960 -4.003901
C 0.670624 1.849977 -0.275066
C 2.013832 2.237343 -0.387618
C -0.325082 2.837927 -0.253495
C 0.017076 4.165164 -0.106640
C 2.349775 3.582752 -0.267415
C 1.360295 4.541020 -0.131679
F 0.298523 0.625952 -1.045940
F -0.934562 5.088344 0.036382
F -1.609277 2.467340 -0.168483
H 3.385277 3.902591 -0.301649
F 1.659785 5.842606 -0.027406
H 2.787582 1.484489 -0.496427

13

Final structure in terms of initial Cartesian coordinates:

Al 0.095726 -0.596113 -0.768136
N -1.383471 -1.409398 0.101725
N 1.423803 -1.319538 0.391974
C -1.224559 -2.612651 0.671041
C 1.257501 -2.496571 0.984775
C 0.017649 -3.157390 1.015897
H 0.006647 -4.143168 1.462066
C -2.709771 -0.846880 0.068787
C -3.432955 -0.832925 -1.134576
C -3.231189 -0.275987 1.244878
C -4.689527 -0.223312 -1.143330
C -4.492500 0.314880 1.188848
C -5.219601 0.347048 0.004960
H -5.257840 -0.192989 -2.068985
H -4.906032 0.773476 2.083050
H -6.196475 0.820210 -0.022103
C 2.681462 -0.629101 0.465449
C 2.787681 0.452749 1.360751
C 3.721491 -0.964510 -0.417631
C 3.983959 1.168823 1.385961
C 4.896670 -0.208736 -0.361746
C 5.033314 0.842566 0.533951
H 4.092584 2.005913 2.068284
H 5.712185 -0.444750 -1.040355
H 5.953938 1.417544 0.561742
C -2.866683 -1.413691 -2.418254
H -1.972505 -1.992705 -2.173061
C -2.437183 -0.212145 2.541441
H -1.564816 -0.870035 2.456473
C 1.631993 0.836252 2.271376
H 0.705449 0.506015 1.788555
C 3.589185 -2.062231 -1.461845
H 2.661796 -2.612524 -1.273525
C 2.432141 -3.174192 1.644903
H 3.077498 -3.618388 0.878400
H 3.040380 -2.458110 2.202986
H 2.096146 -3.966341 2.314839
C -2.451299 -3.420347 1.016696
H -3.150469 -3.432094 0.175410
H -2.176244 -4.442524 1.278270
H -2.979151 -2.972280 1.864631
C 1.521928 2.348185 2.475665
H 2.357258 2.741998 3.064781
H 1.499798 2.871475 1.513324
H 0.602959 2.586583 3.019278
C 1.727842 0.099510 3.612982
H 1.687012 -0.986248 3.474425
H 2.666408 0.343988 4.123398

H 0.897781 0.385908 4.268271
C 3.474134 -1.451826 -2.865107
H 2.587610 -0.818021 -2.946597
H 4.362678 -0.854692 -3.100267
H 3.386464 -2.243900 -3.615854
C 4.760607 -3.050506 -1.405829
H 5.695884 -2.574201 -1.718939
H 4.915139 -3.449387 -0.397995
H 4.577479 -3.889113 -2.084861
C -1.920744 1.215579 2.761347
H -1.302845 1.545681 1.921786
H -2.758381 1.916566 2.849547
H -1.326774 1.273469 3.680970
C -3.251756 -0.672275 3.756122
H -4.074203 0.018160 3.969816
H -3.683572 -1.667170 3.607185
H -2.613873 -0.706043 4.645156
C -3.850944 -2.359433 -3.113846
H -4.745158 -1.829900 -3.459343
H -3.377752 -2.812968 -3.990432
H -4.174541 -3.163538 -2.444835
C -2.440295 -0.282968 -3.363686
H -3.316014 0.284599 -3.699639
H -1.760791 0.420946 -2.871397
H -1.927679 -0.688719 -4.240939
C 0.291986 1.368922 -0.906817
C 1.538693 1.909716 -1.283112
C -0.710330 2.302224 -0.678826
C -0.522002 3.676601 -0.771741
C 1.768605 3.278472 -1.396883
C 0.727900 4.155553 -1.133838
F 0.323547 -1.343699 -2.254878
F -1.526848 4.521270 -0.533148
F -1.953329 1.904349 -0.348021
H 2.734582 3.677884 -1.685210
F 0.899561 5.477015 -1.231710
H 2.373880 1.244590 -1.491201

14

Final structure in terms of initial Cartesian coordinates:
C 0.000000 0.000000 1.113478
C 0.000000 1.205123 0.422762
C 0.000000 1.191249 -0.966074
C 0.000000 0.000000 -1.674542
C 0.000000 -1.191249 -0.966074
C 0.000000 -1.205123 0.422762
F 0.000000 0.000000 2.441094
F 0.000000 2.353860 1.092131
F 0.000000 2.357223 -1.610273
F 0.000000 -2.357223 -1.610273
F 0.000000 -2.353860 1.092131
H 0.000000 0.000000 -2.757162

$IM_{1\text{-}14}$

Final structure in terms of initial Cartesian coordinates:
Al -0.560963 0.425681 -0.696284
N -2.246681 0.748315 0.314530
N 0.326159 1.712414 0.529732
C -2.543416 1.770428 1.115276
C -0.236357 2.704791 1.217775
C -1.613848 2.764412 1.457813
H -1.977666 3.598653 2.043666
C -3.250802 -0.245724 0.052076
C -3.135553 -1.504939 0.677641
C -4.274082 0.018459 -0.876416
C -4.110327 -2.465816 0.406298
C -5.222666 -0.977344 -1.118108
C -5.153889 -2.204136 -0.473743
H -4.047312 -3.440862 0.877678
H -6.020561 -0.791769 -1.831808
H -5.900783 -2.966858 -0.671708
C 1.744763 1.513403 0.609478
C 2.609938 2.064761 -0.349921
C 2.223682 0.659897 1.627255
C 3.973135 1.771364 -0.256900
C 3.594866 0.407958 1.687246
C 4.466962 0.961513 0.756392
H 4.657580 2.182258 -0.995440
H 3.986484 -0.248802 2.457593
H 5.528346 0.740805 0.808610
C -1.983853 -1.808464 1.627596
H -1.075109 -1.375256 1.186605

C -4.320269 1.310616 -1.677214
H -3.677230 2.047855 -1.185718
C 2.108811 2.954493 -1.475082
H 1.042338 3.142816 -1.307606
C 1.274812 0.036301 2.642779
H 0.303657 -0.085998 2.148778
C 0.627038 3.823754 1.752846
H 1.511215 3.442581 2.269146
H 0.982706 4.429812 0.910922
H 0.062889 4.467588 2.429433
C -3.931812 1.888633 1.700617
H -4.233114 0.944210 2.163915
H -3.970320 2.683486 2.446421
H -4.664764 2.105991 0.916994
C 2.250739 2.258588 -2.834772
H 3.303858 2.029703 -3.039632
H 1.681508 1.323142 -2.860932
H 1.884734 2.907680 -3.637546
C 2.832729 4.306647 -1.490582
H 2.777670 4.806231 -0.518110
H 3.892427 4.184027 -1.739552
H 2.390359 4.966512 -2.243871
C 1.735815 -1.340038 3.127072
H 1.991584 -2.004520 2.299560
H 2.609636 -1.263481 3.783775
H 0.937829 -1.811853 3.710386
C 1.049778 0.953073 3.854044
H 2.006936 1.204187 4.325859
H 0.544528 1.882533 3.579673
H 0.425536 0.445177 4.597706
C -3.747286 1.064638 -3.080000
H -2.725584 0.674222 -3.024828
H -4.358985 0.330600 -3.616544
H -3.736059 1.992757 -3.661670
C -5.728817 1.906960 -1.771334
H -6.390797 1.278521 -2.375973
H -6.190415 2.023281 -0.785071
H -5.688440 2.890576 -2.249813
C -2.197075 -1.154670 2.999247
H -3.144515 -1.491069 3.436467
H -1.390191 -1.434538 3.686337
H -2.213720 -0.062028 2.935074
C -1.701480 -3.302564 1.787013
H -2.503966 -3.813071 2.331038
H -1.572273 -3.786063 0.814217
H -0.777579 -3.439646 2.356992
C 1.898393 -2.780672 -0.416220
C 3.190495 -2.281258 -0.310967
C 1.133122 -2.533879 -1.546096
C 1.671936 -1.768169 -2.571194
C 3.711467 -1.530940 -1.356125
C 2.960131 -1.264417 -2.489544
F 1.380960 -3.495053 0.583591
F 0.918358 -1.505612 -3.635213
F -0.110031 -3.000040 -1.612623
H 3.369987 -0.668105 -3.295046
F 3.916239 -2.535971 0.777481
F 4.967949 -1.092901 -1.264302

TS$_{1\text{-}14}$

Final structure in terms of initial Cartesian coordinates:
Al -0.026511 -0.135749 0.767436
N -1.451289 -1.414848 0.396909
N 1.328894 -1.490417 0.418591
C -1.341050 -2.736837 0.542160
C 1.151972 -2.801111 0.591451
C -0.113139 -3.388157 0.718629
H -0.143968 -4.457339 0.880340
C -2.747040 -0.862034 0.094940
C -3.085585 -0.646317 -1.253487
C -3.621713 -0.505259 1.135957
C -4.344561 -0.119296 -1.542070
C -4.860495 0.042519 0.798075
C -5.229390 0.222899 -0.527868
H -4.628752 0.043921 -2.577819
H -5.545845 0.334035 1.589720
H -6.200631 0.643310 -0.770505
C 2.642789 -1.009420 0.080780
C 3.532016 -0.606423 1.090316
C 2.980674 -0.903131 -1.282199
C 4.782839 -0.116057 0.710716
C 4.248010 -0.421875 -1.610799
C 5.145918 -0.030427 -0.625772
H 5.480446 0.210083 1.477864
H 4.533607 -0.343070 -2.656077

H 6.124588 0.350245 -0.901663
C -2.118515 -0.955859 -2.384738
H -1.124048 -1.073417 -1.946867
C -3.246994 -0.652184 2.601715
H -2.317581 -1.230063 2.663700
C 3.164432 -0.642890 2.564297
H 2.209583 -1.171265 2.667958
C 2.016750 -1.309757 -2.386510
H 1.018114 -1.386561 -1.946310
C 2.358909 -3.702287 0.690162
H 3.050125 -3.543542 -0.140516
H 2.910183 -3.463101 1.607156
H 2.062055 -4.751185 0.721006
C -2.589867 -3.585124 0.553063
H -3.216790 -3.379818 -0.318176
H -2.339463 -4.646080 0.578495
H -3.190365 -3.340715 1.436353
C 2.974041 0.783487 3.097900
H 3.908283 1.349930 3.013818
H 2.203165 1.316555 2.532612
H 2.678800 0.764128 4.152441
C 4.207290 -1.391569 3.402812
H 4.400156 -2.395640 3.011263
H 5.161208 -0.853947 3.420791
H 3.863308 -1.485869 4.437714
C 1.950391 -0.267976 -3.507635
H 1.746947 0.728888 -3.108708
H 2.884723 -0.228808 -4.077946
H 1.152516 -0.530882 -4.210313
C 2.374616 -2.686187 -2.962141
H 3.397803 -2.686691 -3.354875
H 2.300339 -3.473755 -2.206174
H 1.695813 -2.945379 -3.781932
C -2.984021 0.726997 3.221781
H -2.202872 1.263749 2.673852
H -3.894355 1.336634 3.199118
H -2.666992 0.625633 4.265284
C -4.317278 -1.400209 3.405535
H -5.247270 -0.824650 3.459608
H -4.555218 -2.373110 2.963424
H -3.972209 -1.564552 4.431284
C -2.471732 -2.271609 -3.087926
H -3.485346 -2.229608 -3.502884
H -1.774719 -2.462374 -3.911485
H -2.420047 -3.122689 -2.401458
C -2.042401 0.193760 -3.394913
H -2.964633 0.275221 -3.980483
H -1.872686 1.147294 -2.889141
H -1.222426 0.022069 -4.099408
C 0.137101 2.003134 -0.381651
C 1.348528 2.702985 -0.268737
C -1.044050 2.761030 -0.408834
C -1.018274 4.109243 -0.111780
C 1.348647 4.052556 0.028283
C 0.174598 4.792398 0.089396
F 0.156715 0.836142 -1.224601
F -2.181619 4.765243 -0.044972
F -2.215939 2.120739 -0.532532
H 0.187933 5.854633 0.292723
F 2.497558 2.015157 -0.260897
F 2.525221 4.654418 0.233625

15

Final structure in terms of initial Cartesian coordinates:

Al -0.107410 -0.121311 -0.387743
N -1.617060 -0.271137 0.740121
N 1.103415 -1.101884 0.694063
C -1.520776 -0.613716 2.028558
C 0.864291 -1.384012 1.973740
C -0.339741 -1.068766 2.623891
H -0.391093 -1.302786 3.678797
C -2.926638 -0.082665 0.164381
C -3.651758 -1.218461 -0.246971
C -3.444390 1.213096 -0.002618
C -4.893666 -1.025655 -0.852410
C -4.694284 1.354916 -0.610665
C -5.414572 0.249204 -1.038524
H -5.460668 -1.890146 -1.187340
H -5.104484 2.351782 -0.750337
H -6.382280 0.378604 -1.513451
C 2.313940 -1.579082 0.068921
C 3.522790 -0.870153 0.212791
C 2.244182 -2.758873 -0.698629
C 4.665147 -1.384507 -0.404445
C 3.419137 -3.236945 -1.281786

C 4.623084 -2.561500 -1.137378
H 5.606257 -0.850078 -0.305073
H 3.389303 -4.156317 -1.859424
H 5.525547 -2.948905 -1.600245
C -3.116611 -2.631995 -0.077029
H -2.230725 -2.596114 0.567106
C -2.693155 2.452900 0.447669
H -1.762770 2.129929 0.922588
C 3.643179 0.434265 0.986211
H 2.677790 0.652719 1.455256
C 0.956463 -3.549966 -0.881198
H 0.120040 -2.950081 -0.510021
C 1.923872 -2.081679 2.787766
H 1.515633 -2.429906 3.736628
H 2.348646 -2.928053 2.241729
H 2.744489 -1.387955 2.994860
C -2.761994 -0.572671 2.882806
H -2.512872 -0.714513 3.934634
H -3.285384 0.379134 2.755747
H -3.457360 -1.361731 2.576504
C 3.990908 1.591285 0.038048
H 4.976641 1.432572 -0.413209
H 3.261177 1.682100 -0.770523
H 4.021258 2.538276 0.587980
C 4.711674 0.357373 2.086702
H 4.566432 -0.494040 2.757861
H 5.711912 0.261495 1.651102
H 4.699566 1.272638 2.686677
C 0.684793 -3.842806 -2.360073
H 0.595399 -2.909814 -2.921661
H 1.480349 -4.449684 -2.804864
H -0.250402 -4.400648 -2.467242
C 0.996636 -4.844940 -0.060553
H 1.819635 -5.490026 -0.388328
H 1.133982 -4.640351 1.006609
H 0.060794 -5.401456 -0.181627
C -2.344737 3.340200 -0.755277
H -1.905521 2.755483 -1.568140
H -3.242978 3.832811 -1.144849
H -1.629256 4.118553 -0.465090
C -3.485517 3.254525 1.487435
H -4.429760 3.619923 1.069490
H -3.721898 2.652709 2.370585
H -2.905577 4.124006 1.813601
C -4.137268 -3.561356 0.590981
H -5.002974 -3.733492 -0.057125
H -3.680332 -4.535619 0.791795
H -4.506612 -3.154578 1.538065
C -2.680893 -3.201193 -1.432600
H -3.535771 -3.255459 -2.116530
H -1.911018 -2.573789 -1.890588
H -2.283725 -4.215289 -1.306320
C 0.544594 1.737853 -0.573758
C 0.825883 2.433138 0.592166
C 0.799934 2.417703 -1.755725
C 1.339430 3.721474 0.608980
F 0.590815 1.829522 1.781157
C 1.316980 3.710152 -1.772900
F 0.526318 1.846542 -2.935316
C 1.595069 4.374379 -0.587977
F 1.603942 4.321229 1.774972
F 1.541750 4.313932 -2.943380
F -0.365790 -0.870288 -1.857307
H 2.001214 5.378724 -0.598548

IM$_{1\text{-}14ortho}$

Final structure in terms of initial Cartesian coordinates:

Al 0.355007 0.333748 0.648245
N 2.004252 1.005613 -0.235729
N -0.645524 1.765024 -0.300904
C 2.209222 2.188761 -0.812804
C -0.174830 2.913772 -0.784317
C 1.192143 3.140782 -0.981764
H 1.479162 4.100318 -1.391910
C 3.099238 0.079408 -0.135997
C 3.119298 -1.041853 -0.992621
C 4.076368 0.256859 0.860309
C 4.180606 -1.940046 -0.873318
C 5.114774 -0.672900 0.942847
C 5.178479 -1.754351 0.076432
H 4.222414 -2.810111 -1.519674
H 5.878474 -0.554429 1.706435
H 5.993055 -2.467939 0.154283
C -2.041068 1.462402 -0.435650
C -2.953312 1.781262 0.581874

C -2.439775 0.744289 -1.585421
C -4.286079 1.391027 0.420637
C -3.781971 0.385523 -1.705525
C -4.702326 0.705940 -0.712634
H -5.006810 1.628627 1.199600
H -4.110955 -0.171794 -2.576869
H -5.742636 0.413666 -0.822537
C 2.012060 -1.272452 -2.014428
H 1.057681 -1.060083 -1.510030
C 3.983818 1.370812 1.892277
H 3.280022 2.126519 1.528961
C -2.533246 2.512718 1.845008
H -1.497061 2.844977 1.713839
C -1.436913 0.391141 -2.676963
H -0.458079 0.276919 -2.196974
C -1.136743 4.027653 -1.127175
H -1.968485 3.668086 -1.737931
H -1.567894 4.425678 -0.200785
H -0.628582 4.838947 -1.650561
C 3.584782 2.545008 -1.326373
H 3.967581 1.750471 -1.974344
H 3.559753 3.481927 -1.884003
H 4.292866 2.646888 -0.498103
C -2.572361 1.570091 3.053708
H -3.577357 1.154160 3.190243
H -1.878499 0.734095 2.924477
H -2.298663 2.106577 3.968621
C -3.398223 3.753379 2.099618
H -3.424954 4.414556 1.227266
H -4.430945 3.475832 2.336792
H -3.006813 4.319648 2.950834
C -1.760809 -0.920121 -3.395760
H -1.935204 -1.740198 -2.697133
H -2.645668 -0.820862 -4.034626
H -0.924746 -1.196825 -4.047068
C -1.300285 1.520411 -3.708839
H -2.276584 1.757069 -4.147894
H -0.887361 2.432458 -3.270283
H -0.629140 1.210381 -4.517489
C 3.417793 0.808647 3.203965
H 2.437112 0.348003 3.044609
H 4.087484 0.041104 3.607939
H 3.313417 1.601682 3.952181
C 5.326004 2.066184 2.144837
H 6.035363 1.398409 2.644420
H 5.790990 2.409928 1.214800
H 5.181188 2.932847 2.797493
C 2.132119 -0.320375 -3.212029
H 3.113317 -0.432217 -3.688032
H 1.366052 -0.552422 -3.961082
H 2.003668 0.726983 -2.921436
C 1.918737 -2.719509 -2.499309
H 2.781923 -2.996480 -3.114557
H 1.844739 -3.416787 -1.660061
H 1.022191 -2.838450 -3.115214
C -1.442327 -3.023858 -0.152003
C -2.723374 -2.501905 -0.030836
C -0.610915 -3.059342 0.958026
C -1.061053 -2.589970 2.182327
C -3.184542 -2.003148 1.177298
C -2.341160 -2.061997 2.275342
F -0.993001 -3.464638 -1.329933
F -0.246117 -2.582693 3.230707
F 0.639061 -3.491033 0.829798
F -3.516526 -2.511237 -1.105830
H -4.177954 -1.580161 1.259636
F -2.743975 -1.591137 3.457587

$TS_{1\text{-}14ortho}$

Final structure in terms of initial Cartesian coordinates:
Al 0.132344 -0.145704 0.816140
N -1.019021 -1.683629 0.486652
N 1.719463 -1.211048 0.396168
C -0.653860 -2.950977 0.682583
C 1.800826 -2.532607 0.555863
C 0.684283 -3.348024 0.789995
H 0.872789 -4.398407 0.967285
C -2.381544 -1.407797 0.118048
C -2.715089 -1.428529 -1.250610
C -3.329263 -1.069300 1.097882
C -4.028675 -1.130688 -1.613769
C -4.626909 -0.763222 0.683053
C -4.980439 -0.797455 -0.658337
H -4.308340 -1.150638 -2.663864
H -5.370796 -0.487781 1.426436

H -5.995356 -0.557506 -0.960268
C 2.918252 -0.483541 0.069033
C 3.760767 -0.024871 1.097846
C 3.195494 -0.206535 -1.282296
C 4.910096 0.683983 0.744299
C 4.369192 0.485401 -1.586835
C 5.224734 0.926015 -0.586068
H 5.568563 1.053040 1.526211
H 4.609446 0.694676 -2.625718
H 6.128496 1.470467 -0.842225
C -1.700699 -1.778082 -2.329802
H -0.708553 -1.803412 -1.867109
C -2.982466 -0.988488 2.574583
H -1.966571 -1.377795 2.710354
C 3.439690 -0.233438 2.569139
H 2.602536 -0.936714 2.643897
C 2.258273 -0.623345 -2.404792
H 1.305921 -0.919020 -1.956248
C 3.152643 -3.202634 0.510167
H 3.702461 -2.920660 -0.391476
H 3.758307 -2.876063 1.362597
H 3.049517 -4.287481 0.547183
C -1.719354 -4.012210 0.812235
H -2.392303 -4.011701 -0.048362
H -1.274928 -5.001656 0.924094
H -2.336475 -3.796187 1.691913
C 2.997687 1.091231 3.206041
H 3.811446 1.824310 3.164339
H 2.133026 1.511689 2.682539
H 2.726733 0.940850 4.256545
C 4.620119 -0.826058 3.348173
H 4.993934 -1.747644 2.890489
H 5.455516 -0.120048 3.400910
H 4.317568 -1.052300 4.375527
C 1.974541 0.542883 -3.357565
H 1.652821 1.427769 -2.802656
H 2.861390 0.808807 -3.942795
H 1.184343 0.269499 -4.063659
C 2.802323 -1.833461 -3.172503
H 3.781045 -1.608392 -3.611362
H 2.916489 -2.705056 -2.519828
H 2.120935 -2.109060 -3.985315
C -2.994804 0.471340 3.047556
H -2.295908 1.079402 2.464868
H -3.995776 0.903403 2.935618
H -2.710396 0.535406 4.103268
C -3.927364 -1.836915 3.434303
H -4.950494 -1.448030 3.400198
H -3.957546 -2.878612 3.098689
H -3.603752 -1.821762 4.479926
C -1.972640 -3.166507 -2.924145
H -2.974604 -3.208803 -3.366042
H -1.245209 -3.392230 -3.711351
H -1.903278 -3.954850 -2.168806
C -1.667658 -0.729261 -3.446643
H -2.607787 -0.711952 -4.008547
H -1.488393 0.270551 -3.044178
H -0.867710 -0.968259 -4.156337
C -0.384199 1.934749 -0.317282
C 0.649035 2.881314 -0.356932
C -1.693474 2.431841 -0.297576
C -1.941693 3.780096 -0.102828
C 0.425848 4.229641 -0.171458
C -0.883492 4.679799 -0.060095
F -0.182017 0.721851 -1.101335
F -3.199046 4.210196 0.011875
F -2.717754 1.574068 -0.294757
F 1.904592 2.412432 -0.426262
F -1.148008 5.982465 0.087716
H 1.256350 4.923828 -0.128987

16

Final structure in terms of initial Cartesian coordinates:

Al -0.149711 -0.160284 -0.444681
N -1.637739 -0.042431 0.718294
N 0.938216 -1.239965 0.674255
C -1.563238 -0.332315 2.020758
C 0.691212 -1.426163 1.969465
C -0.444385 -0.918143 2.620965
H -0.504143 -1.092125 3.687077
C -2.921209 0.285271 0.148237
C -3.793718 -0.766572 -0.194180
C -3.268543 1.626781 -0.085115
C -5.010132 -0.445938 -0.796654
C -4.499463 1.897800 -0.687702

C -5.364566 0.874268 -1.046539
H -5.689287 -1.245729 -1.080190
H -4.778995 2.930455 -0.880535
H -6.314639 1.102651 -1.519837
C 2.062300 -1.896805 0.052179
C 3.354967 -1.344621 0.140991
C 1.822161 -3.088738 -0.660733
C 4.406452 -2.027155 -0.475601
C 2.910923 -3.737526 -1.245759
C 4.194835 -3.217193 -1.156098
H 5.410256 -1.614747 -0.415972
H 2.748875 -4.669196 -1.780151
H 5.027425 -3.735864 -1.621462
C -3.443001 -2.227291 0.045515
H -2.537998 -2.273413 0.661954
C -2.354178 2.779979 0.287105
H -1.461707 2.362861 0.761440
C 3.662730 -0.040644 0.861421
H 2.741516 0.326383 1.327223
C 0.439563 -3.715682 -0.772887
H -0.304066 -2.986220 -0.439038
C 1.664832 -2.215490 2.807315
H 1.203287 -2.522193 3.746349
H 2.028032 -3.097443 2.274420
H 2.535103 -1.592917 3.038516
C -2.768711 -0.079932 2.889426
H -2.515277 -0.191014 3.944037
H -3.170055 0.921923 2.713096
H -3.566146 -0.789548 2.643778
C 4.158738 1.022112 -0.130552
H 5.108116 0.710825 -0.580463
H 3.440722 1.184727 -0.938596
H 4.327683 1.974194 0.384573
C 4.721738 -0.228419 1.958391
H 4.472553 -1.039644 2.648143
H 5.697749 -0.462356 1.519553
H 4.832989 0.694476 2.536348
C 0.099806 -4.077240 -2.221745
H 0.125224 -3.185589 -2.852780
H 0.795159 -4.818666 -2.628528
H -0.904268 -4.508656 -2.275847
C 0.329912 -4.941137 0.142863
H 1.057158 -5.709326 -0.144057
H 0.514198 -4.677468 1.189907
H -0.671431 -5.380358 0.074176
C -1.924273 3.556642 -0.964807
H -1.571003 2.883327 -1.750633
H -2.764178 4.133515 -1.368905
H -1.117220 4.258124 -0.723414
C -3.012180 3.726538 1.298484
H -3.914503 4.186053 0.880839
H -3.297576 3.203655 2.216688
H -2.320385 4.531538 1.567119
C -4.554179 -2.971312 0.796085
H -5.464247 -3.043205 0.191280
H -4.232219 -3.991549 1.028025
H -4.818266 -2.474205 1.735234
C -3.130441 -2.926527 -1.282785
H -4.007969 -2.915209 -1.939477
H -2.303900 -2.429743 -1.798329
H -2.858242 -3.973171 -1.101841
C 0.742351 1.576065 -0.736532
C 1.127143 2.283421 0.392057
C 1.078790 2.186764 -1.939413
C 1.800645 3.495626 0.367956
F 0.833477 1.771295 1.607884
C 1.755187 3.396492 -2.048206
F 0.716883 1.590410 -3.085240
C 2.113111 4.037758 -0.872833
F 2.161082 4.116565 1.493210
F -0.548965 -0.953446 -1.859322
H 1.992694 3.830908 -3.011419
F 2.766504 5.199660 -0.908944

$IM_{1\text{-}14meta}$

Final structure in terms of initial Cartesian coordinates:

Al 0.583772 -0.281243 0.362168
N 2.379191 0.577226 0.486461
N -0.205032 1.466458 0.885265
C 2.718421 1.737790 1.045881
C 0.388216 2.538417 1.410165
C 1.775776 2.643673 1.551950
H 2.157574 3.546906 2.009395
C 3.417322 -0.236645 -0.083882
C 3.570478 -0.241812 -1.485705

C 4.207970 -1.054496 0.742674
C 4.581704 -1.032731 -2.033412
C 5.200868 -1.834603 0.146548
C 5.399510 -1.815207 -1.226659
H 4.725466 -1.051308 -3.108559
H 5.820981 -2.474580 0.768014
H 6.178483 -2.425535 -1.673149
C -1.601717 1.549068 0.559770
C -2.576200 1.139204 1.486563
C -1.958540 1.984324 -0.731910
C -3.917973 1.179045 1.098825
C -3.312682 2.020688 -1.069454
C -4.289225 1.629042 -0.161770
H -4.684294 0.852582 1.796573
H -3.606941 2.358459 -2.060520
H -5.336464 1.654014 -0.443708
C 2.659376 0.591482 -2.378565
H 1.654610 0.563906 -1.935912
C 3.958015 -1.165893 2.238539
H 3.337974 -0.318873 2.549825
C -2.205576 0.647490 2.876214
H -1.156768 0.907233 3.057705
C -0.917348 2.422322 -1.752972
H 0.070729 2.374417 -1.281506
C -0.455522 3.711571 1.848343
H -0.998808 4.132555 0.996461
H -1.210025 3.388183 2.573236
H 0.160807 4.491277 2.297218
C 4.175578 2.128256 1.145559
H 4.673628 2.023894 0.177286
H 4.277538 3.156725 1.493928
H 4.699829 1.468141 1.844552
C -2.330306 -0.878831 2.953290
H -3.345288 -1.199249 2.691158
H -1.633874 -1.364506 2.262295
H -2.110128 -1.235153 3.965372
C -3.049026 1.313467 3.969634
H -3.024932 2.405489 3.892331
H -4.095969 0.997655 3.915064
H -2.673365 1.030051 4.957735
C -0.896871 1.485018 -2.966287
H -0.669407 0.452260 -2.677411
H -1.866870 1.491194 -3.478044
H -0.139003 1.814875 -3.686222
C -1.144415 3.870482 -2.204431
H -2.096347 3.975262 -2.736110
H -1.159452 4.559113 -1.354082
H -0.345877 4.185657 -2.884202
C 3.168820 -2.448700 2.535824
H 2.223469 -2.467306 1.983252
H 3.748839 -3.329438 2.238216
H 2.949239 -2.528197 3.605841
C 5.249002 -1.128756 3.063745
H 5.854262 -2.026576 2.901488
H 5.868424 -0.260621 2.815167
H 5.010536 -1.086490 4.131035
C 3.092111 2.062326 -2.426191
H 4.119239 2.148634 -2.799109
H 2.437270 2.628620 -3.098983
H 3.041679 2.533023 -1.439732
C 2.529604 0.025994 -3.793538
H 3.459977 0.134302 -4.361668
H 2.254777 -1.032902 -3.773207
H 1.752644 0.569567 -4.339235
C -1.752141 -2.156164 -1.917977
C -2.879273 -1.411223 -2.224469
C -1.660743 -2.878090 -0.735387
C -2.710056 -2.818377 0.171780
C -3.922221 -1.380906 -1.313161
C -3.838915 -2.057295 -0.104458
F -0.717441 -2.176604 -2.759914
F -2.628249 -3.482986 1.320596
F -0.585199 -3.602916 -0.457660
F -4.832654 -1.993213 0.782480
F -5.040043 -0.707678 -1.591874
H -2.944155 -0.861880 -3.155179

$TS_{1\text{-}14meta}$

Final structure in terms of initial Cartesian coordinates:
Al 0.197788 -0.203001 -0.780319
N 1.856102 -1.122433 -0.321879
N -0.823595 -1.830763 -0.481922
C 2.051118 -2.436702 -0.427081
C -0.354249 -3.068998 -0.633135
C 1.015610 -3.353442 -0.670514

H 1.299868 -4.388387 -0.806906
C 2.978197 -0.270678 -0.019580
C 3.193250 0.125598 1.312908
C 3.800428 0.194492 -1.062989
C 4.281959 0.955479 1.588260
C 4.865961 1.035564 -0.738921
C 5.116722 1.406598 0.575391
H 4.471011 1.259651 2.614270
H 5.507420 1.409948 -1.532187
H 5.954916 2.056125 0.808404
C -2.202738 -1.631000 -0.138018
C -3.147800 -1.316656 -1.129183
C -2.554319 -1.674477 1.228105
C -4.462979 -1.061337 -0.730343
C -3.884671 -1.432764 1.573438
C -4.835262 -1.125722 0.605695
H -5.205206 -0.806341 -1.482458
H -4.179417 -1.469852 2.618553
H -5.862680 -0.926954 0.894796
C 2.287072 -0.316097 2.451005
H 1.401283 -0.785070 2.014522
C 3.540997 -0.143895 -2.522871
H 2.774746 -0.926243 -2.567789
C -2.784569 -1.215730 -2.600178
H -1.741339 -1.531609 -2.716079
C -1.533200 -1.976519 2.315938
H -0.536006 -1.873859 1.876711
C -1.324850 -4.212148 -0.806904
H -2.128403 -4.181069 -0.068383
H -1.796551 -4.125353 -1.793385
H -0.814799 -5.174393 -0.747864
C 3.451117 -2.986546 -0.302894
H 3.930292 -2.630621 0.613109
H 3.443157 -4.076777 -0.307066
H 4.066835 -2.633211 -1.136848
C -2.892357 0.237325 -3.080733
H -3.924165 0.597378 -2.996386
H -2.250820 0.896763 -2.486510
H -2.589586 0.316367 -4.130091
C -3.653481 -2.138200 -3.463638
H -3.607551 -3.175970 -3.118008
H -4.703493 -1.827286 -3.443293
H -3.319794 -2.106409 -4.505643
C -1.627182 -0.989684 3.484909
H -1.577880 0.044344 3.134023
H -2.556360 -1.121098 4.049888
H -0.797805 -1.160545 4.180311
C -1.673847 -3.415405 2.830037
H -2.678348 -3.584399 3.234500
H -1.498536 -4.151130 2.039832
H -0.950071 -3.604283 3.629881
C 2.996963 1.087483 -3.259922
H 2.081686 1.457813 -2.787407
H 3.736469 1.896192 -3.247286
H 2.774599 0.843860 -4.304297
C 4.792925 -0.673517 -3.232750
H 5.559743 0.103496 -3.317969
H 5.239204 -1.521839 -2.703857
H 4.540617 -0.996679 -4.247618
C 2.974316 -1.357776 3.341351
H 3.892921 -0.950571 3.778732
H 2.312293 -1.656452 4.161826
H 3.239761 -2.257768 2.777367
C 1.813491 0.880460 3.284046
H 2.636877 1.316427 3.860196
H 1.394354 1.660500 2.643588
H 1.043612 0.563613 3.994999
C -0.857660 1.635621 0.299610
C -2.251294 1.809089 0.354160
C -0.028843 2.768106 0.275786
C -0.569105 4.017533 0.057045
C -2.766667 3.078450 0.161707
C -1.951441 4.193788 0.020344
F -0.335784 0.519680 1.110120
F 0.230018 5.071816 -0.098023
F 1.300488 2.597741 0.234400
F -2.467652 5.414599 -0.143038
H -2.928159 0.967734 0.457179
F -4.090975 3.262341 0.140421

17

Final structure in terms of initial Cartesian coordinates:

Al -0.175324 -0.185901 -0.349947
N -1.709437 0.036528 0.734030
N 0.684084 -1.492407 0.714493

C -1.784176 -0.409911 1.990018
C 0.318709 -1.774535 1.964485
C -0.795678 -1.197589 2.592582
H -0.966468 -1.478137 3.623208
C -2.870978 0.635171 0.123197
C -3.857062 -0.212467 -0.420162
C -2.983289 2.034810 0.044583
C -4.942948 0.371978 -1.072098
C -4.093522 2.570630 -0.613436
C -5.062690 1.752375 -1.175320
H -5.706654 -0.266309 -1.508531
H -4.197340 3.650497 -0.681282
H -5.914613 2.189083 -1.687478
C 1.754899 -2.239050 0.100085
C 3.094196 -1.842708 0.279447
C 1.419161 -3.351589 -0.696949
C 4.093900 -2.618584 -0.311333
C 2.457746 -4.099448 -1.254341
C 3.786137 -3.745820 -1.059862
H 5.133483 -2.330225 -0.179720
H 2.218887 -4.973683 -1.853463
H 4.579509 -4.340552 -1.502264
C -3.768935 -1.729158 -0.333717
H -2.965624 -1.993827 0.363443
C -1.974941 2.979425 0.678524
H -1.164100 2.381959 1.104568
C 3.499801 -0.600296 1.058196
H 2.598317 -0.139499 1.475952
C -0.019430 -3.777533 -0.956181
H -0.689339 -3.000750 -0.574041
C 1.121827 -2.775215 2.755068
H 0.655855 -2.971734 3.720574
H 1.223954 -3.713913 2.203019
H 2.133701 -2.394974 2.920266
C -3.026734 -0.119749 2.791768
H -2.869439 -0.351462 3.845485
H -3.326926 0.925562 2.686905
H -3.858311 -0.729444 2.420438
C 4.173895 0.419468 0.127740
H 5.118240 0.020533 -0.259268
H 3.537387 0.669725 -0.725708
H 4.399657 1.340366 0.677016
C 4.455245 -0.925244 2.215437
H 4.045774 -1.668343 2.905568
H 5.406221 -1.316513 1.838321
H 4.674369 -0.017492 2.786390
C -0.293190 -3.914941 -2.457450
H -0.120148 -2.962599 -2.964001
H 0.343534 -4.680023 -2.913910
H -1.333628 -4.210185 -2.625273
C -0.348992 -5.081050 -0.218431
H 0.296010 -5.898377 -0.560187
H -0.216155 -4.977732 0.863549
H -1.388746 -5.370741 -0.405490
C -1.374762 3.941348 -0.354234
H -0.940898 3.400757 -1.199897
H -2.136584 4.626594 -0.742144
H -0.587806 4.547378 0.108028
C -2.614072 3.777857 1.823382
H -3.434658 4.403004 1.454113
H -3.017767 3.123659 2.602135
H -1.871244 4.436092 2.285452
C -5.066543 -2.350945 0.196850
H -5.886365 -2.230184 -0.518951
H -4.928402 -3.424768 0.358015
H -5.382530 -1.899375 1.143024
C -3.409858 -2.328499 -1.698764
H -4.183891 -2.088493 -2.436957
H -2.454254 -1.938505 -2.059226
H -3.341586 -3.420345 -1.623849
C 0.920125 1.423596 -0.602964
C 1.392510 2.136644 0.483370
C 1.295037 1.883002 -1.876034
C 2.203694 3.257510 0.367190
F 1.072193 1.736081 1.736722
C 2.098397 3.002166 -2.018028
C 2.558753 3.694081 -0.901496
F 2.659918 3.901125 1.441465
F -0.544131 -0.787258 -1.870759
F 3.338972 4.763244 -1.040317
F 2.459315 3.453142 -3.223546
H 0.961456 1.369463 -2.772996

[1]**1B**

Final structure in terms of initial Cartesian coordinates:

N -0.102276 1.209248 0.532770
N 0.102276 -1.209248 0.532770
C -0.344373 1.163208 1.874662
C 0.344373 -1.163208 1.874662
C 0.000000 0.000000 2.555099
H 0.000000 0.000000 3.639086
C 0.000000 2.502710 -0.095889
C -1.112577 3.062082 -0.737533
C 1.237148 3.167502 -0.025004
C -0.957359 4.315743 -1.336693
C 1.342852 4.418022 -0.632646
C 0.254584 4.989291 -1.284586
H -1.800087 4.768575 -1.851560
H 2.285620 4.954509 -0.599028
H 0.355543 5.963585 -1.753474
C 0.000000 -2.502710 -0.095889
C 1.112577 -3.062082 -0.737533
C -1.237148 -3.167502 -0.025004
C 0.957359 -4.315743 -1.336693
C -1.342852 -4.418022 -0.632646
C -0.254584 -4.989291 -1.284586
H 1.800087 -4.768575 -1.851560
H -2.285620 -4.954509 -0.599028
H -0.355543 -5.963585 -1.753474
C -2.438746 2.328234 -0.830989
H -2.429498 1.518405 -0.093081
C 2.435877 2.495588 0.626954
H 2.074876 1.920260 1.487817
C 2.438746 -2.328234 -0.830989
H 2.429498 -1.518405 -0.093081
C -2.435877 -2.495588 0.626954
H -2.074876 -1.920260 1.487817
C 0.926527 -2.356231 2.575517
H 1.137910 -2.122771 3.620743
H 0.248152 -3.215202 2.533298
H 1.859170 -2.664835 2.088611
C -0.926527 2.356231 2.575517
H -1.137910 2.122771 3.620743
H -0.248152 3.215202 2.533298
H -1.859170 2.664835 2.088611
C 2.581717 -1.687361 -2.217045
H 2.596893 -2.460710 -2.994257
H 1.738207 -1.014791 -2.403366
H 3.515172 -1.116526 -2.282413
C 3.635908 -3.229333 -0.513428
H 3.520650 -3.730520 0.453767
H 3.774965 -4.002311 -1.276480
H 4.553445 -2.632818 -0.484347
C -3.065655 -1.506225 -0.363931
H -2.317944 -0.801584 -0.749461
H -3.480301 -2.047401 -1.222229
H -3.876285 -0.941691 0.111401
C -3.484301 -3.481406 1.144440
H -3.993355 -3.996392 0.322889
H -3.039745 -4.238613 1.798523
H -4.249875 -2.943895 1.712240
C 3.065655 1.506225 -0.363931
H 2.317944 0.801584 -0.749461
H 3.480301 2.047401 -1.222229
H 3.876285 0.941691 0.111401
C 3.484301 3.481406 1.144440
H 3.993355 3.996392 0.322889
H 3.039745 4.238613 1.798523
H 4.249875 2.943895 1.712240
C -3.635908 3.229333 -0.513428
H -3.774965 4.002311 -1.276480
H -4.553445 2.632818 -0.484347
H -3.520650 3.730520 0.453767
C -2.581717 1.687361 -2.217045
H -2.596893 2.460710 -2.994257
H -1.738207 1.014791 -2.403366
H -3.515172 1.116526 -2.282413
B 0.000000 0.000000 -0.294718

$^{1}IM_{1B\_A-14}$

Final structure in terms of initial Cartesian coordinates:
B 0.106561 -0.524286 0.367773
N -1.042940 -1.310176 0.962520
N 1.357144 -1.075469 1.015018
C -0.976627 -2.235799 1.933013
C 1.433378 -2.029874 1.954836
C 0.264351 -2.595699 2.452977
H 0.322603 -3.350172 3.226581
C -2.338785 -1.019528 0.382694
C -2.660109 -1.598130 -0.857027

C -3.231563 -0.187683 1.075778
C -3.935021 -1.356198 -1.371421
C -4.494045 0.022947 0.518649
C -4.849817 -0.566038 -0.686809
H -4.214505 -1.784855 -2.328067
H -5.204384 0.664330 1.030779
H -5.836692 -0.393550 -1.105631
C 2.584394 -0.538372 0.468904
C 3.178192 0.572305 1.085819
C 3.120418 -1.128896 -0.684175
C 4.314844 1.118916 0.489083
C 4.267826 -0.556427 -1.235596
C 4.856830 0.563150 -0.663453
H 4.781505 1.995050 0.929866
H 4.694325 -0.985051 -2.138863
H 5.739783 1.004474 -1.115440
C -1.645658 -2.437434 -1.618417
H -0.677583 -1.936789 -1.482867
C -2.807264 0.555658 2.332268
H -2.053992 -0.043353 2.857644
C 2.572062 1.213665 2.322661
H 1.921358 0.475786 2.806850
C 2.474577 -2.325487 -1.364487
H 1.657199 -2.689621 -0.730873
C 2.773678 -2.483906 2.457687
H 2.658183 -3.266804 3.208786
H 3.381996 -2.862213 1.629957
H 3.322705 -1.642847 2.894427
C -2.225032 -2.896928 2.449046
H -1.969375 -3.701935 3.140046
H -2.858454 -2.171025 2.969510
H -2.817356 -3.300370 1.622484
C 1.689712 2.394224 1.901789
H 2.294031 3.158408 1.399102
H 0.923318 2.049240 1.199903
H 1.200831 2.849488 2.770500
C 3.624232 1.642994 3.348520
H 4.303303 0.821463 3.601079
H 4.229240 2.477823 2.980309
H 3.134168 1.979251 4.267697
C 1.862143 -1.910242 -2.707017
H 1.131633 -1.110405 -2.563522
H 2.639086 -1.544976 -3.388814
H 1.369682 -2.767475 -3.181157
C 3.466632 -3.479106 -1.551095
H 4.278929 -3.199532 -2.230415
H 3.917968 -3.785345 -0.601298
H 2.959053 -4.347524 -1.983683
C -2.130339 1.867992 1.916758
H -1.276031 1.652293 1.265128
H -2.833378 2.500582 1.362264
H -1.778422 2.423830 2.793361
C -3.953218 0.811320 3.312805
H -4.666188 1.541477 2.916407
H -4.503639 -0.106837 3.546368
H -3.557096 1.222128 4.246523
C -1.535182 -3.863324 -1.062028
H -2.506533 -4.370545 -1.104496
H -0.824471 -4.445578 -1.659774
H -1.183037 -3.881718 -0.025925
C -1.924338 -2.491904 -3.121113
H -2.811344 -3.094756 -3.348511
H -2.068959 -1.491118 -3.535862
H -1.076491 -2.955652 -3.634770
C -0.599783 1.541166 -1.919822
C 0.711946 1.989131 -1.822204
C -1.642073 2.323230 -1.451743
C -1.361085 3.560123 -0.884304
C 0.964814 3.224447 -1.252832
C -0.063954 4.029536 -0.779573
F -0.865480 0.381145 -2.509057
F -2.382247 4.287736 -0.418315
H 0.144624 4.990839 -0.327348
F -2.892939 1.864014 -1.491894
F 1.711912 1.217986 -2.238939
F 2.235794 3.623072 -1.128562

[1]TS$_{1B\text{-}A\text{-}14}$

Final structure in terms of initial Cartesian coordinates:
B -0.088072 -0.435700 0.361616
N -1.352968 -1.034752 0.907221
N 1.054875 -1.046488 1.108486
C -1.444077 -1.912824 1.921541
C 0.962725 -1.917437 2.130056
C -0.286384 -2.340798 2.564222

H -0.358486 -3.039978 3.386779
C -2.582129 -0.666822 0.231864
C -2.870792 -1.286286 -0.993059
C -3.433231 0.274260 0.829716
C -4.074721 -0.947043 -1.613146
C -4.625851 0.573858 0.171256
C -4.946465 -0.032675 -1.036312
H -4.331005 -1.398462 -2.565813
H -5.307849 1.300491 0.600631
H -5.877143 0.216669 -1.536895
C 2.381105 -0.714693 0.625395
C 3.043882 0.393686 1.170803
C 2.951534 -1.523541 -0.366434
C 4.287035 0.731227 0.634823
C 4.211265 -1.163903 -0.847381
C 4.865334 -0.037215 -0.368388
H 4.812629 1.603407 1.011775
H 4.672676 -1.761073 -1.629323
H 5.832945 0.242426 -0.773561
C -1.923331 -2.300617 -1.617691
H -0.903970 -1.972908 -1.386022
C -3.031006 1.016790 2.094909
H -2.432071 0.343943 2.721100
C 2.428890 1.212491 2.294198
H 1.691052 0.583466 2.807525
C 2.232581 -2.727050 -0.954496
H 1.339757 -2.932567 -0.351425
C 2.209261 -2.442252 2.780919
H 1.957503 -3.096749 3.616437
H 2.810163 -2.998115 2.053952
H 2.832511 -1.616801 3.139877
C -2.781516 -2.439226 2.363815
H -2.647276 -3.244153 3.088159
H -3.373718 -1.642930 2.826155
H -3.356704 -2.806815 1.509940
C 1.684069 2.419685 1.718671
H 2.381699 3.079380 1.189404
H 0.926687 2.095359 0.998828
H 1.194340 2.994068 2.512950
C 3.459313 1.649448 3.339108
H 4.049111 0.802771 3.706686
H 4.153411 2.392030 2.933017
H 2.952167 2.112728 4.191109
C 1.763683 -2.427169 -2.383986
H 1.106754 -1.554719 -2.410083
H 2.623469 -2.219004 -3.031119
H 1.228663 -3.291823 -2.794552
C 3.107111 -3.986341 -0.927880
H 3.973614 -3.881752 -1.588825
H 3.480525 -4.204678 0.078377
H 2.532056 -4.850982 -1.274598
C -2.131287 2.204442 1.729391
H -1.239691 1.856447 1.198162
H -2.658726 2.902912 1.071908
H -1.815645 2.739631 2.631901
C -4.223579 1.476202 2.936322
H -4.774794 2.281806 2.440738
H -4.924013 0.657594 3.135594
H -3.870749 1.868758 3.894741
C -2.120228 -3.701738 -1.023896
H -3.156523 -4.036029 -1.151531
H -1.466915 -4.420981 -1.530468
H -1.878959 -3.735472 0.043760
C -2.034171 -2.353798 -3.141949
H -2.973829 -2.815418 -3.466137
H -1.965071 -1.352682 -3.574801
H -1.217495 -2.959112 -3.547688
C -0.032402 1.199570 -1.600313
C 1.304452 1.600947 -1.780399
C -0.954932 2.234106 -1.369598
C -0.535900 3.524739 -1.111328
C 1.702893 2.897682 -1.530120
C 0.797367 3.901614 -1.205075
F -0.475783 0.145199 -2.340917
F -1.469344 4.435341 -0.792953
H 1.117504 4.916252 -1.012221
F -2.258252 1.926105 -1.278433
F 2.216402 0.670539 -2.100362
F 3.012025 3.184410 -1.606871

^{1}15B

Final structure in terms of initial Cartesian coordinates:
N 1.331361 0.480794 0.824184
N -1.206027 0.681254 0.818260
C 1.298304 0.722100 2.133055

C -1.120164 0.996250 2.109314
C 0.091326 0.926069 2.798788
H 0.104726 1.142204 3.857666
C 2.576946 0.663861 0.110117
C 2.860866 1.960161 -0.363587
C 3.480534 -0.394130 -0.078797
C 4.042615 2.163463 -1.074553
C 4.646192 -0.142462 -0.809387
C 4.926167 1.118364 -1.313066
H 4.275024 3.158845 -1.443817
H 5.351896 -0.952546 -0.972702
H 5.838519 1.291966 -1.875641
C -2.504729 0.738934 0.175778
C -3.412320 -0.327870 0.356897
C -2.864521 1.872033 -0.577987
C -4.674026 -0.234801 -0.232646
C -4.141929 1.913178 -1.143857
C -5.044241 0.874814 -0.978161
H -5.379603 -1.050652 -0.101136
H -4.432001 2.784162 -1.724778
H -6.031657 0.930303 -1.426356
C 1.963027 3.152873 -0.075540
H 1.046257 2.792174 0.397722
C 3.282507 -1.779862 0.510760
H 2.364189 -1.771022 1.104643
C -3.097668 -1.585550 1.154726
H -2.085957 -1.501150 1.558614
C -1.954992 3.068807 -0.808123
H -0.995596 2.878597 -0.322599
C -2.333604 1.506774 2.836316
H -2.104181 1.672300 3.889264
H -2.652785 2.453952 2.388068
H -3.178276 0.822409 2.751170
C 2.580284 0.791678 2.915935
H 2.399617 1.203084 3.909631
H 2.981497 -0.221748 3.027528
H 3.340362 1.387922 2.406410
C -3.161165 -2.821065 0.244893
H -4.195748 -3.023962 -0.054288
H -2.570061 -2.680241 -0.663596
H -2.785821 -3.704071 0.773900
C -4.060121 -1.792208 2.334090
H -4.034270 -0.968703 3.052531
H -5.092846 -1.897352 1.985054
H -3.795985 -2.709238 2.870696
C -1.694655 3.246461 -2.309302
H -1.218112 2.353113 -2.720187
H -2.629895 3.423429 -2.851410
H -1.042634 4.107066 -2.486648
C -2.549793 4.349332 -0.208851
H -3.493475 4.617170 -0.696244
H -2.746451 4.244840 0.863224
H -1.855678 5.185696 -0.346080
C 3.146651 -2.838945 -0.589415
H 2.347330 -2.586661 -1.291054
H 4.080098 -2.923289 -1.157253
H 2.930179 -3.819409 -0.151200
C 4.443339 -2.155922 1.444085
H 5.376271 -2.273995 0.882497
H 4.617667 -1.395399 2.211174
H 4.233976 -3.107970 1.942489
C 2.641949 4.120716 0.902586
H 3.574807 4.512879 0.482506
H 1.984445 4.970516 1.114534
H 2.881441 3.633638 1.853525
C 1.562428 3.878268 -1.361580
H 2.427942 4.345556 -1.844525
H 1.102980 3.180475 -2.064843
H 0.842444 4.673692 -1.135377
C -0.072675 -1.292695 -0.539441
C 0.019911 -2.323806 0.388027
C -0.233285 -1.694823 -1.863280
C -0.010810 -3.666431 0.046145
F 0.142558 -2.021673 1.700283
C -0.272729 -3.041117 -2.226915
F -0.359121 -0.806186 -2.852059
C -0.157147 -4.043707 -1.278531
F 0.088544 -4.593354 1.007011
F -0.423773 -3.358462 -3.516290
F 0.120477 1.099506 -1.165000
H -0.187919 -5.087969 -1.564585
B 0.039388 0.264824 -0.052419

[1]IM$_{1B_B1-14}$

Final structure in terms of initial Cartesian coordinates:

N	-1.931457	0.581898	0.734009
N	0.336764	1.308646	1.107363
C	-2.331855	1.314608	1.791787
C	-0.083702	2.173454	2.046044
C	-1.427476	2.170452	2.411409
H	-1.764133	2.813506	3.214259
C	-2.934093	-0.203440	0.046180
C	-3.010925	-1.586754	0.285486
C	-3.811331	0.458712	-0.827308
C	-4.049727	-2.287473	-0.331936
C	-4.828455	-0.287791	-1.423111
C	-4.959705	-1.645921	-1.162913
H	-4.141185	-3.356254	-0.171145
H	-5.524108	0.196386	-2.101171
H	-5.761086	-2.211824	-1.628572
C	1.756194	1.160315	0.874314
C	2.420183	2.068078	0.038433
C	2.413535	0.076265	1.486997
C	3.785247	1.868904	-0.184387
C	3.778906	-0.073895	1.239406
C	4.460972	0.812738	0.411868
H	4.324248	2.545242	-0.842380
H	4.313974	-0.909902	1.677666
H	5.518776	0.665905	0.217633
C	-1.962982	-2.302250	1.126119
H	-1.002379	-1.881904	0.799991
C	-3.584836	1.916255	-1.197447
H	-3.185009	2.441368	-0.321749
C	1.685948	3.199620	-0.663944
H	0.717302	3.340743	-0.172648
C	1.648851	-0.912343	2.358161
H	0.706161	-1.122328	1.832050
C	0.885284	3.106943	2.714923
H	1.734624	2.555311	3.128586
H	1.289473	3.821767	1.990440
H	0.389273	3.659934	3.514442
C	-3.747181	1.227517	2.292084
H	-4.048301	0.183002	2.415268
H	-3.845603	1.749066	3.245672
H	-4.440180	1.673927	1.570899
C	1.390519	2.818386	-2.118564
H	2.327522	2.664829	-2.667642
H	0.802371	1.896756	-2.157444
H	0.829925	3.615460	-2.619170
C	2.446290	4.527651	-0.592344
H	2.720966	4.787037	0.435848
H	3.368138	4.493524	-1.182223
H	1.830051	5.335738	-0.999125
C	2.374324	-2.244937	2.553510
H	2.713691	-2.676793	1.611453
H	3.243519	-2.130706	3.212060
H	1.694994	-2.959562	3.029315
C	1.311401	-0.333917	3.740752
H	2.226280	-0.004897	4.247770
H	0.619902	0.510381	3.692218
H	0.841625	-1.104357	4.361799
C	-2.515581	1.981751	-2.297721
H	-1.611549	1.440403	-1.994072
H	-2.892859	1.511303	-3.212993
H	-2.256193	3.021776	-2.526111
C	-4.857185	2.649457	-1.623698
H	-5.233821	2.277406	-2.582183
H	-5.656105	2.544344	-0.881478
H	-4.645661	3.715365	-1.752420
C	-2.112510	-2.041324	2.630895
H	-3.099839	-2.359089	2.987443
H	-1.357290	-2.612541	3.183481
H	-1.974090	-0.986849	2.890225
C	-1.904907	-3.806964	0.865648
H	-2.795074	-4.323211	1.244076
H	-1.801760	-4.019023	-0.202119
H	-1.035151	-4.229510	1.377569
C	1.451020	-2.474440	-1.028338
C	2.802048	-2.168505	-1.139333
C	0.509810	-1.792126	-1.781447
C	0.936934	-0.787754	-2.637574
C	3.202114	-1.158650	-1.999175
C	2.275960	-0.461793	-2.759309
F	1.051196	-3.409377	-0.162414
F	0.021418	-0.108746	-3.330185
F	-0.774194	-2.117633	-1.691345
H	2.593733	0.332900	-3.422065
F	3.704315	-2.838706	-0.416305
F	4.505290	-0.880147	-2.096575
B	-0.524614	0.457616	0.213111

^{1}**TS$_{1B_B1\text{-}14}$**

Final structure in terms of initial Cartesian coordinates:

N -2.109836 -0.510769 -0.464807
N -0.046745 -1.690140 0.205648
C -2.480893 -1.683595 -1.157804
C -0.752015 -2.889316 0.079545
C -1.879080 -2.862135 -0.745368
H -2.281926 -3.807045 -1.102235
C -3.082299 0.523594 -0.231812
C -3.086654 1.681847 -1.020698
C -4.026747 0.317814 0.794355
C -4.074637 2.640594 -0.770437
C -4.993754 1.299344 1.003272
C -5.022073 2.452448 0.223349
H -4.097622 3.547054 -1.369723
H -5.735723 1.167773 1.784294
H -5.783746 3.206454 0.398079
C 1.389734 -1.664393 0.322683
C 1.956388 -1.244378 1.534186
C 2.170079 -2.018341 -0.796752
C 3.337605 -1.032791 1.570047
C 3.547406 -1.813415 -0.700481
C 4.122913 -1.293809 0.456251
H 3.799417 -0.672882 2.485601
H 4.184690 -2.048726 -1.545829
H 5.193367 -1.111866 0.488819
C -2.066664 1.942102 -2.114835
H -1.391126 1.083165 -2.163869
C -3.925385 -0.903912 1.694664
H -3.622991 -1.753818 1.074319
C 1.124602 -1.093717 2.798929
H 0.147874 -1.550145 2.606028
C 1.539822 -2.651970 -2.036386
H 0.857842 -3.427212 -1.668585
C -0.260372 -4.098880 0.810103
H 0.810407 -4.272078 0.644903
H -0.406915 -4.002621 1.895299
H -0.809212 -4.984531 0.480885
C -3.379638 -1.565941 -2.347958
H -2.850368 -1.091141 -3.187473
H -3.703064 -2.556335 -2.677207
H -4.267050 -0.958356 -2.138284
C 0.886705 0.371032 3.171294
H 1.837848 0.904617 3.293385
H 0.310636 0.883864 2.398015
H 0.334731 0.440812 4.115336
C 1.760413 -1.857189 3.967196
H 1.958615 -2.900144 3.700404
H 2.706653 -1.402813 4.279214
H 1.089182 -1.842617 4.831892
C 0.693973 -1.694157 -2.892543
H 0.044225 -1.050972 -2.292573
H 1.334701 -1.049598 -3.498141
H 0.059009 -2.278191 -3.567480
C 2.562677 -3.352035 -2.933299
H 3.236648 -2.631530 -3.409421
H 3.168324 -4.073509 -2.375994
H 2.042036 -3.888389 -3.732519
C -2.830618 -0.676490 2.747739
H -1.871328 -0.416880 2.283992
H -3.108045 0.151031 3.410499
H -2.686437 -1.575128 3.358366
C -5.242304 -1.275225 2.377008
H -5.535082 -0.529405 3.124102
H -6.057488 -1.373280 1.653275
H -5.131385 -2.230533 2.898980
C -2.740162 2.096617 -3.483417
H -3.401790 2.969553 -3.501335
H -1.985369 2.235004 -4.264401
H -3.341183 1.217503 -3.735934
C -1.226358 3.182000 -1.783641
H -1.839221 4.089894 -1.819056
H -0.788102 3.106912 -0.784889
H -0.414388 3.296079 -2.510100
C 1.567467 1.734588 -0.256286
C 2.577572 1.298604 -1.081544
C 1.829049 2.552288 0.821497
C 3.139079 2.936884 1.076680
C 3.884946 1.685623 -0.811317
C 4.182482 2.494646 0.274380
F 0.208940 0.915093 -0.176164
F 3.389030 3.745909 2.111356
F 0.839638 3.007155 1.600955
H 5.204011 2.785739 0.485475
F 2.328422 0.545471 -2.158932
F 4.863947 1.249571 -1.611302

B -0.800788 -0.519629 0.044639

^{1}**IM**$_{\mathbf{1B\_B2\text{-}14}}$

Final structure in terms of initial Cartesian coordinates:

N 1.829146 -0.029953 1.074557
N -0.542472 -0.244849 1.691949
C 2.194191 -0.181249 2.442365
C -0.179507 -0.372788 3.059693
C 1.172498 -0.345258 3.366030
H 1.452850 -0.445206 4.410318
C 2.841253 0.201375 0.082095
C 2.993703 1.495831 -0.449601
C 3.633922 -0.875106 -0.345319
C 3.995479 1.693753 -1.401393
C 4.625404 -0.627500 -1.296975
C 4.811358 0.646691 -1.815222
H 4.140779 2.678970 -1.831765
H 5.251120 -1.443641 -1.646020
H 5.585808 0.824328 -2.555438
C -1.921317 -0.153176 1.303900
C -2.652382 -1.335850 1.093752
C -2.495486 1.115862 1.123144
C -3.956261 -1.223541 0.610481
C -3.799931 1.180788 0.623366
C -4.520530 0.023848 0.357841
H -4.540471 -2.120574 0.425431
H -4.258283 2.151501 0.451527
H -5.528151 0.091879 -0.040181
C 2.093335 2.638318 0.001049
H 1.069003 2.246602 0.032419
C 3.354601 -2.291916 0.129201
H 2.841302 -2.236918 1.094979
C -2.022586 -2.692305 1.372237
H -1.240445 -2.544976 2.124711
C -1.771715 2.392587 1.523388
H -0.780175 2.118334 1.901080
C -1.265819 -0.486103 4.079614
H -1.928782 0.389162 4.072069
H -1.909038 -1.361403 3.914233
H -0.827244 -0.571487 5.076491
C 3.641481 -0.116227 2.812828
H 4.113273 0.805210 2.447302
H 3.741137 -0.144055 3.900652
H 4.224928 -0.949766 2.398344
C -1.339065 -3.242557 0.118001
H -2.076105 -3.434443 -0.670551
H -0.607501 -2.527776 -0.268841
H -0.819819 -4.181978 0.336005
C -3.020859 -3.707191 1.934681
H -3.557622 -3.305236 2.800104
H -3.761537 -4.005322 1.184954
H -2.493331 -4.613759 2.247192
C -1.580376 3.357616 0.350593
H -1.028701 2.895047 -0.470813
H -2.543687 3.697717 -0.044679
H -1.022773 4.241329 0.681789
C -2.524938 3.097904 2.660310
H -3.509086 3.440453 2.322098
H -2.677852 2.435092 3.517024
H -1.962806 3.973998 2.999809
C 2.401237 -2.974949 -0.859897
H 1.477992 -2.401515 -0.975224
H 2.865809 -3.050035 -1.849921
H 2.144212 -3.984569 -0.520618
C 4.622085 -3.127529 0.323000
H 5.110635 -3.349243 -0.631634
H 5.348764 -2.616833 0.963476
H 4.369344 -4.086169 0.786833
C 2.448097 3.120162 1.413496
H 3.486853 3.469272 1.447062
H 1.800529 3.957583 1.698430
H 2.323031 2.329780 2.159066
C 2.087225 3.822338 -0.966375
H 3.040274 4.363227 -0.944237
H 1.894803 3.501945 -1.993724
H 1.302755 4.528275 -0.675709
C -0.781103 0.853481 -2.379111
C -2.163669 0.963571 -2.386030
C -0.167603 -0.384665 -2.520829
C -0.956601 -1.513249 -2.681554
C -2.930949 -0.183947 -2.548904
C -2.341137 -1.426719 -2.700595
F -0.022881 1.938332 -2.235429
F -0.357324 -2.700571 -2.792468
F 1.158493 -0.467773 -2.472800

H -2.949134 -2.314505 -2.822893
F -2.746725 2.152469 -2.253212
F -4.257476 -0.060338 -2.574541
B 0.463927 -0.067909 0.712758

[1]**TS$_{1B-B2-14}$**

Final structure in terms of initial Cartesian coordinates:

N 2.198339 0.550283 0.516612
N -0.056535 1.149873 1.328765
C 2.770547 1.418943 1.492017
C 0.530448 1.914953 2.376983
C 1.911743 2.034499 2.388970
H 2.353854 2.663654 3.155642
C 3.062255 -0.267884 -0.293172
C 3.391402 0.141879 -1.591761
C 3.570941 -1.455781 0.264272
C 4.235492 -0.681294 -2.344027
C 4.417853 -2.238945 -0.517599
C 4.746967 -1.856811 -1.815010
H 4.495453 -0.390988 -3.358673
H 4.823809 -3.161273 -0.114259
H 5.403700 -2.481009 -2.413542
C -1.478747 1.179814 1.099387
C -2.294402 0.180006 1.653908
C -2.002439 2.207587 0.296592
C -3.638929 0.153100 1.274211
C -3.352905 2.145015 -0.049455
C -4.160222 1.110796 0.412222
H -4.288460 -0.624801 1.668247
H -3.776780 2.906098 -0.697826
H -5.202232 1.061563 0.109741
C 2.850483 1.422297 -2.203340
H 2.325771 1.977702 -1.418529
C 3.138670 -1.892872 1.654092
H 2.964797 -0.989870 2.251546
C -1.788952 -0.792310 2.709289
H -0.737531 -0.556480 2.910549
C -1.117383 3.354997 -0.168742
H -0.269380 3.414650 0.523134
C -0.372796 2.546320 3.384176
H -1.113312 3.209986 2.917883
H -0.935702 1.797685 3.957187
H 0.217558 3.135593 4.089644
C 4.248767 1.632921 1.465349
H 4.584702 2.020985 0.494283
H 4.531228 2.352642 2.237118
H 4.808389 0.704072 1.639669
C -1.869641 -2.256853 2.269499
H -2.888899 -2.520942 1.962951
H -1.198765 -2.471129 1.437046
H -1.591228 -2.914040 3.100669
C -2.578656 -0.605890 4.014144
H -2.607280 0.441232 4.329499
H -3.614254 -0.942367 3.893064
H -2.128645 -1.198246 4.817436
C -0.549236 3.103876 -1.570037
H -0.072698 2.121502 -1.635522
H -1.346679 3.130342 -2.319853
H 0.191048 3.871578 -1.823720
C -1.839291 4.704629 -0.116862
H -2.633453 4.768078 -0.868199
H -2.287158 4.882597 0.866056
H -1.130969 5.513013 -0.323897
C 1.808709 -2.653193 1.556569
H 1.055002 -2.074589 1.013135
H 1.946642 -3.592901 1.009577
H 1.417558 -2.887170 2.553108
C 4.186401 -2.733420 2.385019
H 4.316903 -3.714691 1.916591
H 5.160198 -2.233307 2.406659
H 3.867363 -2.908103 3.417142
C 3.974602 2.319511 -2.733569
H 4.497423 1.849439 -3.573409
H 3.562657 3.268945 -3.090582
H 4.715181 2.536107 -1.957173
C 1.844224 1.101697 -3.315781
H 2.332429 0.550231 -4.127432
H 1.020488 0.489507 -2.936456
H 1.427097 2.024045 -3.734680
C -1.352047 -1.200718 -1.481941
C -2.366162 -0.424119 -2.002893
C -1.599773 -2.487823 -1.053527
C -2.888184 -2.998816 -1.134981
C -3.649729 -0.949023 -2.082516
C -3.929549 -2.232739 -1.638949

F -0.112944 -0.505450 -0.931916
F -3.116641 -4.245458 -0.710875
F -0.613788 -3.250886 -0.560409
H -4.935069 -2.630976 -1.691778
F -2.129939 0.810109 -2.460229
F -4.627999 -0.184718 -2.578235
B 0.799127 0.484416 0.434011

[1]**IM'$_{1B-B2-14}$**

Final structure in terms of initial Cartesian coordinates:

N 2.361125 0.706054 0.250646
N 0.148175 1.258816 1.198611
C 2.918159 1.823634 0.923626
C 0.735890 2.315419 1.939061
C 2.087909 2.562596 1.752735
H 2.528804 3.391798 2.297333
C 3.208133 -0.164605 -0.515400
C 3.224097 -0.067967 -1.913866
C 4.005928 -1.099449 0.170280
C 4.071519 -0.924740 -2.623352
C 4.849115 -1.921370 -0.574801
C 4.884142 -1.834718 -1.963617
H 4.094399 -0.872180 -3.708860
H 5.479838 -2.646487 -0.069814
H 5.542444 -2.485670 -2.531242
C -1.272612 1.066326 1.217656
C -1.817826 -0.017874 1.920743
C -2.086598 1.978996 0.516384
C -3.208694 -0.165694 1.924277
C -3.469403 1.822235 0.590304
C -4.029136 0.755209 1.288790
H -3.652694 -1.007232 2.451011
H -4.120978 2.519298 0.074099
H -5.108187 0.630853 1.311554
C 2.331883 0.905018 -2.663208
H 1.881099 1.580464 -1.928376
C 3.866125 -1.261000 1.674815
H 3.619931 -0.282013 2.101696
C -0.957757 -1.010601 2.684122
H 0.082106 -0.670994 2.630264
C -1.446429 3.044954 -0.359675
H -0.554723 3.420173 0.156032
C -0.118060 3.052927 2.918269
H -0.958828 3.574684 2.441060
H -0.558003 2.370518 3.658629
H 0.484491 3.792478 3.450220
C 4.352718 2.156574 0.673170
H 4.547605 2.321300 -0.395216
H 4.619172 3.066821 1.214858
H 5.034505 1.356240 0.992088
C -1.034314 -2.411460 2.066026
H -2.062096 -2.793526 2.086882
H -0.690982 -2.411085 1.027884
H -0.407734 -3.110441 2.631345
C -1.344323 -1.053321 4.167543
H -1.291332 -0.058880 4.621498
H -2.363794 -1.430909 4.302318
H -0.668339 -1.718098 4.715527
C -0.981401 2.413853 -1.682855
H -0.393815 1.504993 -1.515378
H -1.846383 2.135146 -2.292785
H -0.366866 3.117831 -2.256024
C -2.359285 4.238291 -0.640072
H -3.208311 3.952226 -1.270437
H -2.750591 4.672944 0.285697
H -1.803624 5.014761 -1.174431
C 2.691251 -2.207041 1.968222
H 1.780471 -1.886703 1.451930
H 2.925531 -3.218428 1.616696
H 2.485060 -2.254598 3.043624
C 5.138732 -1.758015 2.361444
H 5.366634 -2.794538 2.090442
H 6.004013 -1.139974 2.100520
H 5.011167 -1.728170 3.447881
C 3.121232 1.769093 -3.652138
H 3.549235 1.165008 -4.459196
H 2.461550 2.512915 -4.111044
H 3.940798 2.296775 -3.154478
C 1.202237 0.152900 -3.381764
H 1.614320 -0.511641 -4.150116
H 0.625545 -0.460211 -2.681817
H 0.522039 0.858616 -3.872554
C -2.258142 -0.827344 -1.780748
C -3.529458 -0.311282 -1.748966
C -1.942643 -2.086603 -1.330630

C -2.968758 -2.851565 -0.780138
C -4.536792 -1.102130 -1.200805
C -4.268147 -2.370904 -0.714375
F 0.423750 -0.589669 -0.287752
F -2.676584 -4.064396 -0.306551
F -0.715515 -2.596862 -1.401935
H -5.056142 -2.972055 -0.277732
F -3.830185 0.911611 -2.192153
F -5.770171 -0.596763 -1.119863
B 0.972030 0.441312 0.378314

[3]1B

Final structure in terms of initial Cartesian coordinates:

N -1.043925 -0.648543 0.595115
N 1.043925 0.648543 0.595115
C -1.012656 -0.694796 2.017250
C 1.012656 0.694796 2.017250
C 0.000000 0.000000 2.660936
H 0.000000 0.000000 3.746857
C -2.151139 -1.242273 -0.100158
C -1.987992 -2.484188 -0.731279
C -3.382820 -0.560256 -0.118811
C -3.086320 -3.032608 -1.400502
C -4.455947 -1.153129 -0.781799
C -4.310388 -2.380792 -1.421437
H -2.978626 -3.988812 -1.905779
H -5.417029 -0.648881 -0.807727
H -5.155027 -2.826773 -1.938128
C 2.151139 1.242273 -0.100158
C 1.987992 2.484188 -0.731279
C 3.382820 0.560256 -0.118811
C 3.086320 3.032608 -1.400502
C 4.455947 1.153129 -0.781799
C 4.310388 2.380792 -1.421437
H 2.978626 3.988812 -1.905779
H 5.417029 0.648881 -0.807727
H 5.155027 2.826773 -1.938128
C -0.657555 -3.215866 -0.737746
H -0.003205 -2.735336 -0.002131
C -3.501294 0.824850 0.496772
H -2.822426 0.874808 1.355686
C 0.657555 3.215866 -0.737746
H 0.003205 2.735336 -0.002131
C 3.501294 -0.824850 0.496772
H 2.822426 -0.874808 1.355686
C 2.039705 1.515606 2.727890
H 1.835521 1.509509 3.801138
H 3.062788 1.146329 2.572456
H 2.031378 2.558092 2.382054
C -2.039705 -1.515606 2.727890
H -1.835521 -1.509509 3.801138
H -3.062788 -1.146329 2.572456
H -2.031378 -2.558092 2.382054
C 0.000000 3.088420 -2.117936
H 0.629554 3.554009 -2.885103
H -0.138211 2.035447 -2.387897
H -0.977816 3.582527 -2.131436
C 0.798710 4.687193 -0.335381
H 1.299878 4.788550 0.632235
H 1.373191 5.255423 -1.074634
H -0.190164 5.151239 -0.260793
C 3.032857 -1.873270 -0.521835
H 2.024537 -1.645903 -0.883157
H 3.702915 -1.887136 -1.389220
H 3.023273 -2.874773 -0.076253
C 4.907594 -1.153927 0.999682
H 5.618328 -1.263343 0.173531
H 5.286042 -0.378349 1.673699
H 4.894825 -2.103705 1.543092
C -3.032857 1.873270 -0.521835
H -2.024537 1.645903 -0.883157
H -3.702915 1.887136 -1.389220
H -3.023273 2.874773 -0.076253
C -4.907594 1.153927 0.999682
H -5.618328 1.263343 0.173531
H -5.286042 0.378349 1.673699
H -4.894825 2.103705 1.543092
C -0.798710 -4.687193 -0.335381
H -1.373191 -5.255423 -1.074634
H 0.190164 -5.151239 -0.260793
H -1.299878 -4.788550 0.632235
C 0.000000 -3.088420 -2.117936
H -0.629554 -3.554009 -2.885103
H 0.138211 -2.035447 -2.387897
H 0.977816 -3.582527 -2.131436

B 0.000000 0.000000 -0.104773

[3]IM$_{1B_A1-14}$

Final structure in terms of initial Cartesian coordinates:

B 0.053592 -0.717522 0.459586
N -1.173140 -1.246921 0.924655
N 1.283122 -1.116438 1.025947
C -1.160056 -2.196042 1.989086
C 1.294921 -2.064896 2.084157
C 0.071608 -2.553874 2.518086
H 0.078621 -3.279832 3.325587
C -2.422249 -0.895880 0.307445
C -2.742111 -1.440189 -0.949848
C -3.299890 -0.031952 0.984713
C -3.984457 -1.121046 -1.501648
C -4.536019 0.246924 0.400525
C -4.879657 -0.298218 -0.829347
H -4.260558 -1.523809 -2.470479
H -5.233806 0.906294 0.907240
H -5.844499 -0.071654 -1.273438
C 2.519505 -0.631444 0.478353
C 3.138095 0.479737 1.074548
C 3.064367 -1.261583 -0.651149
C 4.286525 0.996099 0.474308
C 4.226183 -0.722482 -1.208843
C 4.825702 0.404182 -0.662278
H 4.767022 1.872694 0.899741
H 4.657485 -1.184327 -2.093497
H 5.719103 0.820182 -1.118191
C -1.775048 -2.366775 -1.673426
H -0.767035 -1.961268 -1.531954
C -2.869338 0.655816 2.270043
H -2.186150 -0.011243 2.807422
C 2.549780 1.137221 2.311557
H 1.871168 0.419570 2.785373
C 2.407903 -2.473722 -1.289375
H 1.587286 -2.799078 -0.639677
C 2.613258 -2.487531 2.645141
H 2.459012 -3.227918 3.433499
H 3.261870 -2.932537 1.878570
H 3.174033 -1.644787 3.073227
C -2.456365 -2.783925 2.448913
H -2.262155 -3.543827 3.209623
H -3.133790 -2.035578 2.881773
H -3.005726 -3.255958 1.623820
C 1.719866 2.358811 1.905130
H 2.351060 3.097234 1.396051
H 0.923548 2.065214 1.214155
H 1.261116 2.834416 2.779078
C 3.616850 1.520207 3.340533
H 4.255435 0.667222 3.591767
H 4.261035 2.326925 2.975143
H 3.140518 1.875193 4.259878
C 1.813331 -2.107007 -2.654395
H 1.109478 -1.274506 -2.568405
H 2.603561 -1.803860 -3.351406
H 1.290035 -2.967128 -3.088057
C 3.385409 -3.646453 -1.421785
H 4.207576 -3.406556 -2.104896
H 3.820361 -3.915397 -0.454085
H 2.868967 -4.525354 -1.821436
C -2.082506 1.922884 1.911655
H -1.208815 1.667623 1.303366
H -2.708072 2.614077 1.334549
H -1.733400 2.439169 2.812960
C -4.028784 0.990111 3.209755
H -4.671972 1.774250 2.796229
H -4.650896 0.111962 3.412642
H -3.638477 1.361350 4.162299
C -1.792793 -3.777370 -1.070564
H -2.801688 -4.203393 -1.122267
H -1.117567 -4.434886 -1.630150
H -1.469112 -3.774767 -0.025446
C -2.019663 -2.431934 -3.181335
H -2.949238 -2.962092 -3.417915
H -2.070511 -1.431565 -3.621103
H -1.202593 -2.977345 -3.663602
C -0.412082 1.526047 -1.889751
C 0.918975 1.919105 -1.803931
C -1.423603 2.351889 -1.422576
C -1.088485 3.591868 -0.890904
C 1.224389 3.160375 -1.265658
C 0.228709 4.010764 -0.806730
F -0.717749 0.345135 -2.419549
F -2.073865 4.368969 -0.438482

H 0.477631 4.971886 -0.374275
F -2.689140 1.952213 -1.469108
F 1.878612 1.107510 -2.234553
F 2.506437 3.512421 -1.163672

[3]TS$_{1B_A1-14}$

Final structure in terms of initial Cartesian coordinates:
B -0.101173 -0.372120 0.434574
N -1.374277 -0.766514 0.927988
N 1.079078 -0.751718 1.123923
C -1.458962 -1.556716 2.116943
C 0.980694 -1.522739 2.322444
C -0.279682 -1.886203 2.760005
H -0.346969 -2.480127 3.666144
C -2.608564 -0.519838 0.233163
C -2.933099 -1.315645 -0.879123
C -3.476089 0.468630 0.727658
C -4.146022 -1.062266 -1.523871
C -4.680205 0.676514 0.055402
C -5.008915 -0.074753 -1.066344
H -4.423989 -1.647687 -2.394626
H -5.369564 1.438182 0.405886
H -5.947300 0.105435 -1.582363
C 2.401488 -0.563156 0.590722
C 3.139178 0.562943 0.993348
C 2.940972 -1.544828 -0.253053
C 4.384232 0.771390 0.399800
C 4.206484 -1.312415 -0.796850
C 4.906373 -0.150441 -0.502949
H 4.966404 1.649449 0.662179
H 4.640256 -2.043714 -1.473787
H 5.877465 0.026884 -0.955239
C -2.033333 -2.454763 -1.340212
H -0.998475 -2.178786 -1.120268
C -3.094511 1.329397 1.923669
H -2.453011 0.734032 2.581866
C 2.607566 1.482417 2.084490
H 1.956368 0.877382 2.725992
C 2.189796 -2.824768 -0.585658
H 1.321721 -2.895158 0.080707
C 2.238134 -1.945099 3.013642
H 1.981195 -2.485754 3.927516
H 2.855923 -2.604924 2.390658
H 2.874966 -1.094190 3.288552
C -2.801222 -1.996122 2.617586
H -2.658679 -2.687265 3.452004
H -3.421923 -1.163537 2.974294
H -3.386967 -2.508324 1.845615
C 1.751166 2.611863 1.512123
H 2.356761 3.259334 0.867936
H 0.927537 2.213852 0.910918
H 1.324637 3.223342 2.314835
C 3.721652 2.052694 2.965823
H 4.383196 1.264144 3.338486
H 4.334056 2.779395 2.421171
H 3.286050 2.572937 3.824562
C 1.676693 -2.813415 -2.031040
H 1.002878 -1.973360 -2.213926
H 2.512693 -2.729205 -2.735175
H 1.140878 -3.744743 -2.248564
C 3.056798 -4.065775 -0.342129
H 3.900257 -4.108207 -1.039634
H 3.461864 -4.083885 0.674415
H 2.462256 -4.973066 -0.489830
C -2.267444 2.541093 1.478068
H -1.311481 2.218951 1.054802
H -2.796833 3.124657 0.717905
H -2.050816 3.192456 2.331924
C -4.304655 1.782648 2.744536
H -4.901195 2.526454 2.205778
H -4.959143 0.942368 2.999485
H -3.967376 2.250199 3.674602
C -2.329084 -3.744165 -0.562454
H -3.386529 -4.018871 -0.651970
H -1.727663 -4.569412 -0.960403
H -2.084997 -3.635253 0.498417
C -2.119413 -2.711649 -2.846040
H -3.072539 -3.175831 -3.123176
H -2.006087 -1.785427 -3.415985
H -1.324090 -3.400622 -3.147760
C -0.029527 0.852358 -1.558296
C 1.324419 1.191838 -1.849912
C -0.913933 1.964690 -1.471738
C -0.436778 3.255187 -1.364542
C 1.781240 2.486904 -1.745382

C 0.913777 3.548566 -1.503508
F -0.519620 -0.224833 -2.239857
F -1.320402 4.240321 -1.174228
H 1.281116 4.562622 -1.413464
F -2.225786 1.751512 -1.472865
F 2.149555 0.223236 -2.235453
F 3.085590 2.723900 -1.911443

^{3}IM$_{1B_A2-14}$

Final structure in terms of initial Cartesian coordinates:
B -0.105947 -0.214734 0.300135
N -1.390467 -0.598792 0.833750
N 1.054764 -0.558344 1.079877
C -1.488285 -1.333765 2.055697
C 0.928021 -1.214324 2.342767
C -0.332020 -1.582491 2.764696
H -0.418419 -2.110266 3.709135
C -2.664094 -0.381592 0.195452
C -3.075898 -1.247913 -0.828472
C -3.500555 0.631750 0.701775
C -4.327808 -1.020152 -1.408150
C -4.738565 0.818351 0.089839
C -5.144851 0.010200 -0.967236
H -4.668905 -1.666835 -2.211444
H -5.401342 1.601948 0.442976
H -6.111625 0.174242 -1.433881
C 2.412900 -0.490116 0.604286
C 3.194781 0.631411 0.926090
C 2.946957 -1.603625 -0.064281
C 4.476332 0.707608 0.378469
C 4.244207 -1.495644 -0.569303
C 4.985586 -0.335191 -0.388322
H 5.095674 1.577375 0.575970
H 4.676227 -2.326720 -1.119739
H 5.982747 -0.257188 -0.810915
C -2.250401 -2.450337 -1.265970
H -1.213423 -2.278908 -0.969423
C -3.075095 1.512880 1.872578
H -2.420528 0.918943 2.518921
C 2.686472 1.681880 1.905419
H 1.958620 1.187443 2.558832
C 2.155609 -2.895275 -0.229436
H 1.327171 -2.876761 0.488359
C 2.158290 -1.567336 3.121302
H 1.855928 -2.017549 4.069730
H 2.798918 -2.286958 2.594664
H 2.788725 -0.698246 3.346633
C -2.828650 -1.766608 2.574346
H -2.673183 -2.456366 3.407594
H -3.438322 -0.931223 2.944287
H -3.434433 -2.274497 1.818123
C 1.949232 2.827672 1.213920
H 2.602876 3.334295 0.494510
H 1.073055 2.457722 0.675658
H 1.606431 3.564089 1.948766
C 3.805717 2.229939 2.795651
H 4.383155 1.423905 3.259715
H 4.499261 2.860599 2.229490
H 3.377454 2.849429 3.589743
C 1.549597 -3.043629 -1.632048
H 0.833843 -2.250103 -1.858281
H 2.334931 -3.009397 -2.396128
H 1.033256 -4.007178 -1.713051
C 3.015223 -4.122744 0.098615
H 3.790158 -4.283935 -0.658169
H 3.508906 -4.023767 1.070531
H 2.389276 -5.020126 0.121035
C -2.257684 2.735397 1.432238
H -1.279984 2.439840 1.040633
H -2.777075 3.306077 0.655963
H -2.081428 3.393281 2.290094
C -4.266234 1.966894 2.723363
H -4.868703 2.719383 2.203290
H -4.921529 1.129346 2.984589
H -3.906001 2.424140 3.649905
C -2.718673 -3.727847 -0.555407
H -3.790634 -3.892776 -0.713923
H -2.179418 -4.596246 -0.950231
H -2.530615 -3.680935 0.520832
C -2.268337 -2.659918 -2.783156
H -3.236798 -3.042006 -3.124819
H -2.053824 -1.731653 -3.316914
H -1.511550 -3.401189 -3.061145
C 0.002604 0.555154 -1.207300
C 1.374165 0.805902 -1.775010

C -0.752912 1.851016 -1.336755
C -0.194025 3.065203 -1.600447
C 1.909871 2.031036 -2.040641
C 1.164364 3.210874 -1.917620
F -0.607442 -0.342603 -2.117834
F -0.987561 4.142579 -1.610578
H 1.600099 4.181063 -2.115768
F -2.076799 1.783488 -1.273430
F 2.038729 -0.269301 -2.182085
F 3.169257 2.101768 -2.484288

^{3}TS$_{1B_A2-14}$

Final structure in terms of initial Cartesian coordinates:
B -0.014600 0.425152 -0.343839
N 1.265317 1.112054 -0.466700
N -1.145666 1.239879 0.050005
C 1.264227 2.522932 -0.616686
C -1.010982 2.641950 0.228193
C 0.158156 3.241009 -0.206698
H 0.209424 4.325885 -0.222964
C 2.576901 0.570121 -0.229465
C 3.307945 -0.055934 -1.248560
C 3.139747 0.776776 1.050996
C 4.588965 -0.534874 -0.945683
C 4.429945 0.316888 1.293991
C 5.150850 -0.350452 0.307088
H 5.159469 -1.041456 -1.720008
H 4.876669 0.469439 2.273186
H 6.151747 -0.716533 0.516157
C -2.433987 0.684528 0.343405
C -2.599684 -0.092556 1.503964
C -3.504666 0.933182 -0.538727
C -3.855475 -0.659863 1.743989
C -4.739683 0.353642 -0.249402
C -4.915805 -0.445459 0.874527
H -4.007045 -1.266666 2.632490
H -5.577653 0.520843 -0.919491
H -5.883887 -0.894416 1.075700
C 2.784469 -0.203061 -2.666520
H 1.850221 0.357425 -2.736961
C 2.363685 1.465530 2.166498
H 1.319617 1.564216 1.849412
C -1.483498 -0.291637 2.521962
H -0.522383 -0.090508 2.040106
C -3.333263 1.766533 -1.803726
H -2.479999 2.436071 -1.660161
C -2.130545 3.422256 0.845837
H -1.798447 4.450560 1.006778
H -3.036504 3.453636 0.226737
H -2.438326 3.009757 1.814295
C 2.451216 3.170770 -1.262673
H 2.295425 4.251461 -1.312445
H 3.391611 2.981823 -0.730924
H 2.587703 2.801319 -2.288525
C -1.434494 -1.723397 3.067347
H -2.247567 -1.913363 3.776332
H -1.517504 -2.462995 2.263050
H -0.491497 -1.891940 3.598385
C -1.610850 0.715578 3.671720
H -1.487516 1.740276 3.308558
H -2.592788 0.633649 4.151162
H -0.841266 0.529209 4.428786
C -3.010336 0.891771 -3.023783
H -2.039790 0.404106 -2.918857
H -3.775677 0.118285 -3.158279
H -2.991094 1.511697 -3.926662
C -4.561488 2.637120 -2.095211
H -5.410432 2.036103 -2.438772
H -4.882995 3.201154 -1.213268
H -4.327901 3.349503 -2.892267
C 2.371987 0.659391 3.471173
H 1.976480 -0.349139 3.319441
H 3.381413 0.567925 3.886193
H 1.754909 1.164778 4.222187
C 2.905895 2.879170 2.414593
H 3.955699 2.840356 2.728601
H 2.839412 3.492161 1.511199
H 2.333434 3.375172 3.205792
C 3.771268 0.369602 -3.692014
H 4.694786 -0.218165 -3.734495
H 3.325101 0.353749 -4.691975
H 4.045066 1.402143 -3.453686
C 2.477888 -1.669634 -2.992426
H 3.391192 -2.275074 -2.951102
H 1.753712 -2.086226 -2.287836

H 2.056632 -1.758219 -3.999460
C -0.176458 -1.208657 -0.213377
C -1.272951 -1.954399 -0.759817
C 0.760259 -2.018349 0.476988
C 0.655933 -3.386852 0.566305
C -1.374079 -3.348856 -0.660662
C -0.406898 -4.084655 -0.024680
F -0.048441 -0.456721 -2.056789
F 1.543216 -4.058951 1.289835
H -0.473797 -5.163310 0.052814
F 1.701819 -1.459306 1.233064
F -2.235116 -1.389672 -1.445308
F -2.418743 -3.940314 -1.232353

[3]15B

Final structure in terms of initial Cartesian coordinates:

B 0.167636 -0.306437 -0.679047
N -1.198761 -1.091518 -0.669139
N 1.246503 -1.192129 0.071593
C -1.130830 -2.419269 -1.030191
C 1.152383 -2.551945 0.024124
C 0.018934 -3.116372 -0.597609
H 0.019547 -4.196880 -0.727057
C -2.516658 -0.574541 -0.445692
C -3.078731 0.369145 -1.320212
C -3.250147 -1.093818 0.648308
C -4.406985 0.754743 -1.109816
C -4.573439 -0.689634 0.799785
C -5.157851 0.221486 -0.075421
H -4.857172 1.477583 -1.785202
H -5.154810 -1.078107 1.631497
H -6.191787 0.522871 0.062562
C 2.527619 -0.598595 0.348280
C 2.715803 0.009071 1.604423
C 3.554856 -0.621185 -0.618759
C 3.946824 0.604257 1.879529
C 4.761836 0.004632 -0.297416
C 4.963449 0.611756 0.934540
H 4.104591 1.071971 2.847624
H 5.559848 0.018105 -1.034969
H 5.913072 1.088990 1.157040
C -2.325657 0.961773 -2.497777
H -1.302562 0.582783 -2.492606
C -2.628292 -2.014064 1.693699
H -1.540970 -1.980727 1.567844
C 1.634620 -0.000401 2.668683
H 0.691469 -0.264849 2.187458
C 3.418948 -1.270516 -1.989824
H 2.469288 -1.811598 -2.028472
C 2.273071 -3.420451 0.512593
H 1.933233 -4.454167 0.609373
H 3.131022 -3.407036 -0.169465
H 2.642242 -3.085146 1.486819
C -2.216454 -3.070145 -1.834004
H -2.035219 -4.146128 -1.911033
H -3.214402 -2.921370 -1.408166
H -2.237122 -2.661840 -2.853833
C 1.468783 1.375591 3.328122
H 2.241443 1.551111 4.085256
H 1.532681 2.180031 2.589022
H 0.494671 1.442452 3.824993
C 1.912363 -1.069203 3.733985
H 1.908097 -2.075665 3.304213
H 2.888040 -0.903948 4.204656
H 1.145513 -1.032798 4.515341
C 3.392923 -0.212840 -3.100668
H 2.562925 0.478244 -2.963570
H 4.329026 0.357315 -3.109704
H 3.287390 -0.699377 -4.076428
C 4.558434 -2.263443 -2.264191
H 5.514391 -1.743190 -2.386551
H 4.686189 -2.996766 -1.462210
H 4.361997 -2.805264 -3.195023
C -2.938738 -1.539300 3.120900
H -2.714658 -0.478525 3.248953
H -3.991199 -1.702889 3.376687
H -2.339063 -2.111416 3.836654
C -3.086282 -3.470028 1.535993
H -4.179399 -3.536984 1.582536
H -2.759520 -3.903654 0.587701
H -2.678146 -4.083064 2.346904
C -2.959745 0.534786 -3.827940
H -3.986888 0.906860 -3.913579
H -2.384961 0.937187 -4.668404
H -2.988756 -0.555091 -3.923324

C -2.270812 2.491927 -2.396369
H -3.257793 2.935121 -2.571572
H -1.930342 2.813049 -1.407041
H -1.583802 2.898032 -3.146187
C 0.054039 1.201442 -0.072325
C 0.716837 2.274901 -0.668815
C -0.728394 1.543415 1.026556
C -0.894728 2.845387 1.476582
C 0.572612 3.586520 -0.225248
C -0.246901 3.894485 0.847416
F 0.590906 -0.271353 -2.017802
F -1.645124 3.062970 2.561281
H -0.354569 4.913773 1.197420
F -1.314918 0.582550 1.764886
F 1.538509 2.096340 -1.708403
F 1.240843 4.556306 -0.856133

^{3}IM$_{1B_B-14}$

Final structure in terms of initial Cartesian coordinates:
N -1.994409 -0.115872 0.920885
N 0.354408 0.069818 1.631946
C -2.399018 -0.179481 2.283793
C -0.056231 0.020190 2.998168
C -1.412635 -0.111489 3.254359
H -1.727044 -0.138063 4.293234
C -3.013915 -0.033357 -0.089236
C -3.624150 -1.214397 -0.544252
C -3.403020 1.227841 -0.565056
C -4.627446 -1.110382 -1.507466
C -4.407589 1.285809 -1.534525
C -5.017499 0.129822 -2.001645
H -5.107592 -2.010009 -1.881133
H -4.715213 2.251904 -1.926429
H -5.798241 0.194035 -2.753709
C 1.753663 0.223435 1.351619
C 2.243594 1.485779 0.961898
C 2.603325 -0.881021 1.543512
C 3.625690 1.629023 0.814534
C 3.976749 -0.689847 1.373827
C 4.484634 0.556515 1.027076
H 4.037760 2.587638 0.518183
H 4.659651 -1.519389 1.528142
H 5.556753 0.693142 0.915348
C -3.140722 -2.569911 -0.059848
H -2.671237 -2.429988 0.920059
C -2.737189 2.506576 -0.087880
H -2.092334 2.258272 0.762248
C 1.293430 2.651073 0.701333
H 0.491426 2.265632 0.056428
C 2.027156 -2.242132 1.910610
H 1.192302 -2.077094 2.600737
C 0.964329 0.225413 4.073037
H 1.725989 -0.564780 4.105399
H 1.505693 1.171642 3.939014
H 0.464723 0.253085 5.044480
C -3.857372 -0.277567 2.598548
H -4.314081 -1.192433 2.196687
H -3.996944 -0.278356 3.682094
H -4.425357 0.562725 2.178450
C 1.949397 3.813673 -0.045995
H 2.661713 4.347010 0.594465
H 2.473732 3.486347 -0.945862
H 1.179273 4.531341 -0.346511
C 0.646381 3.186969 1.986725
H 0.046659 2.433885 2.503947
H 1.418507 3.549226 2.675918
H -0.009215 4.031884 1.744714
C 1.448014 -2.937474 0.673281
H 0.681665 -2.318717 0.195717
H 2.231839 -3.142860 -0.063061
H 0.986121 -3.891258 0.951211
C 3.030700 -3.157306 2.613781
H 3.821101 -3.488490 1.931216
H 3.502935 -2.661139 3.468306
H 2.520680 -4.054372 2.977365
C -1.856445 3.078810 -1.204466
H -1.162220 2.323347 -1.582501
H -2.473340 3.414730 -2.046503
H -1.277855 3.938327 -0.845163
C -3.752132 3.546579 0.396274
H -4.399694 3.884888 -0.419647
H -4.390129 3.142668 1.188512
H -3.230917 4.425488 0.790097
C -4.263848 -3.595905 0.102719
H -4.697634 -3.877134 -0.862757

H -3.870679 -4.509674 0.559179
H -5.069942 -3.213666 0.737738
C -2.063926 -3.083086 -1.023319
H -2.502075 -3.287164 -2.007464
H -1.278362 -2.336565 -1.163854
H -1.605985 -4.006060 -0.650948
C 0.859874 -0.246231 -2.405615
C 1.480449 -1.485334 -2.314929
C 1.623453 0.913941 -2.368385
C 3.001365 0.821615 -2.231939
C 2.859777 -1.548847 -2.168512
C 3.635425 -0.404077 -2.123090
F -0.458094 -0.168116 -2.550796
F 3.717932 1.947184 -2.208351
F 1.028913 2.103600 -2.460317
H 4.708641 -0.462975 -1.995203
F 0.757004 -2.601963 -2.356186
F 3.431045 -2.751018 -2.070332
B -0.621577 0.011372 0.608451

^{3}TS$_{1B_B-14}$

Final structure in terms of initial Cartesian coordinates:

N 2.199980 0.560872 0.522951
N -0.057102 1.195134 1.299863
C 2.772498 1.381689 1.541367
C 0.523205 1.978594 2.339213
C 1.907358 2.039794 2.401458
H 2.347025 2.658322 3.177904
C 3.062422 -0.238542 -0.306144
C 3.374356 0.188014 -1.603706
C 3.586789 -1.429678 0.231465
C 4.224127 -0.615608 -2.371081
C 4.439597 -2.191297 -0.564642
C 4.756722 -1.788997 -1.858888
H 4.472360 -0.310773 -3.384460
H 4.858149 -3.114026 -0.175414
H 5.418359 -2.397068 -2.468495
C -1.481456 1.195836 1.085113
C -2.272846 0.190327 1.664509
C -2.032759 2.201799 0.273498
C -3.620748 0.133284 1.300837
C -3.385724 2.109674 -0.055797
C -4.168888 1.068383 0.430252
H -4.251730 -0.650328 1.713483
H -3.830090 2.853039 -0.711122
H -5.213176 0.995966 0.140542
C 2.810467 1.465119 -2.201380
H 2.268114 1.999077 -1.413805
C 3.159510 -1.899878 1.612272
H 2.979559 -1.013905 2.232372
C -1.735628 -0.757398 2.726396
H -0.683366 -0.506390 2.903080
C -1.176517 3.358124 -0.222593
H -0.308523 3.432723 0.442203
C -0.388997 2.694091 3.279735
H -1.062557 3.387635 2.758076
H -1.027431 1.999372 3.840690
H 0.200114 3.268079 3.998679
C 4.259623 1.515519 1.588918
H 4.662172 1.880077 0.634577
H 4.539989 2.222964 2.372690
H 4.762639 0.560630 1.793219
C -1.809095 -2.229449 2.311632
H -2.832639 -2.510630 2.036293
H -1.157693 -2.448031 1.465014
H -1.500730 -2.870413 3.144850
C -2.500916 -0.558179 4.043695
H -2.525481 0.492031 4.348777
H -3.537899 -0.897900 3.946048
H -2.033910 -1.140195 4.844781
C -0.645690 3.107265 -1.637957
H -0.140024 2.139821 -1.703677
H -1.466538 3.096815 -2.362574
H 0.061383 3.894468 -1.924297
C -1.917214 4.697112 -0.155995
H -2.733993 4.743567 -0.884036
H -2.340179 4.873681 0.838258
H -1.228369 5.515579 -0.387571
C 1.832819 -2.662645 1.497558
H 1.076828 -2.070139 0.973356
H 1.971945 -3.586041 0.923866
H 1.445421 -2.925771 2.488257
C 4.212018 -2.751907 2.322746
H 4.342716 -3.723852 1.835315
H 5.185185 -2.250530 2.350309

H 3.897515 -2.947005 3.352575
C 3.920501 2.392826 -2.708464
H 4.466593 1.941025 -3.543511
H 3.491950 3.335332 -3.064437
H 4.643197 2.620349 -1.918595
C 1.820369 1.137514 -3.326210
H 2.325526 0.605837 -4.140522
H 1.006273 0.504093 -2.960520
H 1.387729 2.055852 -3.737911
C -1.343801 -1.244577 -1.444221
C -2.362477 -0.479838 -1.973455
C -1.582349 -2.531666 -1.011073
C -2.867123 -3.052634 -1.092628
C -3.642413 -1.013683 -2.051534
C -3.913408 -2.297050 -1.601779
F -0.108316 -0.526060 -0.907697
F -3.086040 -4.299915 -0.665658
F -0.592996 -3.287822 -0.514863
H -4.915682 -2.703371 -1.654817
F -2.133621 0.751868 -2.440554
F -4.625047 -0.258583 -2.552309
B 0.801459 0.522195 0.414374

^{3}IM'$_{1B\_B-14}$

Final structure in terms of initial Cartesian coordinates:

N 2.360968 0.705394 0.253022
N 0.147579 1.255309 1.201589
C 2.917674 1.820961 0.929608
C 0.734913 2.309786 1.945393
C 2.086999 2.557573 1.760373
H 2.527726 3.385027 2.307766
C 3.208368 -0.162755 -0.515473
C 3.225052 -0.061323 -1.913602
C 4.005796 -1.099917 0.167420
C 4.072768 -0.915748 -2.625594
C 4.849257 -1.919411 -0.580052
C 4.884949 -1.828058 -1.968549
H 4.096151 -0.859516 -3.710902
H 5.479714 -2.646264 -0.077246
H 5.543403 -2.477186 -2.538076
C -1.273166 1.062365 1.220088
C -1.818022 -0.024031 1.920059
C -2.087449 1.977107 0.521859
C -3.208880 -0.171956 1.923665
C -3.470210 1.819925 0.595698
C -4.029615 0.750775 1.291192
H -3.652647 -1.015085 2.448038
H -4.122086 2.518480 0.081916
H -5.108647 0.626250 1.313957
C 2.333070 0.914203 -2.659937
H 1.882106 1.587178 -1.922943
C 3.865552 -1.266462 1.671376
H 3.619137 -0.288910 2.101423
C -0.957589 -1.018842 2.680319
H 0.082242 -0.679061 2.626988
C -1.447497 3.045881 -0.350901
H -0.556481 3.420399 0.166537
C -0.119397 3.044122 2.926669
H -0.960190 3.567229 2.450956
H -0.559360 2.359334 3.664808
H 0.482941 3.782072 3.461085
C 4.352459 2.154405 0.681087
H 4.548160 2.321976 -0.386700
H 4.618683 3.063133 1.225432
H 5.033901 1.353105 0.998335
C -1.034237 -2.418067 2.058553
H -2.062088 -2.800033 2.078035
H -0.690473 -2.415130 1.020551
H -0.408022 -3.118622 2.622317
C -1.343493 -1.065578 4.163824
H -1.290261 -0.072384 4.620467
H -2.362914 -1.443486 4.298034
H -0.667234 -1.731843 4.709666
C -0.980977 2.418845 -1.675454
H -0.392857 1.510003 -1.510001
H -1.845227 2.141111 -2.286896
H -0.366508 3.124900 -2.246135
C -2.361079 4.239246 -0.628850
H -3.209661 3.953927 -1.260153
H -2.752986 4.671595 0.297741
H -1.805770 5.017285 -1.161278
C 2.690846 -2.213788 1.961305
H 1.779994 -1.891876 1.446144
H 2.925439 -3.223823 1.606101
H 2.484657 -2.265343 3.036524

C 5.138049 -1.765585 2.356679
H 5.366062 -2.801216 2.082368
H 6.003351 -1.146663 2.097910
H 5.010262 -1.739244 3.443174
C 3.122533 1.781663 -3.645819
H 3.551161 1.180274 -4.454551
H 2.462686 2.526571 -4.102727
H 3.941623 2.308222 -3.146197
C 1.203589 0.164453 -3.381268
H 1.615901 -0.496960 -4.152202
H 0.627101 -0.451588 -2.683702
H 0.523098 0.871776 -3.869311
C -2.257990 -0.822016 -1.783335
C -3.529286 -0.306060 -1.749635
C -1.942375 -2.082664 -1.337234
C -2.968370 -2.849390 -0.789036
C -4.536524 -1.098660 -1.203808
C -4.267765 -2.368981 -0.721487
F 0.424189 -0.589459 -0.289272
F -2.676093 -4.063801 -0.319542
F -0.715216 -2.592640 -1.410184
H -5.055647 -2.971598 -0.286667
F -3.830045 0.918241 -2.188961
F -5.769893 -0.593584 -1.121064
B 0.971910 0.440028 0.379510

IM_{1Ga-14}

Final structure in terms of initial Cartesian coordinates:

Ga -0.576726 0.403671 -0.729402
N -2.310555 0.647356 0.390855
N 0.319275 1.613974 0.696547
C -2.582641 1.587794 1.288297
C -0.268940 2.530057 1.459398
C -1.649532 2.554297 1.701194
H -2.013246 3.328858 2.363710
C -3.298708 -0.338804 0.078252
C -3.127071 -1.648110 0.576224
C -4.366812 -0.021489 -0.781703
C -4.091077 -2.606183 0.260292
C -5.302322 -1.015353 -1.074166
C -5.179013 -2.293355 -0.546439
H -3.986170 -3.617340 0.638744
H -6.134992 -0.787762 -1.733741
H -5.918231 -3.053502 -0.779715
C 1.739086 1.455742 0.741833
C 2.570938 2.124721 -0.172396
C 2.269345 0.531127 1.670844
C 3.947469 1.887906 -0.115653
C 3.651829 0.342177 1.702117
C 4.489480 1.020287 0.822292
H 4.604525 2.394367 -0.818706
H 4.080882 -0.363122 2.406746
H 5.560516 0.848149 0.850672
C -1.924811 -2.006471 1.440728
H -1.047382 -1.514021 0.998923
C -4.462162 1.335848 -1.460418
H -3.847748 2.046836 -0.899569
C 2.016311 3.083038 -1.213155
H 0.945863 3.211286 -1.016780
C 1.355988 -0.238557 2.616618
H 0.417835 -0.423034 2.078822
C 0.574529 3.606984 2.106236
H 1.441373 3.183915 2.620488
H 0.958796 4.276902 1.328167
H -0.011432 4.195789 2.813767
C -3.953288 1.637000 1.928178
H -4.215474 0.655412 2.335615
H -3.982612 2.376132 2.729687
H -4.721656 1.887826 1.189488
C 2.168260 2.509119 -2.627847
H 3.227791 2.346864 -2.860732
H 1.642746 1.553483 -2.730223
H 1.763607 3.205343 -3.370138
C 2.682943 4.461967 -1.127967
H 2.623643 4.878497 -0.117619
H 3.742482 4.405506 -1.400199
H 2.201283 5.161308 -1.818964
C 1.932021 -1.590898 3.042873
H 2.290156 -2.177366 2.194808
H 2.763330 -1.467056 3.746182
H 1.159999 -2.172552 3.557954
C 1.002646 0.573377 3.872496
H 1.915588 0.915383 4.374121
H 0.382275 1.442960 3.643567
H 0.442644 -0.052177 4.576572

C -3.873668 1.240602 -2.874886
H -2.837425 0.886114 -2.846270
H -4.451668 0.533923 -3.480881
H -3.895283 2.216883 -3.371203
C -5.890212 1.887792 -1.506341
H -6.528777 1.297267 -2.171423
H -6.355089 1.892907 -0.514625
H -5.883754 2.913358 -1.888343
C -2.079269 -1.481596 2.874060
H -2.998803 -1.874194 3.323604
H -1.234671 -1.808001 3.492136
H -2.114107 -0.388043 2.909110
C -1.603152 -3.501296 1.454271
H -2.367727 -4.076196 1.988185
H -1.510703 -3.897361 0.438555
H -0.650278 -3.663338 1.966921
C 2.083989 -2.699930 -0.566552
C 3.379230 -2.222316 -0.413091
C 1.318189 -2.327808 -1.662650
C 1.859308 -1.456474 -2.599738
C 3.901843 -1.363188 -1.369550
C 3.148929 -0.967671 -2.463143
F 1.566205 -3.517958 0.348707
F 1.109829 -1.079141 -3.631763
F 0.078391 -2.792239 -1.785756
H 3.558952 -0.284235 -3.195940
F 4.105640 -2.594210 0.640024
F 5.157987 -0.941018 -1.228418

$TS_{1Ga\text{-}14}$

Final structure in terms of initial Cartesian coordinates:

Ga -0.026782 0.031603 0.542450
N -1.407990 -1.370651 0.634405
N 1.419617 -1.290051 0.794459
C -1.248915 -2.561504 1.208251
C 1.251900 -2.492560 1.347251
C -0.001375 -3.060369 1.611588
H -0.001753 -4.025689 2.100000
C -2.688336 -0.990464 0.104368
C -2.912749 -1.171494 -1.273673
C -3.637899 -0.377544 0.937221
C -4.144249 -0.767596 -1.790492
C -4.848619 0.021988 0.369664
C -5.106703 -0.178699 -0.979249
H -4.349211 -0.900145 -2.848016
H -5.594766 0.509316 0.991139
H -6.053709 0.139463 -1.404077
C 2.732431 -0.876057 0.377860
C 3.457430 0.036088 1.162549
C 3.223023 -1.333082 -0.860142
C 4.689230 0.486282 0.682986
C 4.459276 -0.855732 -1.295356
C 5.189547 0.048777 -0.534960
H 5.258629 1.201882 1.269970
H 4.849756 -1.187887 -2.253580
H 6.144899 0.416770 -0.895475
C -1.862928 -1.801066 -2.178962
H -0.881425 -1.620026 -1.729508
C -3.352106 -0.074766 2.398700
H -2.463223 -0.640324 2.700666
C 2.934998 0.570226 2.485833
H 2.033491 0.006422 2.751905
C 2.435406 -2.280388 -1.752875
H 1.568014 -2.652328 -1.195464
C 2.475270 -3.306860 1.688837
H 2.979945 -3.632601 0.773534
H 3.194184 -2.700891 2.248687
H 2.205983 -4.187266 2.273162
C -2.465741 -3.416377 1.470354
H -3.099990 -3.487659 0.583599
H -2.176786 -4.417289 1.792656
H -3.072610 -2.953882 2.257667
C 2.551439 2.050798 2.356410
H 3.419363 2.648670 2.056981
H 1.772895 2.200305 1.601416
H 2.181740 2.437321 3.312044
C 3.948090 0.371483 3.619428
H 4.245384 -0.678090 3.710969
H 4.854219 0.962741 3.451232
H 3.516872 0.692041 4.573086
C 1.909441 -1.532731 -2.984839
H 1.299890 -0.670696 -2.699921
H 2.743771 -1.168334 -3.595019
H 1.303725 -2.202659 -3.605940

C 3.267139 -3.494562 -2.184582
H 4.103536 -3.195184 -2.824694
H 3.683326 -4.033378 -1.327099
H 2.646666 -4.190871 -2.757700
C -3.037765 1.417819 2.572703
H -2.183243 1.719232 1.958730
H -3.893443 2.026333 2.260197
H -2.814752 1.646058 3.620352
C -4.502253 -0.493350 3.320867
H -5.397032 0.112074 3.143138
H -4.776707 -1.543253 3.174098
H -4.216037 -0.354329 4.368185
C -2.046504 -3.321059 -2.272662
H -3.046529 -3.568219 -2.647221
H -1.309334 -3.750817 -2.960101
H -1.916056 -3.804695 -1.299338
C -1.840594 -1.173330 -3.574245
H -2.734073 -1.432623 -4.152376
H -1.765669 -0.084990 -3.509189
H -0.974970 -1.543549 -4.132317
C -0.105038 1.869275 -0.799801
C 1.106763 2.567957 -0.971845
C -1.270755 2.662005 -0.770656
C -1.200259 4.038859 -0.730372
C 1.155868 3.946100 -0.922007
C 0.007166 4.722204 -0.827218
F -0.222631 0.648651 -1.642029
F -2.343130 4.726151 -0.605166
F -2.452653 2.041684 -0.588136
H 0.049133 5.802214 -0.826151
F 2.249012 1.852673 -0.982644
F 2.356622 4.536444 -0.981343

15Ga

Final structure in terms of initial Cartesian coordinates:

Ga 0.102088 -0.086408 -0.394368
N -1.118779 -1.118664 0.706896
N 1.644950 -0.245596 0.758862
C -0.855812 -1.402700 1.978800
C 1.530560 -0.602923 2.039760
C 0.348075 -1.071480 2.624217
H 0.400806 -1.308759 3.678157
C -2.319900 -1.616370 0.085850
C -2.232788 -2.793369 -0.683397
C -3.537597 -0.923583 0.230532
C -3.401260 -3.286973 -1.266999
C -4.673628 -1.453465 -0.384982
C -4.614913 -2.629195 -1.119586
H -3.358173 -4.204215 -1.847236
H -5.622084 -0.932626 -0.284225
H -5.512045 -3.028835 -1.582109
C 2.955556 -0.049095 0.194670
C 3.452833 1.252258 0.013228
C 3.700352 -1.179210 -0.196415
C 4.707234 1.406470 -0.582310
C 4.946229 -0.974737 -0.789900
C 5.449941 0.305930 -0.985559
H 5.103169 2.407537 -0.731924
H 5.529645 -1.833767 -1.109482
H 6.420983 0.443834 -1.450885
C -0.931885 -3.560800 -0.871648
H -0.107873 -2.955249 -0.481438
C -3.668216 0.381525 1.000427
H -2.702506 0.610370 1.463830
C 2.670796 2.482395 0.435492
H 1.740900 2.145889 0.902038
C 3.173177 -2.595466 -0.027591
H 2.297484 -2.566914 0.630408
C 2.764203 -0.565233 2.907108
H 2.502351 -0.695889 3.957381
H 3.454675 -1.364373 2.615894
H 3.297764 0.380442 2.777293
C -1.894907 -2.129001 2.795911
H -1.478213 -2.460922 3.746900
H -2.738708 -1.461889 2.996951
H -2.290141 -2.990684 2.251564
C 2.318625 3.344753 -0.784077
H 3.213956 3.835877 -1.181987
H 1.883671 2.742762 -1.587087
H 1.597959 4.123816 -0.508669
C 3.432393 3.312845 1.475047
H 3.669648 2.724490 2.366928
H 4.374117 3.693042 1.064665
H 2.830789 4.173573 1.784827
C 2.714133 -3.150440 -1.381830

H 1.940645 -2.515102 -1.823644
H 3.559080 -3.201879 -2.078365
H 2.313676 -4.163706 -1.258729
C 4.205248 -3.529306 0.615106
H 5.057941 -3.700119 -0.050288
H 4.592778 -3.127156 1.557006
H 3.750704 -4.503876 0.819553
C -4.029525 1.531971 0.049422
H -3.301774 1.627190 -0.760686
H -5.014199 1.362401 -0.400177
H -4.068187 2.480663 0.595939
C -4.729989 0.296967 2.106302
H -5.730603 0.181260 1.676436
H -4.566241 -0.546540 2.783165
H -4.729523 1.216827 2.699370
C -0.958146 -4.872763 -0.077736
H -1.765518 -5.525690 -0.428136
H -0.011346 -5.410097 -0.199994
H -1.110892 -4.693479 0.991805
C -0.645208 -3.820044 -2.354051
H -1.434001 -4.421944 -2.817557
H -0.554958 -2.874245 -2.893149
H 0.294403 -4.370198 -2.463896
C -0.583460 1.773977 -0.546569
C -0.875591 2.434577 0.635638
C -0.844108 2.466952 -1.718259
C -1.411736 3.713281 0.673503
F -0.629416 1.810544 1.808607
C -1.384467 3.749903 -1.709587
F -0.555916 1.921321 -2.904745
C -1.676117 4.383898 -0.511696
F -1.687195 4.287551 1.848908
F -1.617167 4.372760 -2.867696
F 0.369771 -0.862587 -1.903485
H -2.099382 5.381063 -0.502604

18

Final structure in terms of initial Cartesian coordinates:
C 0.000000 1.388994 0.000000
C 1.202904 0.694497 0.000000
C 1.202904 -0.694497 0.000000
C 0.000000 -1.388994 0.000000
C -1.202904 -0.694497 0.000000
C -1.202904 0.694497 0.000000
F 0.000000 2.716761 0.000000
F 2.352784 1.358380 0.000000
F 2.352784 -1.358380 0.000000
F 0.000000 -2.716761 0.000000
F -2.352784 -1.358380 0.000000
F -2.352784 1.358380 0.000000

IM_{1-18}

Final structure in terms of initial Cartesian coordinates:
Al 0.617497 0.383222 -0.631310
N -0.178490 1.742037 0.574385
N 2.367075 0.745397 0.242152
C 0.429884 2.755299 1.188588
C 2.721209 1.801256 0.974317
C 1.820246 2.812628 1.339431
H 2.225575 3.667602 1.864954
C -1.592140 1.560041 0.734854
C -2.026111 0.744124 1.801888
C -2.498976 2.088938 -0.198779
C -3.394959 0.507135 1.937674
C -3.858158 1.810600 -0.030746
C -4.307611 1.039754 1.032903
H -3.753409 -0.117997 2.749842
H -4.574214 2.203921 -0.748087
H -5.367031 0.832206 1.144933
C 3.346620 -0.269491 -0.033369
C 4.308089 -0.066172 -1.039760
C 3.267238 -1.489974 0.668864
C 5.239018 -1.078685 -1.279900
C 4.222728 -2.469717 0.396408
C 5.210744 -2.264372 -0.559397
H 5.990770 -0.940417 -2.051922
H 4.188171 -3.415078 0.927764
H 5.945152 -3.039481 -0.756091
C -1.031994 0.157365 2.796292
H -0.070903 0.054589 2.279430
C -2.044449 2.939170 -1.372802
H -0.970098 3.123956 -1.259549

C 4.299234 1.175116 -1.918126
H 3.700833 1.946976 -1.423168
C 2.173119 -1.733996 1.700200
H 1.245959 -1.301866 1.298797
C 4.148656 1.939560 1.451054
H 4.480033 1.018987 1.941115
H 4.823495 2.108773 0.605604
H 4.244843 2.772445 2.148570
C -0.391599 3.903307 1.726772
H -1.266133 3.553254 2.279830
H 0.208538 4.550643 2.368091
H -0.762301 4.496882 0.882178
C 3.615319 0.851971 -3.253891
H 4.174503 0.076306 -3.789179
H 2.596924 0.481120 -3.095085
H 3.568137 1.742595 -3.889761
C 5.698710 1.751369 -2.158146
H 6.240009 1.915993 -1.220366
H 6.305588 1.086014 -2.780754
H 5.624747 2.708737 -2.683476
C 1.878516 -3.215559 1.935995
H 1.690521 -3.735426 0.991695
H 2.701411 -3.716994 2.457001
H 0.984433 -3.314923 2.559165
C 2.478865 -1.026685 3.026724
H 3.443552 -1.363858 3.423430
H 2.512473 0.061476 2.911462
H 1.707685 -1.257958 3.770686
C -2.257943 2.201860 -2.700071
H -1.707466 1.255206 -2.723244
H -3.319296 1.979485 -2.853662
H -1.915180 2.817826 -3.538436
C -2.758679 4.296526 -1.395226
H -3.828537 4.175645 -1.596575
H -2.659326 4.821953 -0.439967
H -2.343943 4.931540 -2.184644
C -0.801263 1.100184 3.985748
H -1.749089 1.318424 4.491177
H -0.125972 0.633763 4.711660
H -0.350235 2.046764 3.675836
C -1.439163 -1.226495 3.306068
H -2.318596 -1.176290 3.957359
H -1.661358 -1.915501 2.488297
H -0.624875 -1.654811 3.900550
C -1.821073 -2.764508 -0.190830
C -1.173944 -2.498999 -1.389027
C -3.104187 -2.285045 0.036083
C -3.748799 -1.546201 -0.946034
C -1.810581 -1.740940 -2.362168
C -3.097037 -1.268882 -2.140425
F -1.196325 -3.467073 0.755073
F -4.998443 -1.132500 -0.760000
F -3.720509 -2.549040 1.185909
F 0.060571 -2.944693 -1.582759
F -1.183556 -1.437798 -3.491214
F -3.715088 -0.554375 -3.077141

TS$_{1\text{-}18}$

Final structure in terms of initial Cartesian coordinates:

Al -0.081135 -0.236545 0.775012
N -1.579239 -1.441050 0.440439
N 1.185055 -1.690653 0.542943
C -1.556713 -2.757126 0.657996
C 0.922560 -2.972963 0.804786
C -0.379624 -3.469788 0.928870
H -0.485353 -4.522294 1.155642
C -2.833718 -0.823702 0.091209
C -3.121857 -0.603594 -1.267882
C -3.715492 -0.408657 1.104887
C -4.344445 -0.015824 -1.595729
C -4.915984 0.195334 0.727745
C -5.239515 0.377841 -0.609864
H -4.591408 0.152555 -2.640021
H -5.606482 0.532398 1.496210
H -6.181594 0.842927 -0.883811
C 2.517518 -1.331149 0.136770
C 3.455584 -0.884473 1.080818
C 2.824063 -1.392177 -1.236914
C 4.723075 -0.515631 0.625776
C 4.108514 -1.027103 -1.640404
C 5.053776 -0.591600 -0.719746
H 5.459961 -0.155305 1.338763
H 4.371412 -1.076142 -2.693608
H 6.045375 -0.301847 -1.053590
C -2.137968 -0.970154 -2.367144

H -1.157917 -1.104090 -1.903715
C -3.379462 -0.542664 2.581515
H -2.498723 -1.187759 2.678844
C 3.122887 -0.748707 2.556871
H 2.148336 -1.219142 2.733132
C 1.811141 -1.864267 -2.270819
H 0.824658 -1.874247 -1.795722
C 2.069631 -3.934710 0.997837
H 2.765837 -3.906970 0.156516
H 2.639834 -3.638114 1.885999
H 1.706347 -4.953776 1.134518
C -2.851894 -3.532593 0.646279
H -3.433574 -3.316070 -0.253453
H -2.662571 -4.604669 0.707089
H -3.470324 -3.234383 1.500087
C 3.007153 0.732128 2.943404
H 3.956918 1.249685 2.767362
H 2.233388 1.235644 2.355218
H 2.753182 0.833264 4.003988
C 4.150668 -1.455694 3.448219
H 4.276751 -2.506668 3.168063
H 5.132159 -0.974789 3.380998
H 3.835056 -1.414319 4.495509
C 1.736580 -0.926728 -3.480071
H 1.539413 0.102686 -3.171722
H 2.665127 -0.943523 -4.060645
H 0.930248 -1.250443 -4.147970
C 2.113805 -3.295133 -2.735464
H 3.117788 -3.355851 -3.170765
H 2.057859 -4.012174 -1.911129
H 1.393247 -3.607750 -3.499075
C -3.020700 0.831042 3.164864
H -2.193651 1.291187 2.614224
H -3.881830 1.506400 3.107562
H -2.728163 0.738763 4.216278
C -4.516286 -1.180716 3.388517
H -5.395252 -0.528714 3.421895
H -4.831493 -2.140035 2.965330
H -4.195361 -1.350297 4.421192
C -2.508313 -2.295799 -3.041399
H -3.508072 -2.240175 -3.487153
H -1.792844 -2.531262 -3.837585
H -2.499216 -3.125394 -2.326770
C -2.000538 0.151510 -3.401626
H -2.900877 0.244002 -4.018607
H -1.820236 1.111688 -2.911167
H -1.162319 -0.057627 -4.073381
C 0.310877 1.831132 -0.432644
C 1.573342 2.441306 -0.375630
C -0.806115 2.679790 -0.476657
C -0.684555 4.032769 -0.227155
C 1.698089 3.796137 -0.127934
C 0.572576 4.611062 -0.080217
F 0.239216 0.637533 -1.244086
F -1.773376 4.798177 -0.158516
F -2.025486 2.127034 -0.531851
F 2.667910 1.672303 -0.369979
F 2.908748 4.333501 0.020935
F 0.694816 5.926459 0.103749

19

Final structure in terms of initial Cartesian coordinates:
Al 0.237104 0.185290 -0.379897
N 1.695272 -0.234320 0.745634
N -0.517392 1.551239 0.695242
C 1.745489 0.134822 2.029118
C -0.180137 1.738636 1.970493
C 0.824324 1.004887 2.621670
H 0.967677 1.215747 3.672847
C 2.835957 -0.899932 0.165510
C 3.915077 -0.113710 -0.283190
C 2.845388 -2.298731 0.030817
C 4.993387 -0.757585 -0.890465
C 3.948924 -2.897712 -0.581842
C 5.013947 -2.138376 -1.044544
H 5.829210 -0.165071 -1.253473
H 3.967569 -3.978311 -0.698737
H 5.860337 -2.620799 -1.523620
C -1.466815 2.439446 0.068245
C -2.851722 2.234971 0.226051
C -0.966820 3.497921 -0.715864
C -3.724353 3.136429 -0.387740
C -1.883005 4.377121 -1.296426
C -3.250732 4.205712 -1.133767
H -4.796147 2.994944 -0.275537

H -1.517061 5.212373 -1.886570
H -3.946485 4.900897 -1.593578
C 3.933738 1.401555 -0.148354
H 3.084242 1.708955 0.472193
C 1.701887 -3.170215 0.518113
H 0.960037 -2.520613 0.991327
C -3.442666 1.071587 1.008218
H -2.624161 0.516342 1.478742
C 0.521958 3.741609 -0.920715
H 1.072531 2.863052 -0.571742
C -0.896914 2.792148 2.775621
H -0.395720 2.958355 3.729255
H -0.955890 3.735181 2.225696
H -1.924415 2.471467 2.972370
C 2.886781 -0.358668 2.880657
H 2.729447 -0.098766 3.927595
H 2.995591 -1.443039 2.786661
H 3.829959 0.084580 2.544119
C -4.193166 0.118587 0.066179
H -5.046801 0.628746 -0.393580
H -3.546319 -0.243986 -0.737238
H -4.577298 -0.743255 0.623125
C -4.404620 1.540574 2.109499
H -3.954139 2.282384 2.775160
H -5.302421 1.993607 1.675835
H -4.725569 0.687153 2.714993
C 0.859413 3.925570 -2.403423
H 0.559303 3.043369 -2.973972
H 0.364235 4.805852 -2.826227
H 1.937055 4.066253 -2.529556
C 0.992920 4.943642 -0.093046
H 0.467644 5.856248 -0.396680
H 0.812348 4.790679 0.976344
H 2.066858 5.107324 -0.235126
C 1.036376 -3.895827 -0.659545
H 0.830791 -3.209884 -1.485717
H 1.687109 -4.691643 -1.039522
H 0.091303 -4.354064 -0.345186
C 2.164096 -4.184569 1.571407
H 2.900482 -4.879956 1.154375
H 2.620876 -3.693804 2.436521
H 1.312149 -4.773886 1.925873
C 5.212595 1.901557 0.534785
H 6.095679 1.702954 -0.081547
H 5.156107 2.983713 0.690329
H 5.372491 1.425227 1.507505
C 3.767190 2.062566 -1.521740
H 4.593720 1.783183 -2.185119
H 2.827175 1.756851 -1.989339
H 3.773381 3.153855 -1.416749
C -1.049672 -1.308808 -0.542562
C -1.563462 -1.830628 0.634745
C -1.539830 -1.875755 -1.710480
C -2.512393 -2.840105 0.689397
F -1.125056 -1.333770 1.813276
C -2.492066 -2.889726 -1.717618
F -1.081850 -1.473639 -2.900863
C -2.982033 -3.367233 -0.508121
F -2.992811 -3.289386 1.848921
F -2.935794 -3.413638 -2.858590
F -3.899037 -4.329290 -0.495035
F 0.748477 0.774170 -1.856155

20

Final structure in terms of initial Cartesian coordinates:

C -1.213801 0.379112 0.000000
C 0.000000 1.043728 0.000000
C 1.199932 0.339178 0.000000
C 1.196787 -1.044620 0.000000
C -0.013429 -1.735351 0.000000
C -1.194140 -1.009572 0.000000
F 0.035590 2.376748 0.000000
F 2.348838 1.022095 0.000000
H 2.146666 -1.567521 0.000000
H -0.049255 -2.818013 0.000000
H -2.146034 0.930581 0.000000
F -2.362591 -1.663276 0.000000

IM_{1-20}

Final structure in terms of initial Cartesian coordinates:

Al 0.193775 0.492403 -0.175854
N 1.745730 -0.617577 -0.803303

N -0.968192 -0.823021 -1.126228
C 1.808501 -1.388046 -1.881445
C -0.652110 -1.571777 -2.190743
C 0.667725 -1.789628 -2.605604
H 0.830740 -2.416132 -3.472227
C 2.961329 -0.302475 -0.113888
C 3.113373 -0.712982 1.230264
C 3.934907 0.480657 -0.754483
C 4.310615 -0.382317 1.870292
C 5.117022 0.786758 -0.058284
C 5.313606 0.341992 1.231958
H 4.477222 -0.691380 2.894266
H 5.873471 1.406099 -0.526613
H 6.240478 0.556646 1.754803
C -2.336847 -0.865932 -0.669335
C -3.317590 0.026778 -1.167324
C -2.672277 -1.819143 0.310463
C -4.602089 -0.036938 -0.631091
C -3.985204 -1.843333 0.806780
C -4.940162 -0.960670 0.346853
H -5.376990 0.621354 -1.014395
H -4.269859 -2.577723 1.550872
H -5.957486 -1.008727 0.722581
C 2.014906 -1.487305 1.981662
H 1.043076 -0.963945 1.829723
C 3.726687 1.036688 -2.168956
H 2.909516 0.489906 -2.650218
C -3.008844 1.067085 -2.230715
H -2.040164 0.827101 -2.686894
C -1.662303 -2.827117 0.834477
H -0.684536 -2.571997 0.429099
C -1.781863 -2.213278 -2.959192
H -1.457399 -2.910980 -3.727495
H -2.492952 -2.703474 -2.298017
H -2.333641 -1.393903 -3.428734
C 3.151192 -1.930658 -2.308515
H 3.069663 -2.527819 -3.218005
H 3.849528 -1.101966 -2.465311
H 3.578535 -2.553662 -1.511861
C -2.942569 2.439484 -1.550189
H -3.868142 2.586938 -0.983144
H -2.096330 2.479580 -0.859076
H -2.836286 3.249483 -2.280443
C -4.053046 1.077574 -3.347653
H -4.246939 0.066107 -3.720482
H -5.007388 1.509349 -3.031557
H -3.690035 1.682398 -4.185749
C -1.576914 -2.763652 2.361347
H -1.181698 -1.789774 2.667290
H -2.558397 -2.886473 2.823589
H -0.928386 -3.543576 2.779965
C -2.026067 -4.256686 0.390599
H -2.888612 -4.610779 0.961152
H -2.290739 -4.318036 -0.667789
H -1.183028 -4.930389 0.581112
C 3.306846 2.508398 -2.091598
H 2.437418 2.626117 -1.437337
H 4.126858 3.108432 -1.684787
H 3.054776 2.898172 -3.084314
C 4.944741 0.900168 -3.088713
H 5.752693 1.568075 -2.780077
H 5.358522 -0.109742 -3.123504
H 4.663065 1.181223 -4.108847
C 1.913922 -2.927953 1.457463
H 2.860936 -3.456097 1.621390
H 1.124328 -3.490950 1.972375
H 1.686120 -2.939789 0.392914
C 2.223104 -1.506179 3.488301
H 2.957349 -2.257875 3.800804
H 2.545215 -0.526746 3.847671
H 1.270796 -1.751616 3.961558
C -0.823343 1.252561 2.771911
C -2.084808 1.071498 2.246076
C -0.030466 2.350229 2.477842
C -0.545005 3.291505 1.609291
C -2.572613 2.010475 1.331059
C -1.810090 3.136813 1.051684
F -0.294379 0.322267 3.590717
H -2.672365 0.202972 2.505086
H -3.534863 1.868163 0.846216
H 0.968444 2.452786 2.885441
F 0.145002 4.391249 1.310929
F -2.245062 4.084266 0.212600

TS_{1-20}

Final structure in terms of initial

Cartesian coordinates:
Al 0.039362 -0.096895 0.770548
N -1.242881 -1.501749 0.409098
N 1.528239 -1.293062 0.464193
C -1.009712 -2.795050 0.619729
C 1.482821 -2.606418 0.680760
C 0.279316 -3.311027 0.831475
H 0.354275 -4.370225 1.038587
C -2.563889 -1.040404 0.077159
C -2.906268 -0.886476 -1.281084
C -3.451788 -0.663007 1.102448
C -4.174990 -0.390929 -1.588773
C -4.701925 -0.156172 0.742300
C -5.069359 -0.027439 -0.590280
H -4.462269 -0.277955 -2.630339
H -5.395368 0.147902 1.522086
H -6.047330 0.365341 -0.851060
C 2.770416 -0.649317 0.134559
C 3.526117 -0.028076 1.146922
C 3.152882 -0.563467 -1.219747
C 4.683087 0.663704 0.781319
C 4.326313 0.126127 -1.531424
C 5.088389 0.738102 -0.544436
H 5.271695 1.155322 1.551443
H 4.640891 0.194747 -2.569462
H 5.991663 1.278916 -0.809532
C -1.952707 -1.247896 -2.409298
H -0.958947 -1.394422 -1.976116
C -3.082123 -0.740274 2.574990
H -2.124098 -1.265067 2.664152
C 3.107973 -0.044937 2.607939
H 2.241981 -0.708809 2.710380
C 2.339100 -1.185773 -2.344492
H 1.401930 -1.559258 -1.919142
C 2.777624 -3.371279 0.789057
H 2.596349 -4.432806 0.959180
H 3.382665 -3.248278 -0.112819
H 3.369300 -2.969618 1.619468
C -2.174810 -3.751444 0.668337
H -1.833526 -4.783716 0.750511
H -2.802174 -3.514074 1.535225
H -2.809053 -3.647541 -0.215888
C 2.680782 1.360199 3.054241
H 3.532048 2.049789 3.017704
H 1.891215 1.761115 2.408711
H 2.306023 1.338091 4.082839
C 4.217774 -0.585368 3.517623
H 4.550784 -1.579865 3.203544
H 5.091889 0.074351 3.513978
H 3.859647 -0.652937 4.549748
C 1.987754 -0.153259 -3.422553
H 1.464176 0.704960 -2.994906
H 2.887237 0.207131 -3.933554
H 1.343230 -0.611871 -4.180202
C 3.080394 -2.369540 -2.981042
H 4.036817 -2.044213 -3.405650
H 3.287770 -3.163827 -2.257986
H 2.482502 -2.800223 -3.791002
C -2.900013 0.672098 3.148294
H -2.144969 1.233821 2.587344
H -3.842850 1.228595 3.100799
H -2.584963 0.624018 4.196025
C -4.120571 -1.518546 3.391890
H -5.078910 -0.989439 3.422249
H -4.304373 -2.514044 2.974983
H -3.776761 -1.636148 4.424378
C -2.370961 -2.559142 -3.086664
H -3.377324 -2.471383 -3.511613
H -1.680373 -2.805390 -3.900366
H -2.373805 -3.397323 -2.383015
C -1.849644 -0.123255 -3.445992
H -2.789093 0.002016 -3.995028
H -1.595548 0.829327 -2.973360
H -1.073069 -0.361024 -4.179825
C -0.034110 1.880812 -0.290759
C 1.146774 2.662474 -0.348375
C -1.289101 2.535375 -0.388160
C -1.330089 3.911672 -0.335529
C 1.062160 4.046365 -0.296139
C -0.169990 4.680590 -0.277824
F 0.040850 0.658206 -1.129920
H 2.117096 2.173321 -0.362305
H 1.960065 4.655541 -0.280188
H -2.221862 1.979322 -0.410958
F -2.512773 4.540329 -0.349802
F -0.265805 6.019798 -0.213391

21

Final structure in terms of initial Cartesian coordinates:

Al	-0.103138	-0.655176	-0.660159
N	-1.436990	-0.948428	0.667494
N	1.337459	-1.281487	0.400596
C	-1.323142	-1.929572	1.553517
C	1.138299	-2.284283	1.266550
C	-0.122921	-2.641974	1.749604
H	-0.162694	-3.475175	2.439095
C	-2.650163	-0.185746	0.546968
C	-3.740087	-0.706248	-0.169387
C	-2.655718	1.124574	1.061434
C	-4.862641	0.108080	-0.332667
C	-3.799734	1.898679	0.869033
C	-4.897693	1.396382	0.180429
H	-5.715091	-0.271025	-0.890385
H	-3.832108	2.912110	1.255653
H	-5.777910	2.015920	0.036310
C	2.666597	-0.768562	0.213271
C	3.209202	0.094252	1.188468
C	3.369362	-1.069931	-0.966876
C	4.466783	0.650786	0.950813
C	4.619903	-0.478717	-1.163019
C	5.169077	0.374530	-0.216327
H	4.896398	1.326565	1.685880
H	5.168293	-0.692148	-2.076717
H	6.141021	0.827221	-0.386707
C	-3.714586	-2.075597	-0.829441
H	-2.815389	-2.607803	-0.502239
C	-1.467994	1.668664	1.841342
H	-0.562571	1.188816	1.452468
C	2.459373	0.486897	2.455038
H	1.522209	-0.079499	2.501911
C	2.800291	-1.988208	-2.034645
H	1.879210	-2.437271	-1.654254
C	2.333536	-3.046198	1.780559
H	2.019264	-3.928350	2.339070
H	2.971701	-3.351450	0.945061
H	2.945021	-2.417360	2.433983
C	-2.513398	-2.327162	2.390237
H	-2.196947	-2.885583	3.272002
H	-3.092661	-1.453391	2.697577
H	-3.177904	-2.964803	1.796551
C	2.099635	1.978223	2.420731
H	3.004346	2.595455	2.385235
H	1.493805	2.223200	1.543667
H	1.537853	2.257536	3.319226
C	3.267525	0.180563	3.722845
H	3.569995	-0.869370	3.778936
H	4.176898	0.789447	3.764674
H	2.673647	0.411628	4.612920
C	2.436619	-1.187147	-3.291609
H	1.776005	-0.346859	-3.055146
H	3.339106	-0.780683	-3.763514
H	1.923790	-1.827045	-4.015785
C	3.764534	-3.128989	-2.378326
H	4.690542	-2.751440	-2.825279
H	4.033984	-3.708130	-1.489034
H	3.300426	-3.807367	-3.101040
C	-1.286683	3.177955	1.675550
H	-1.248422	3.455200	0.616570
H	-2.097258	3.739363	2.153117
H	-0.352916	3.495641	2.149151
C	-1.582807	1.289062	3.323466
H	-2.512312	1.682400	3.750528
H	-1.578159	0.202229	3.458905
H	-0.743452	1.702314	3.893797
C	-4.938964	-2.917613	-0.447769
H	-5.856542	-2.494420	-0.870208
H	-4.835715	-3.933254	-0.842561
H	-5.075133	-2.979837	0.637097
C	-3.622795	-1.929049	-2.354221
H	-4.491441	-1.382081	-2.738580
H	-2.717014	-1.393389	-2.642533
H	-3.603639	-2.916773	-2.826964
C	0.055389	1.222283	-1.249491
C	1.166240	2.067806	-1.095567
C	-1.053934	1.800717	-1.860783
C	-1.131212	3.121094	-2.279938
C	1.110636	3.391785	-1.508822
C	-0.020850	3.939312	-2.094923
F	-0.396637	-1.745346	-1.901332
H	2.098396	1.714488	-0.660981
F	-2.140469	1.020539	-2.073607
F	2.192113	4.173154	-1.339050
H	-2.042442	3.487415	-2.740006

H -0.017816 4.979264 -2.402388

IM$_{1-20meta}$

Final structure in terms of initial Cartesian coordinates:

Al 0.357421 0.092679 -0.822017
N 2.034533 -0.836924 -0.248104
N -0.637073 -1.545628 -0.252854
C 2.238641 -2.144201 -0.125295
C -0.161485 -2.793819 -0.267163
C 1.206059 -3.086466 -0.264476
H 1.487518 -4.131497 -0.251956
C 3.126507 0.067879 -0.023819
C 3.145116 0.807271 1.176933
C 4.098333 0.268207 -1.020411
C 4.199794 1.695225 1.389292
C 5.132396 1.170915 -0.763280
C 5.195332 1.868580 0.434629
H 4.237736 2.272955 2.307022
H 5.893165 1.339122 -1.520734
H 6.007005 2.566370 0.616862
C -2.022568 -1.364590 0.076141
C -2.999155 -1.262503 -0.929894
C -2.367366 -1.257407 1.442532
C -4.333652 -1.095995 -0.546511
C -3.717423 -1.135615 1.776116
C -4.698576 -1.059988 0.791590
H -5.097844 -0.998431 -1.312773
H -4.006897 -1.075105 2.820907
H -5.742542 -0.951237 1.069799
C 2.044775 0.634955 2.214201
H 1.099616 0.519185 1.669511
C 4.002691 -0.394182 -2.385919
H 3.285754 -1.219319 -2.323249
C -2.656093 -1.317131 -2.409393
H -1.601409 -1.600710 -2.503253
C -1.303671 -1.266859 2.533876
H -0.395331 -0.835596 2.098638
C -1.128727 -3.955614 -0.289827
H -0.597059 -4.907663 -0.257894
H -1.833387 -3.909971 0.544734
H -1.723038 -3.912072 -1.209709
C 3.622712 -2.668344 0.179306
H 3.593782 -3.737925 0.390640
H 4.295792 -2.494764 -0.666445
H 4.051304 -2.142627 1.037989
C -2.832868 0.064013 -3.054416
H -3.857769 0.428006 -2.918784
H -2.151359 0.794248 -2.606037
H -2.624016 0.012621 -4.128614
C -3.491961 -2.366021 -3.153733
H -3.419337 -3.353048 -2.685420
H -4.550634 -2.086567 -3.178601
H -3.150403 -2.451629 -4.190150
C -1.696732 -0.413289 3.744286
H -2.045580 0.576906 3.442442
H -2.481354 -0.895769 4.337825
H -0.829926 -0.286866 4.401908
C -0.957471 -2.686704 3.007476
H -1.865131 -3.221756 3.311172
H -0.450104 -3.269756 2.234759
H -0.287836 -2.639066 3.873411
C 3.454395 0.612665 -3.407096
H 2.478424 0.997323 -3.093247
H 4.137774 1.463921 -3.504597
H 3.345823 0.144954 -4.391575
C 5.340575 -0.966227 -2.867679
H 6.060696 -0.169981 -3.083274
H 5.794202 -1.631581 -2.125633
H 5.195081 -1.532225 -3.793189
C 2.256619 -0.632649 3.051613
H 3.236552 -0.606797 3.542619
H 1.489683 -0.708467 3.831473
H 2.198912 -1.539564 2.440518
C 1.856480 1.853159 3.117611
H 2.700458 1.990231 3.802803
H 1.727601 2.764637 2.527071
H 0.957072 1.716206 3.726398
C -1.674096 2.452585 0.674941
C -2.926368 2.032770 0.269907
C -0.994204 3.458824 0.000916
C -1.567037 4.063688 -1.102511
C -3.480584 2.651999 -0.841618
C -2.828936 3.657960 -1.536361
F -1.068624 1.856904 1.714938
H -3.441610 1.230287 0.785443

F 0.230514 3.799184 0.416216
F -4.687070 2.240317 -1.264612
H -1.011539 4.835368 -1.622945
H -3.300621 4.104484 -2.403576

TS1-20meta

Final structure in terms of initial Cartesian coordinates:
Al 0.136851 0.032244 -0.828170
N 1.707847 -1.014814 -0.314953
N -1.023130 -1.494917 -0.470273
C 1.789721 -2.341363 -0.410242
C -0.661392 -2.769454 -0.598963
C 0.680001 -3.168916 -0.646515
H 0.874365 -4.226037 -0.769960
C 2.896873 -0.263886 -0.006360
C 3.139676 0.113051 1.326818
C 3.765760 0.123087 -1.044154
C 4.301893 0.834100 1.609226
C 4.903783 0.860374 -0.714044
C 5.182233 1.204001 0.602194
H 4.513911 1.117062 2.636978
H 5.581053 1.174091 -1.504118
H 6.077308 1.770223 0.841058
C -2.376329 -1.165637 -0.125986
C -3.289436 -0.776074 -1.121223
C -2.729903 -1.150459 1.240054
C -4.570306 -0.380263 -0.726063
C -4.026494 -0.764076 1.582699
C -4.943003 -0.377132 0.611457
H -5.284543 -0.064210 -1.482193
H -4.319130 -0.754375 2.629325
H -5.942955 -0.066771 0.898325
C 2.186280 -0.228796 2.460674
H 1.271293 -0.640185 2.024952
C 3.483277 -0.187516 -2.505804
H 2.647499 -0.894652 -2.555516
C -2.925318 -0.739181 -2.595410
H -1.916471 -1.152154 -2.708751
C -1.751956 -1.544084 2.337362
H -0.755900 -1.623726 1.890552
C -1.726865 -3.830986 -0.726464
H -1.295936 -4.831175 -0.667746
H -2.499734 -3.722398 0.037278
H -2.223919 -3.717895 -1.697512
C 3.136983 -3.008760 -0.275641
H 3.035377 -4.094304 -0.276583
H 3.786201 -2.712385 -1.106559
H 3.639201 -2.692069 0.642511
C -2.902429 0.706859 -3.107338
H -3.899169 1.157810 -3.038683
H -2.208794 1.320792 -2.523114
H -2.588463 0.735517 -4.156049
C -3.880503 -1.595448 -3.435738
H -3.918405 -2.629155 -3.077147
H -4.899819 -1.195729 -3.406193
H -3.559219 -1.605039 -4.482153
C -1.682752 -0.489788 3.447664
H -1.472829 0.501548 3.038252
H -2.620129 -0.440365 4.012630
H -0.888149 -0.750051 4.156056
C -2.108103 -2.911797 2.935473
H -3.118589 -2.896614 3.359608
H -2.067915 -3.708761 2.187125
H -1.408393 -3.169398 3.737669
C 3.059623 1.092061 -3.240397
H 2.186715 1.548455 -2.763088
H 3.875811 1.823647 -3.229843
H 2.810360 0.872356 -4.284048
C 4.683834 -0.829790 -3.211359
H 5.520463 -0.127542 -3.289086
H 5.044334 -1.717894 -2.682252
H 4.408394 -1.124966 -4.228750
C 2.779935 -1.291932 3.392135
H 3.715216 -0.937680 3.840292
H 2.081118 -1.520876 4.204401
H 2.993870 -2.223175 2.857703
C 1.801080 1.028134 3.249902
H 2.656578 1.424834 3.807530
H 1.437672 1.810230 2.578880
H 1.013630 0.796137 3.973920
C -0.723445 1.996839 0.192225
C -2.088541 2.303511 0.277546
C 0.206129 3.048791 0.137174
C -0.195688 4.349593 -0.046038
C -2.464806 3.627247 0.108838

C -1.560274 4.664753 -0.054882
F -0.288665 0.832742 1.004888
H -2.841589 1.532777 0.402889
F 1.511395 2.719465 0.118643
F -3.777987 3.912865 0.128610
H 0.562584 5.114460 -0.174434
H -1.914923 5.680554 -0.173565

22

Final structure in terms of initial Cartesian coordinates:

Al -0.103138 -0.655176 -0.660159
N -1.436990 -0.948428 0.667494
N 1.337459 -1.281487 0.400596
C -1.323142 -1.929572 1.553517
C 1.138299 -2.284283 1.266550
C -0.122921 -2.641974 1.749604
H -0.162694 -3.475175 2.439095
C -2.650163 -0.185746 0.546968
C -3.740087 -0.706248 -0.169387
C -2.655718 1.124574 1.061434
C -4.862641 0.108080 -0.332667
C -3.799734 1.898679 0.869033
C -4.897693 1.396382 0.180429
H -5.715091 -0.271025 -0.890385
H -3.832108 2.912110 1.255652
H -5.777910 2.015920 0.036310
C 2.666597 -0.768562 0.213271
C 3.209202 0.094252 1.188468
C 3.369362 -1.069931 -0.966876
C 4.466783 0.650786 0.950813
C 4.619903 -0.478717 -1.163019
C 5.169078 0.374530 -0.216327
H 4.896398 1.326565 1.685880
H 5.168293 -0.692148 -2.076717
H 6.141021 0.827221 -0.386707
C -3.714586 -2.075597 -0.829441
H -2.815389 -2.607803 -0.502239
C -1.467994 1.668664 1.841342
H -0.562571 1.188816 1.452468
C 2.459373 0.486897 2.455038
H 1.522209 -0.079499 2.501911
C 2.800291 -1.988208 -2.034645
H 1.879210 -2.437271 -1.654254
C 2.333536 -3.046198 1.780559
H 2.019264 -3.928350 2.339070
H 2.971701 -3.351450 0.945061
H 2.945021 -2.417360 2.433983
C -2.513398 -2.327162 2.390237
H -2.196947 -2.885583 3.272002
H -3.092661 -1.453391 2.697577
H -3.177904 -2.964803 1.796551
C 2.099635 1.978223 2.420731
H 3.004346 2.595455 2.385235
H 1.493806 2.223200 1.543667
H 1.537854 2.257536 3.319226
C 3.267525 0.180563 3.722845
H 3.569995 -0.869370 3.778936
H 4.176898 0.789447 3.764674
H 2.673647 0.411628 4.612920
C 2.436619 -1.187147 -3.291609
H 1.776005 -0.346859 -3.055146
H 3.339106 -0.780683 -3.763514
H 1.923790 -1.827045 -4.015785
C 3.764534 -3.128989 -2.378326
H 4.690542 -2.751440 -2.825279
H 4.033984 -3.708130 -1.489034
H 3.300426 -3.807367 -3.101040
C -1.286683 3.177955 1.675550
H -1.248422 3.455200 0.616571
H -2.097258 3.739363 2.153117
H -0.352916 3.495641 2.149151
C -1.582807 1.289061 3.323466
H -2.512312 1.682400 3.750528
H -1.578159 0.202229 3.458905
H -0.743452 1.702313 3.893797
C -4.938964 -2.917613 -0.447769
H -5.856542 -2.494420 -0.870208
H -4.835715 -3.933254 -0.842561
H -5.075133 -2.979837 0.637097
C -3.622795 -1.929049 -2.354221
H -4.491441 -1.382081 -2.738580
H -2.717014 -1.393389 -2.642533
H -3.603639 -2.916773 -2.826964
C 0.055389 1.222283 -1.249491
C 1.166240 2.067806 -1.095566

C -1.053934 1.800717 -1.860783
C -1.131212 3.121094 -2.279938
C 1.110636 3.391786 -1.508822
C -0.020850 3.939312 -2.094923
F -0.396637 -1.745346 -1.901332
H 2.098396 1.714488 -0.660981
F -2.140469 1.020539 -2.073607
F 2.192113 4.173154 -1.339050
H -2.042442 3.487415 -2.740006
H -0.017816 4.979264 -2.402388

IM$_{1\text{-}20para}$

Final structure in terms of initial Cartesian coordinates:
Al -0.317043 0.144679 -0.809368
N 0.612188 -1.593503 -0.452112
N -2.029128 -0.780166 -0.343006
C 0.083646 -2.806732 -0.635417
C -2.286838 -2.082581 -0.390418
C -1.295132 -3.040040 -0.660942
H -1.620279 -4.064820 -0.784588
C 2.007529 -1.516970 -0.123859
C 2.371448 -1.604857 1.239384
C 2.976968 -1.328373 -1.124583
C 3.728586 -1.572879 1.563896
C 4.322703 -1.278059 -0.746643
C 4.702366 -1.419848 0.580313
H 4.032786 -1.654132 2.603019
H 5.083226 -1.134330 -1.509888
H 5.753116 -1.396500 0.854007
C -3.084518 0.130665 0.000916
C -4.041136 0.502242 -0.960310
C -3.080508 0.701543 1.290479
C -5.037807 1.406184 -0.585858
C -4.100298 1.594726 1.619439
C -5.079801 1.938192 0.695004
H -5.785228 1.707905 -1.314671
H -4.121625 2.042206 2.607810
H -5.863995 2.637597 0.968805
C 1.320082 -1.736094 2.334896
H 0.427639 -1.205425 1.985338
C 2.612529 -1.159626 -2.589938
H 1.540780 -1.361288 -2.698119
C -3.970585 0.014560 -2.399004
H -3.290941 -0.842973 -2.442054
C -1.989944 0.350352 2.293223
H -1.044846 0.303555 1.738371
C -3.689599 -2.584299 -0.139464
H -3.709438 -3.673766 -0.093197
H -4.076490 -2.177402 0.800112
H -4.367780 -2.252037 -0.931666
C 0.999628 -3.993325 -0.832675
H 0.428525 -4.919517 -0.911599
H 1.574223 -3.852543 -1.755384
H 1.724332 -4.086343 -0.019680
C -3.378285 1.115342 -3.290028
H -4.024674 2.000283 -3.281406
H -2.387351 1.418146 -2.936165
H -3.288331 0.766995 -4.324641
C -5.330875 -0.436257 -2.943066
H -5.813584 -1.167253 -2.285904
H -6.016032 0.410130 -3.057417
H -5.208002 -0.890683 -3.931164
C -1.787806 1.411329 3.375085
H -1.651278 2.403121 2.936026
H -2.630371 1.446941 4.074889
H -0.889776 1.169608 3.952557
C -2.225653 -1.025203 2.931123
H -3.212406 -1.061690 3.407519
H -2.164248 -1.834176 2.195904
H -1.470742 -1.220117 3.701979
C 2.868290 0.284753 -3.039236
H 2.291419 0.992632 -2.435367
H 3.931412 0.534593 -2.937452
H 2.587560 0.415219 -4.089886
C 3.370355 -2.141810 -3.491736
H 4.442983 -1.920320 -3.506790
H 3.250031 -3.178000 -3.159606
H 3.005137 -2.066207 -4.520815
C 0.917746 -3.196635 2.591548
H 1.804766 -3.810576 2.788392
H 0.266136 -3.254523 3.470568
H 0.370619 -3.631747 1.751783
C 1.761479 -1.100274 3.657005
H 2.518936 -1.710103 4.162654
H 2.164553 -0.096287 3.507917

H	0.903538	-1.030837	4.334239
C	1.825553	2.119405	0.980203
C	1.109276	3.250222	0.608904
C	3.098368	1.892053	0.494893
C	3.674713	2.825013	-0.366626
C	1.661421	4.186778	-0.244961
C	2.946183	3.950044	-0.718166
F	1.227426	1.228312	1.792954
H	4.668479	2.680288	-0.773912
H	3.620432	0.984696	0.782330
H	1.101233	5.063700	-0.544729
F	3.495059	4.850263	-1.546022
F	-0.133019	3.405156	1.071869

TS$_{1-20para}$

Final structure in terms of initial Cartesian coordinates:

Al	-0.086125	-0.005407	-0.809679
N	1.110377	-1.514255	-0.490167
N	-1.633248	-1.107076	-0.332067
C	0.780275	-2.793895	-0.650654
C	-1.681917	-2.431504	-0.461861
C	-0.550264	-3.224885	-0.714459
H	-0.718097	-4.283098	-0.864758
C	2.453210	-1.159793	-0.133581
C	2.801963	-1.164403	1.233838
C	3.360592	-0.727697	-1.116779
C	4.087965	-0.755183	1.589791
C	4.630714	-0.310954	-0.708417
C	4.998921	-0.327770	0.630297
H	4.376922	-0.761641	2.637518
H	5.340812	0.034847	-1.455404
H	5.991842	-0.004244	0.927486
C	-2.840739	-0.390794	-0.014339
C	-3.715164	-0.004667	-1.048157
C	-3.095440	-0.041545	1.324276
C	-4.869451	0.702488	-0.709115
C	-4.273913	0.649143	1.615235
C	-5.159117	1.017424	0.611942
H	-5.550767	1.015705	-1.495923
H	-4.494858	0.909852	2.646968
H	-6.066524	1.560814	0.857180
C	1.829368	-1.602730	2.318909
H	0.839243	-1.706334	1.864114
C	3.000283	-0.667142	-2.591075
H	2.003082	-1.103654	-2.716995
C	-3.422364	-0.283803	-2.514150
H	-2.570376	-0.970435	-2.573412
C	-2.138283	-0.381844	2.455388
H	-1.213006	-0.765715	2.016042
C	-3.012028	-3.136196	-0.349432
H	-2.881920	-4.218593	-0.364914
H	-3.531028	-2.845867	0.568102
H	-3.660875	-2.845745	-1.182754
C	1.871994	-3.826260	-0.795165
H	1.464378	-4.837502	-0.768123
H	2.377373	-3.676150	-1.756870
H	2.633445	-3.720466	-0.019467
C	-3.026256	1.016580	-3.227147
H	-3.859304	1.728751	-3.209716
H	-2.165930	1.485502	-2.739406
H	-2.767844	0.818247	-4.272873
C	-4.606794	-0.941173	-3.233107
H	-4.946901	-1.847150	-2.721104
H	-5.459892	-0.257743	-3.298610
H	-4.323858	-1.210799	-4.255529
C	-1.782726	0.869502	3.267114
H	-1.429265	1.669196	2.611411
H	-2.649361	1.240961	3.824840
H	-0.995921	0.641197	3.993002
C	-2.712415	-1.473100	3.366282
H	-3.654609	-1.145233	3.820147
H	-2.908582	-2.397835	2.814001
H	-2.010047	-1.704146	4.174904
C	2.939417	0.789383	-3.069110
H	2.227539	1.370175	-2.473104
H	3.923910	1.264771	-2.987434
H	2.627533	0.834092	-4.117853
C	3.980463	-1.477066	-3.448261
H	4.988439	-1.050274	-3.407385
H	4.046227	-2.517426	-3.113730
H	3.661424	-1.471395	-4.495365
C	2.223888	-2.966733	2.901076
H	3.227663	-2.924604	3.339138
H	1.523104	-3.260215	3.689881
H	2.220706	-3.752716	2.140107

C 1.721887 -0.565305 3.441816
H 2.657148 -0.489238 4.007390
H 1.475846 0.422363 3.043811
H 0.937098 -0.862644 4.146599
C 0.723177 1.969613 0.213441
C -0.229887 2.998685 0.190212
C 2.076670 2.312434 0.333079
C 2.459216 3.647673 0.226260
C 0.132881 4.319909 0.065277
C 1.491310 4.628856 0.090164
F 0.298895 0.757638 1.007660
H 3.504992 3.931138 0.264235
H 2.824973 1.533497 0.442550
H -0.619677 5.092063 -0.041814
F 1.859234 5.917720 -0.011490
F -1.524793 2.646178 0.142392

23

Final structure in terms of initial Cartesian coordinates:

Al 0.013715 -0.559525 -0.747076
N -1.496668 -1.253208 0.171489
N 1.293530 -1.188722 0.515837
C -1.387322 -2.410325 0.838641
C 1.088857 -2.304431 1.202938
C -0.165402 -2.946081 1.254170
H -0.202419 -3.892384 1.778185
C -2.783287 -0.622334 0.061038
C -3.461994 -0.636567 -1.171151
C -3.306402 0.068295 1.174275
C -4.659645 0.074600 -1.277955
C -4.512517 0.753296 1.020147
C -5.184876 0.766645 -0.196007
H -5.185706 0.083724 -2.228808
H -4.925291 1.297920 1.865670
H -6.117085 1.313961 -0.297510
C 2.556929 -0.503260 0.556363
C 2.636628 0.678338 1.316872
C 3.628069 -0.950438 -0.234242
C 3.836604 1.389630 1.298633
C 4.808201 -0.203329 -0.217593
C 4.917322 0.952751 0.541431
H 3.926534 2.303199 1.877611
H 5.648622 -0.526698 -0.826262
H 5.841627 1.522497 0.536466
C -2.923322 -1.377553 -2.382860
H -2.058583 -1.969533 -2.073297
C -2.583649 0.147035 2.512363
H -1.707933 -0.511136 2.481560
C 1.467773 1.146534 2.170203
H 0.544739 0.791959 1.697511
C 3.527561 -2.162695 -1.145958
H 2.582467 -2.676899 -0.942715
C 2.227685 -2.940742 1.959691
H 1.851226 -3.611682 2.732900
H 2.842794 -3.525295 1.265967
H 2.876376 -2.186837 2.411679
C -2.643931 -3.158569 1.205654
H -2.406050 -4.167745 1.543574
H -3.180230 -2.641306 2.006910
H -3.320419 -3.210126 0.347253
C 1.373226 2.670029 2.264343
H 2.208586 3.095388 2.831081
H 1.361112 3.125410 1.268184
H 0.453837 2.956386 2.784017
C 1.537118 0.512601 3.565584
H 1.486011 -0.580364 3.511530
H 2.472858 0.785511 4.066624
H 0.703695 0.857618 4.187570
C 3.493121 -1.724079 -2.616031
H 2.636267 -1.077650 -2.812142
H 4.408728 -1.180510 -2.875918
H 3.419169 -2.600460 -3.268553
C 4.677052 -3.150799 -0.911065
H 5.633564 -2.726244 -1.234155
H 4.777617 -3.422097 0.145084
H 4.513138 -4.065707 -1.489316
C -2.081047 1.575093 2.761462
H -1.411025 1.907602 1.963438
H -2.921081 2.277537 2.809692
H -1.540739 1.631833 3.713303
C -3.476006 -0.296202 3.679291
H -4.307277 0.401780 3.824691
H -3.904135 -1.290560 3.520827
H -2.897574 -0.317276 4.608352
C -3.959499 -2.344640 -2.966823

H -4.827580 -1.810628 -3.368017
H -3.514822 -2.915908 -3.787654
H -4.319501 -3.051369 -2.212072
C -2.446144 -0.384878 -3.450942
H -3.293884 0.182647 -3.853174
H -1.727999 0.332784 -3.041929
H -1.960955 -0.916099 -4.274939
C 0.009093 1.395792 -0.978580
C 1.161519 1.995755 -1.478376
C -1.030724 2.289519 -0.665346
C -0.909249 3.670518 -0.812444
C 1.349020 3.360312 -1.646434
C 0.286420 4.179321 -1.297757
F 0.258193 -1.443773 -2.151862
H -1.718949 4.348767 -0.568044
H -1.979163 1.902433 -0.299830
H 2.275480 3.763301 -2.036990
F 0.421921 5.504711 -1.447447
F 2.187056 1.195144 -1.843898

24

Final structure in terms of initial Cartesian coordinates:
C 0.000000 1.195127 -0.381910
C 0.000000 0.000000 -1.090183
C 0.000000 -1.195127 -0.381910
C 0.000000 -1.217785 1.004162
C 0.000000 0.000000 1.668109
C 0.000000 1.217785 1.004162
F 0.000000 2.335092 -1.070409
F 0.000000 0.000000 -2.421547
F 0.000000 -2.335092 -1.070409
H 0.000000 -2.156673 1.543094
H 0.000000 2.156673 1.543094
F 0.000000 0.000000 3.004502

IM$_{1-24}$

Final structure in terms of initial Cartesian coordinates:
Al -0.498458 0.360620 0.307138
N -2.370775 -0.267953 0.613873
N 0.118976 -1.402133 1.006084
C -2.796061 -1.273479 1.376370
C -0.551069 -2.280582 1.753036
C -1.924757 -2.181116 1.994611
H -2.369968 -2.937653 2.627314
C -3.352296 0.545287 -0.049577
C -3.578062 0.334806 -1.425840
C -4.019895 1.563690 0.654101
C -4.539663 1.120901 -2.061951
C -4.966042 2.330220 -0.029242
C -5.238511 2.103756 -1.370593
H -4.739180 0.974737 -3.118192
H -5.491056 3.122815 0.496935
H -5.981854 2.704905 -1.885330
C 1.449621 -1.751597 0.593650
C 2.566437 -1.320503 1.332551
C 1.603965 -2.495918 -0.592145
C 3.838612 -1.666041 0.873268
C 2.896339 -2.829218 -1.005786
C 4.009261 -2.420227 -0.280821
H 4.711920 -1.330983 1.425565
H 3.030682 -3.412833 -1.913760
H 5.008109 -2.680084 -0.618129
C -2.794619 -0.718258 -2.200780
H -1.756094 -0.680203 -1.842943
C -3.690812 1.892005 2.102135
H -3.123775 1.056269 2.525727
C 2.427264 -0.484510 2.593577
H 1.367253 -0.464453 2.870068
C 0.415226 -2.930816 -1.437226
H -0.502082 -2.585486 -0.947111
C 0.181881 -3.458040 2.350947
H 0.595392 -4.097685 1.565285
H 1.029490 -3.110670 2.951008
H -0.481341 -4.052663 2.980246
C -4.277493 -1.483465 1.590610
H -4.810942 -1.477807 0.635475
H -4.464206 -2.428539 2.101970
H -4.695888 -0.670329 2.193101
C 2.873492 0.960061 2.331208
H 3.921287 0.988318 2.007419
H 2.262077 1.421625 1.547725
H 2.782093 1.562145 3.241550

C 3.210215 -1.080928 3.769281
H 2.945441 -2.128704 3.945203
H 4.289941 -1.035349 3.591940
H 3.003420 -0.516455 4.683982
C 0.472874 -2.280183 -2.824132
H 0.513729 -1.188297 -2.751719
H 1.357585 -2.614304 -3.378829
H -0.410377 -2.558633 -3.410504
C 0.329976 -4.456304 -1.563029
H 1.210364 -4.860212 -2.074964
H 0.260366 -4.939976 -0.583734
H -0.553794 -4.740184 -2.144295
C -2.793965 3.136298 2.161716
H -1.870735 2.985018 1.592696
H -3.315363 4.001022 1.735699
H -2.530087 3.372174 3.198207
C -4.941083 2.096828 2.964862
H -5.479612 3.006673 2.679709
H -5.640407 1.258303 2.881344
H -4.657612 2.203496 4.016688
C -3.317243 -2.136898 -1.939709
H -4.375408 -2.211651 -2.216778
H -2.757307 -2.862479 -2.542065
H -3.212925 -2.427618 -0.890058
C -2.750532 -0.442769 -3.704452
H -3.728315 -0.599582 -4.173269
H -2.426747 0.581323 -3.912972
H -2.045857 -1.127211 -4.185393
C 2.106564 1.640543 -2.055726
C 3.131823 0.828653 -1.593593
C 1.992174 2.980640 -1.721318
C 2.957978 3.520522 -0.887601
C 4.084938 1.416224 -0.777393
C 4.010818 2.752472 -0.409358
F 1.174391 1.105339 -2.855266
F 4.924005 3.276683 0.409439
H 3.182872 -0.226038 -1.840848
F 5.105150 0.700939 -0.296323
F 2.892512 4.801161 -0.523758
H 1.162814 3.580383 -2.073189

$TS_{1\text{-}24}$

Final structure in terms of initial Cartesian coordinates:

Al -0.131172 -0.241777 0.848546
N -1.706751 -1.269501 0.330902
N 1.021993 -1.758887 0.450874
C -1.801310 -2.593568 0.431074
C 0.654393 -3.032599 0.564184
C -0.689425 -3.426817 0.636926
H -0.885094 -4.484096 0.757404
C -2.870591 -0.482273 0.027832
C -3.111165 -0.104790 -1.308926
C -3.702815 -0.036037 1.073282
C -4.230234 0.686888 -1.579347
C -4.798309 0.767559 0.751670
C -5.070756 1.120868 -0.562835
H -4.444574 0.970867 -2.605785
H -5.446198 1.124498 1.547845
H -5.931737 1.740066 -0.795143
C 2.371783 -1.410649 0.109980
C 3.275224 -1.017198 1.113737
C 2.728229 -1.361361 -1.254531
C 4.544712 -0.577791 0.728055
C 4.012825 -0.929585 -1.587486
C 4.916983 -0.534321 -0.608759
H 5.250727 -0.261334 1.491687
H 4.305965 -0.893590 -2.633444
H 5.907671 -0.189871 -0.888737
C -2.217866 -0.547950 -2.458777
H -1.294120 -0.951976 -2.034413
C -3.430162 -0.358218 2.534311
H -2.616587 -1.090905 2.582262
C 2.914235 -1.025792 2.589638
H 1.913455 -1.459840 2.694899
C 1.769646 -1.769282 -2.363434
H 0.782464 -1.928919 -1.917734
C 1.717380 -4.099816 0.645022
H 2.464120 -3.982619 -0.143215
H 2.248290 -4.000700 1.599508
H 1.281706 -5.097845 0.587066
C -3.160280 -3.243139 0.345591
H -3.704459 -2.903578 -0.539527
H -3.074266 -4.329850 0.322721

H -3.761421 -2.954138 1.214887
C 2.868050 0.404875 3.141328
H 3.856683 0.874498 3.083457
H 2.161572 1.022478 2.576034
H 2.555801 0.400267 4.190824
C 3.887131 -1.888376 3.402795
H 3.941545 -2.910543 3.014567
H 4.899262 -1.470316 3.381142
H 3.569639 -1.933120 4.449420
C 1.630805 -0.676718 -3.429607
H 1.318207 0.272072 -2.986882
H 2.574993 -0.516351 -3.961119
H 0.884199 -0.978175 -4.172950
C 2.211748 -3.083304 -3.022383
H 3.203263 -2.972455 -3.475635
H 2.261226 -3.908018 -2.305433
H 1.509650 -3.366679 -3.813603
C -2.970054 0.903837 3.277188
H -2.078880 1.334900 2.809234
H -3.761746 1.661862 3.269897
H -2.732085 0.669983 4.320220
C -4.653300 -0.965718 3.232314
H -5.468075 -0.237896 3.307536
H -5.038665 -1.840332 2.698491
H -4.391881 -1.271953 4.250062
C -2.886494 -1.660686 -3.275881
H -3.834050 -1.312177 -3.702438
H -2.236888 -1.971046 -4.101703
H -3.096536 -2.542804 -2.662950
C -1.837801 0.625723 -3.368297
H -2.711432 1.031617 -3.888951
H -1.370107 1.434315 -2.799185
H -1.129039 0.293161 -4.132662
C 0.453924 1.812459 -0.170328
C 1.815663 2.162980 -0.186808
C -0.546798 2.798169 -0.263945
C -0.163313 4.120168 -0.162287
C 2.138454 3.502576 -0.081240
C 1.171912 4.501293 -0.064030
F 0.150748 0.569033 -0.961485
F 1.512891 5.788638 0.051741
H 2.606699 1.419588 -0.196094
F 3.420232 3.872105 -0.006740
F -1.088861 5.083412 -0.162937
H -1.600405 2.545644 -0.316463

25

Final structure in terms of initial Cartesian coordinates:

Al 0.02919 -0.651371 -0.774898
N -1.485561 -1.433609 0.052745
N 1.305754 -1.455710 0.380332
C -1.392795 -2.655335 0.592240
C 1.091372 -2.648670 0.922042
C -0.176196 -3.261462 0.925718
H -0.228483 -4.261440 1.335987
C -2.760677 -0.769875 0.008519
C -3.444939 -0.659462 -1.215299
C -3.259327 -0.168914 1.182355
C -4.627082 0.084561 -1.248448
C -4.447491 0.557732 1.099031
C -5.127269 0.692767 -0.105769
H -5.158899 0.190045 -2.190528
H -4.838790 1.039156 1.991454
H -6.043972 1.272465 -0.151456
C 2.578551 -0.796997 0.487795
C 2.704419 0.239187 1.435160
C 3.609164 -1.102657 -0.416383
C 3.911484 0.934621 1.488719
C 4.796023 -0.368104 -0.329151
C 4.952289 0.635152 0.616069
H 4.037085 1.736758 2.208316
H 5.603811 -0.581151 -1.024331
H 5.879929 1.197000 0.665286
C -2.926573 -1.296261 -2.493277
H -2.061662 -1.917033 -2.246787
C -2.525947 -0.231055 2.514466
H -1.677446 -0.918223 2.420729
C 1.555851 0.592247 2.368201
H 0.621800 0.349823 1.847386
C 3.459745 -2.147873 -1.510419
H 2.513618 -2.677755 -1.360445
C 2.239249 -3.397743 1.549980
H 2.866948 -3.827668 0.760927
H 2.873338 -2.730033 2.138455
H 1.876460 -4.207879 2.183525

C -2.662060 -3.408803 0.898552
H -3.330766 -3.392792 0.032234
H -2.442870 -4.441844 1.169871
H -3.199276 -2.935738 1.726296
C 1.510876 2.081924 2.712272
H 2.365290 2.385067 3.326629
H 1.501510 2.693648 1.803837
H 0.607041 2.306019 3.285946
C 1.601186 -0.262641 3.641274
H 1.504794 -1.329336 3.412906
H 2.546963 -0.110807 4.173759
H 0.781974 0.011079 4.315585
C 3.388064 -1.473034 -2.886497
H 2.537353 -0.789976 -2.946154
H 4.307579 -0.912633 -3.090143
H 3.266286 -2.226622 -3.671154
C 4.600720 -3.172401 -1.479231
H 5.555869 -2.707716 -1.746731
H 4.720953 -3.624508 -0.489367
H 4.410619 -3.970836 -2.203327
C -1.966789 1.151481 2.872664
H -1.300926 1.526509 2.089317
H -2.778712 1.877013 2.996701
H -1.407196 1.106649 3.814120
C -3.425592 -0.740021 3.647990
H -4.237135 -0.034517 3.854473
H -3.880022 -1.707500 3.412887
H -2.843804 -0.851154 4.568664
C -3.977839 -2.203002 -3.142893
H -4.852167 -1.631893 -3.473238
H -3.553383 -2.697421 -4.022275
H -4.325232 -2.975545 -2.449146
C -2.456577 -0.217109 -3.476646
H -3.305366 0.387181 -3.817693
H -1.729570 0.458747 -3.014961
H -1.983578 -0.676052 -4.349739
C 0.133112 1.315262 -0.790983
C 1.365139 1.924650 -1.094709
C -0.956599 2.161285 -0.520751
C -0.808886 3.540371 -0.535128
C 1.494361 3.304442 -1.113369
C 0.412963 4.128475 -0.829281
F 0.258601 -1.350580 -2.284712
F 0.543704 5.455192 -0.848704
H 2.258990 1.346706 -1.321602
F 2.664254 3.883288 -1.404934
F -1.844671 4.344905 -0.270322
H -1.948420 1.775582 -0.294899

$IM_{1\text{-}24meta}$

Final structure in terms of initial Cartesian coordinates:
Al -0.318038 0.058576 -0.735528
N 0.695257 -1.628193 -0.407371
N -1.981481 -0.958573 -0.302963
C 0.239087 -2.874168 -0.555564
C -2.167117 -2.275168 -0.326137
C -1.124451 -3.183908 -0.569659
H -1.393150 -4.227709 -0.667783
C 2.077516 -1.449145 -0.065749
C 2.423645 -1.472740 1.304425
C 3.041478 -1.198456 -1.057390
C 3.767543 -1.317032 1.647806
C 4.366544 -0.997436 -0.658417
C 4.735865 -1.078789 0.676302
H 4.061276 -1.353080 2.692680
H 5.120736 -0.783999 -1.411235
H 5.773736 -0.940877 0.964270
C -3.095441 -0.102939 -0.001464
C -4.053051 0.180674 -0.991363
C -3.152995 0.503099 1.271000
C -5.110335 1.032218 -0.663778
C -4.231098 1.343340 1.551719
C -5.210124 1.598805 0.598799
H -5.860197 1.264627 -1.415162
H -4.302141 1.818832 2.524375
H -6.040168 2.257145 0.836465
C 1.365211 -1.645668 2.387890
H 0.438738 -1.201967 2.004171
C 2.696322 -1.148615 -2.536726
H 1.650296 -1.455710 -2.650977
C -3.922759 -0.340752 -2.413904
H -3.197800 -1.161236 -2.419290
C -2.069294 0.244349 2.309257
H -1.107547 0.241975 1.779437
C -3.543288 -2.847957 -0.078319

H -3.977545 -2.418441 0.829634
H -4.220014 -2.600004 -0.902213
H -3.497577 -3.932934 0.023390
C 1.223179 -4.009604 -0.721098
H 1.951423 -4.034851 0.093579
H 0.708202 -4.970057 -0.772099
H 1.787537 -3.862442 -1.649120
C -3.368715 0.767393 -3.320338
H -4.060757 1.616620 -3.349043
H -2.404418 1.132890 -2.952080
H -3.235556 0.398687 -4.343114
C -5.243591 -0.879023 -2.975339
H -5.701148 -1.617507 -2.308599
H -5.971006 -0.075337 -3.128738
H -5.073204 -1.352447 -3.947338
C -1.959294 1.342288 3.367003
H -1.850481 2.327106 2.904006
H -2.829777 1.356457 4.032203
H -1.074886 1.159987 3.985363
C -2.237380 -1.128131 2.974374
H -3.229598 -1.211737 3.432891
H -2.115670 -1.947796 2.258732
H -1.488074 -1.262638 3.763294
C 2.829607 0.277028 -3.087012
H 2.134734 0.956388 -2.582743
H 3.846284 0.657895 -2.939676
H 2.608389 0.292499 -4.159764
C 3.563948 -2.116865 -3.351521
H 4.613219 -1.802803 -3.352000
H 3.523308 -3.135403 -2.952229
H 3.226756 -2.140859 -4.392628
C 1.071592 -3.122074 2.695329
H 1.999226 -3.655319 2.934880
H 0.404860 -3.197931 3.561512
H 0.581548 -3.630349 1.861427
C 1.729022 -0.923278 3.688770
H 2.531354 -1.441936 4.225374
H 2.040345 0.107670 3.506218
H 0.858671 -0.904126 4.353324
C 1.525495 2.376761 1.156937
C 0.766397 3.331004 0.498582
C 2.769034 1.978250 0.696363
C 3.230593 2.567459 -0.469998
C 1.268255 3.888572 -0.668635
C 2.507719 3.522609 -1.169605
F 0.995419 1.802805 2.245209
F 0.522778 4.784394 -1.313592
F 4.424925 2.193851 -0.949387
H 3.336412 1.204610 1.201126
F -0.450788 3.647996 0.939141
H 2.885991 3.957967 -2.085911

$TS_{1\text{-}24meta}$

Final structure in terms of initial Cartesian coordinates:

Al 0.114557 -0.092120 0.824456
N -1.100165 -1.580109 0.497883
N 1.643871 -1.202127 0.332555
C -0.784404 -2.865306 0.649125
C 1.679809 -2.528877 0.448685
C 0.541288 -3.312112 0.699960
H 0.698349 -4.373122 0.842077
C -2.441536 -1.208425 0.149242
C -2.798898 -1.210666 -1.216188
C -3.336547 -0.764373 1.138130
C -4.082250 -0.785980 -1.563427
C -4.603597 -0.331094 0.737764
C -4.980756 -0.344871 -0.598336
H -4.378739 -0.789052 -2.608944
H -5.303724 0.026974 1.488107
H -5.970068 -0.005757 -0.889522
C 2.855971 -0.493628 0.011307
C 3.735268 -0.114674 1.043081
C 3.107960 -0.144980 -1.327950
C 4.889946 0.590862 0.702007
C 4.286633 0.544242 -1.620792
C 5.174888 0.909583 -0.619054
H 5.574763 0.900462 1.487179
H 4.505059 0.807282 -2.652408
H 6.081490 1.453528 -0.865716
C -1.839155 -1.661701 -2.307958
H -0.842879 -1.757298 -1.864697
C -2.966547 -0.708404 2.610344
H -1.974806 -1.158903 2.731066
C 3.448992 -0.401175 2.508916
H 2.592016 -1.081713 2.568767

C 2.149040 -0.485875 -2.457465
H 1.221102 -0.861409 -2.016467
C 3.002953 -3.244065 0.325725
H 3.522350 -2.949696 -0.590179
H 3.655825 -2.965932 1.160187
H 2.863577 -4.325369 0.331973
C -1.887323 -3.884249 0.802618
H -2.662433 -3.760542 0.043617
H -1.493931 -4.900475 0.757779
H -2.371207 -3.736991 1.775829
C 3.069481 0.896965 3.234929
H 3.909635 1.600548 3.220268
H 2.212957 1.381036 2.755196
H 2.813440 0.692297 4.279984
C 4.632483 -1.075171 3.214136
H 4.957603 -1.982090 2.694167
H 5.493702 -0.401620 3.274645
H 4.355993 -1.346968 4.237739
C 1.802372 0.760686 -3.280466
H 1.471341 1.575521 -2.632043
H 2.667321 1.110516 -3.854451
H 1.002572 0.535185 -3.992987
C 2.716046 -1.587385 -3.360734
H 3.662240 -1.269693 -3.813427
H 2.902511 -2.511296 -2.803816
H 2.013919 -1.816183 -4.170170
C -2.883975 0.747004 3.088702
H -2.165942 1.318171 2.490509
H -3.861475 1.236482 3.009190
H -2.568465 0.787170 4.136552
C -3.951933 -1.506390 3.472716
H -4.954501 -1.066694 3.437829
H -4.032733 -2.545428 3.137326
H -3.626850 -1.505841 4.517955
C -2.236639 -3.034747 -2.866680
H -3.250851 -3.004407 -3.280873
H -1.552473 -3.330542 -3.669091
H -2.208334 -3.813150 -2.098725
C -1.749120 -0.641424 -3.448224
H -2.686994 -0.586567 -4.011773
H -1.514343 0.356308 -3.069248
H -0.963609 -0.941051 -4.151094
C -0.667811 1.873704 -0.278363
C 0.314366 2.875164 -0.248173
C -2.023905 2.234401 -0.341186
C -2.341690 3.569947 -0.168207
C -0.058463 4.187183 -0.049962
C -1.395021 4.575637 -0.024153
F -0.300721 0.686505 -1.062085
F 0.901645 5.104685 0.104314
F -3.637898 3.915402 -0.162517
H -2.807675 1.492558 -0.448731
F 1.606618 2.515119 -0.200323
H -1.675260 5.612706 0.100513

26

Final structure in terms of initial Cartesian coordinates:
Al 0.035145 -0.601866 -0.744408
N -1.490376 -1.344364 0.102296
N 1.291460 -1.277149 0.512438
C -1.386699 -2.529573 0.719972
C 1.079798 -2.423219 1.146160
C -0.170269 -3.074498 1.139877
H -0.212108 -4.043534 1.620276
C -2.779256 -0.717474 -0.013409
C -3.422515 -0.676744 -1.263704
C -3.338818 -0.084633 1.116638
C -4.619429 0.035371 -1.371935
C -4.541981 0.604620 0.959021
C -5.177839 0.675176 -0.274634
H -5.117600 0.087919 -2.336398
H -4.981517 1.107970 1.816334
H -6.107281 1.226683 -0.377689
C 2.545974 -0.580695 0.611443
C 2.593119 0.567124 1.425557
C 3.637906 -0.976771 -0.177681
C 3.780936 1.297456 1.458959
C 4.805321 -0.213107 -0.106578
C 4.881508 0.911029 0.702397
H 3.846066 2.187191 2.076935
H 5.661418 -0.495911 -0.713419
H 5.795000 1.496816 0.735536
C -2.849957 -1.363318 -2.491825
H -1.985270 -1.957835 -2.187488
C -2.661342 -0.073101 2.480533

H -1.783243 -0.728093 2.446869
C 1.399851 0.978898 2.274819
H 0.490627 0.646042 1.760638
C 3.574131 -2.149352 -1.142979
H 2.626657 -2.676420 -0.990999
C 2.205895 -3.084174 1.900315
H 2.829970 -3.649358 1.198508
H 2.848380 -2.345853 2.385538
H 1.816271 -3.777786 2.646569
C -2.645002 -3.302657 1.023290
H -3.299500 -3.323684 0.146731
H -2.406161 -4.323294 1.323991
H -3.205140 -2.823976 1.832117
C 1.290436 2.493515 2.453937
H 2.106522 2.890393 3.067429
H 1.303716 3.006186 1.485916
H 0.354573 2.743959 2.962534
C 1.437852 0.267326 3.633364
H 1.397479 -0.821000 3.516219
H 2.357812 0.518069 4.173659
H 0.585296 0.570632 4.251063
C 3.584729 -1.650739 -2.594244
H 2.733961 -0.996696 -2.792291
H 4.506819 -1.096710 -2.803567
H 3.533080 -2.499972 -3.283699
C 4.722646 -3.139604 -0.911962
H 5.686359 -2.693583 -1.180238
H 4.787719 -3.458015 0.133665
H 4.587600 -4.028270 -1.536812
C -2.172939 1.340747 2.822151
H -1.470604 1.713496 2.071408
H -3.015046 2.040452 2.868983
H -1.673735 1.349419 3.797897
C -3.592796 -0.577234 3.591088
H -4.429768 0.112204 3.743802
H -4.013789 -1.562017 3.366589
H -3.047019 -0.646919 4.537397
C -3.863872 -2.318648 -3.131687
H -4.730675 -1.778705 -3.527611
H -3.398130 -2.853694 -3.965157
H -4.229565 -3.057135 -2.410878
C -2.363414 -0.326234 -3.512016
H -3.209647 0.244373 -3.912701
H -1.664810 0.385650 -3.061053
H -1.852285 -0.820184 -4.343444
C 0.019793 1.371640 -0.875453
C 1.181071 1.990022 -1.316085
C -1.048048 2.220776 -0.532848
C -0.904619 3.597255 -0.614355
C 1.318885 3.372049 -1.398372
C 0.270081 4.202291 -1.042578
F 0.318214 -1.387952 -2.197403
F 2.469924 3.892977 -1.832002
F -1.935609 4.386420 -0.271908
H -2.007890 1.830338 -0.206221
F 2.237463 1.243553 -1.696451
H 0.364222 5.279586 -1.105947

IM$_{1\text{-}24para}$

Final structure in terms of initial Cartesian coordinates:
Al 0.396669 -0.304192 0.471389
N -0.573269 -1.615803 -0.672697
N 2.084401 -0.859104 -0.437951
C -0.081635 -2.582156 -1.449462
C 2.297845 -1.859417 -1.290189
C 1.283141 -2.726047 -1.716936
H 1.572897 -3.537282 -2.371859
C -1.999034 -1.436966 -0.636783
C -2.602665 -0.663592 -1.650338
C -2.755017 -1.974403 0.418194
C -3.974100 -0.424461 -1.573252
C -4.128444 -1.714834 0.448345
C -4.736766 -0.944117 -0.532919
H -4.455031 0.181767 -2.334945
H -4.727321 -2.124851 1.258220
H -5.804554 -0.749683 -0.491657
C 3.191004 -0.009247 -0.089923
C 4.072816 -0.403876 0.934201
C 3.327576 1.236380 -0.729841
C 5.104708 0.463596 1.291923
C 4.381916 2.067667 -0.342470
C 5.266732 1.689068 0.657022
H 5.789417 0.178342 2.086009
H 4.503122 3.032674 -0.828192
H 6.077123 2.350407 0.948089

C -1.783551 -0.084785 -2.796202
H -0.867839 -0.677933 -2.894582
C -2.132456 -2.821195 1.515957
H -1.087109 -3.009196 1.247340
C 3.894557 -1.712036 1.688780
H 3.207061 -2.346743 1.120161
C 2.366532 1.709948 -1.809777
H 1.608672 0.932096 -1.960003
C 3.686818 -2.091713 -1.836446
H 4.078762 -1.173256 -2.283655
H 4.376298 -2.368717 -1.031644
H 3.683247 -2.884380 -2.585603
C -1.028468 -3.550072 -2.118974
H -1.627510 -3.034567 -2.878311
H -0.480113 -4.361568 -2.599081
H -1.731306 -3.968124 -1.391946
C 3.254057 -1.445574 3.057416
H 3.902824 -0.802002 3.662290
H 2.287635 -0.941311 2.949435
H 3.099023 -2.383974 3.600804
C 5.210090 -2.481299 1.853059
H 5.718370 -2.626086 0.893940
H 5.901563 -1.956372 2.520465
H 5.018258 -3.465506 2.291821
C 1.646781 2.992662 -1.376956
H 1.129168 2.854481 -0.422644
H 2.357970 3.817256 -1.253561
H 0.908079 3.295143 -2.126531
C 3.081997 1.916475 -3.150097
H 3.841438 2.702522 -3.074617
H 3.578914 1.000711 -3.486148
H 2.364105 2.218482 -3.920285
C -2.149017 -2.070434 2.852467
H -1.587020 -1.134126 2.784221
H -3.174988 -1.823825 3.149486
H -1.704370 -2.683858 3.643810
C -2.829893 -4.179978 1.651551
H -3.864769 -4.063703 1.990996
H -2.848890 -4.722509 0.700800
H -2.309094 -4.799005 2.389152
C -2.515145 -0.152570 -4.140250
H -3.369040 0.532053 -4.171351
H -1.837049 0.138629 -4.948484
H -2.884727 -1.162340 -4.347945
C -1.361318 1.356896 -2.489156
H -2.236261 2.006709 -2.392229
H -0.800353 1.416101 -1.547909
H -0.723223 1.749916 -3.289727
C -1.773090 3.121857 0.522137
C -0.973183 3.141766 1.656248
C -2.911451 2.325596 0.521785
C -3.254155 1.546621 1.615463
C -1.290123 2.398683 2.780571
C -2.429233 1.611240 2.728786
F -1.452365 3.850492 -0.549822
F 0.124196 3.899083 1.637208
H -0.648311 2.409364 3.651836
H -4.133623 0.915666 1.592323
F -3.698323 2.353120 -0.557454
F -2.749403 0.880564 3.805022

$TS_{1\text{-}24para}$

Final structure in terms of initial Cartesian coordinates:
Al 0.000013 -0.113131 0.800890
N -1.392569 -1.400217 0.322369
N 1.392573 -1.400215 0.322313
C -1.247583 -2.724057 0.379206
C 1.247583 -2.724055 0.379148
C 0.000004 -3.353414 0.484700
H 0.000006 -4.432541 0.560361
C -2.698744 -0.860209 0.051719
C -3.059695 -0.612259 -1.286068
C -3.564960 -0.539904 1.112272
C -4.324035 -0.081020 -1.542745
C -4.810209 0.011904 0.806057
C -5.196689 0.231587 -0.508791
H -4.623804 0.104198 -2.570794
H -5.487408 0.272403 1.615491
H -6.172887 0.654200 -0.726793
C 2.698748 -0.860197 0.051683
C 3.564917 -0.539828 1.112254
C 3.059722 -0.612227 -1.286096
C 4.810134 0.012074 0.806075
C 4.324033 -0.080900 -1.542737
C 5.196636 0.231781 -0.508762

H 5.487286 0.272620 1.615533
H 4.623821 0.104323 -2.570780
H 6.172807 0.654469 -0.726739
C -2.121587 -0.896931 -2.448511
H -1.132274 -1.117498 -2.036907
C -3.183188 -0.743261 2.569405
H -2.232041 -1.286971 2.603487
C 3.183133 -0.743220 2.569376
H 2.231950 -1.286867 2.603447
C 2.121694 -0.897000 -2.448581
H 1.132385 -1.117675 -2.037021
C 2.475272 -3.602215 0.353750
H 3.101895 -3.384220 -0.514566
H 3.086886 -3.397895 1.239433
H 2.201107 -4.657547 0.347116
C -2.475267 -3.602227 0.353882
H -3.101940 -3.384257 -0.514405
H -2.201080 -4.657554 0.347247
H -3.086834 -3.397920 1.239602
C 2.975004 0.609969 3.262692
H 3.907989 1.185173 3.271146
H 2.212534 1.202420 2.747514
H 2.655798 0.463841 4.300214
C 4.225178 -1.573455 3.329091
H 4.415932 -2.535466 2.842663
H 5.180573 -1.042556 3.398292
H 3.881364 -1.767761 4.350110
C 1.982847 0.322353 -3.367107
H 1.703487 1.212026 -2.797905
H 2.918810 0.533042 -3.895749
H 1.213622 0.137969 -4.124117
C 2.577474 -2.119840 -3.254418
H 3.583901 -1.964629 -3.659375
H 2.595828 -3.026395 -2.641917
H 1.898169 -2.296661 -4.095303
C -2.974977 0.609943 3.262662
H -2.212246 1.202197 2.747645
H -3.907848 1.185334 3.270796
H -2.656098 0.463838 4.300289
C -4.225284 -1.573403 3.329154
H -5.180627 -1.042419 3.398400
H -4.416148 -2.535393 2.842726
H -3.881449 -1.767747 4.350158
C -2.577182 -2.119830 -3.254359
H -3.583640 -1.964779 -3.659302
H -1.897857 -2.296528 -4.095254
H -2.595374 -3.026393 -2.641862
C -1.982827 0.322459 -3.367002
H -2.918784 0.533062 -3.895688
H -1.703595 1.212141 -2.797746
H -1.213540 0.138184 -4.123975
C -0.000022 2.094461 -0.176214
C 1.190208 2.832778 -0.159093
C -1.190244 2.832799 -0.159527
C -1.213976 4.196465 0.059908
C 1.213878 4.196442 0.060360
C -0.000061 4.863400 0.157658
F 0.000067 0.886107 -1.016894
F 2.342498 2.151704 -0.228532
H 2.156840 4.723233 0.140070
H -2.156957 4.723276 0.139264
F -2.342521 2.151742 -0.229402
F -0.000083 6.191522 0.346451

27

Final structure in terms of initial Cartesian coordinates:

Al 0.013282 -0.491178 -0.771997
N -1.533211 -1.241938 0.038852
N 1.264780 -1.294693 0.422804
C -1.448971 -2.473625 0.565880
C 1.017396 -2.466453 0.995206
C -0.248823 -3.078398 0.948171
H -0.319650 -4.073331 1.367994
C -2.830070 -0.616993 -0.004175
C -3.511764 -0.504752 -1.226629
C -3.365894 -0.085534 1.185002
C -4.734576 0.170256 -1.243352
C -4.593666 0.571407 1.119634
C -5.275599 0.706324 -0.083763
H -5.268265 0.276141 -2.184173
H -5.016440 0.999321 2.024870
H -6.226399 1.229699 -0.116013
C 2.551750 -0.667066 0.550826
C 2.658472 0.440729 1.411705
C 3.625926 -1.092797 -0.248101

C	3.881284	1.109554	1.472271
C	4.829340	-0.391236	-0.149886
C	4.960573	0.698720	0.698955
H	3.990436	1.967454	2.128387
H	5.672384	-0.695588	-0.764458
H	5.903606	1.234120	0.755419
C	-2.940844	-1.061128	-2.519324
H	-2.088071	-1.700123	-2.275732
C	-2.628438	-0.141247	2.516030
H	-1.766915	-0.811211	2.418231
C	1.492217	0.874898	2.285649
H	0.564829	0.584974	1.779148
C	3.508202	-2.232157	-1.248665
H	2.556314	-2.746681	-1.083273
C	2.117139	-3.200279	1.721608
H	2.739390	-3.740291	0.998884
H	2.769907	-2.508978	2.259467
H	1.697878	-3.924838	2.420870
C	-2.721301	-3.245084	0.816505
H	-3.369844	-3.204935	-0.063893
H	-2.497520	-4.284700	1.057627
H	-3.283164	-2.806171	1.646649
C	1.440890	2.388327	2.498862
H	2.262465	2.736391	3.134382
H	1.493444	2.921570	1.543492
H	0.506849	2.664906	2.997869
C	1.524171	0.130040	3.626287
H	1.432740	-0.952090	3.482626
H	2.465017	0.325930	4.153177
H	0.698205	0.455459	4.268153
C	3.479528	-1.692713	-2.685045
H	2.597239	-1.072968	-2.852310
H	4.375276	-1.095559	-2.890417
H	3.453229	-2.525188	-3.396356
C	4.641794	-3.252538	-1.083587
H	5.603907	-2.827150	-1.388301
H	4.744241	-3.590317	-0.046940
H	4.456541	-4.126295	-1.716376
C	-2.092591	1.249715	2.879502
H	-1.416068	1.625070	2.108279
H	-2.916935	1.965701	2.973800
H	-1.557070	1.219419	3.835401
C	-3.511407	-0.673677	3.651566
H	-4.328035	0.019690	3.878162
H	-3.957900	-1.642703	3.407079
H	-2.918967	-0.791965	4.564563
C	-3.956109	-1.920816	-3.279507
H	-4.807288	-1.327558	-3.630517
H	-3.482444	-2.369343	-4.158375
H	-4.346336	-2.728117	-2.651150
C	-2.425431	0.079773	-3.405952
H	-3.257759	0.713983	-3.733244
H	-1.713353	0.716731	-2.870569
H	-1.920302	-0.319992	-4.290300
C	0.225279	1.480030	-0.859156
C	1.439538	1.998719	-1.308323
C	-0.695508	2.469292	-0.526934
C	-0.465713	3.838230	-0.577366
C	1.767834	3.344640	-1.389727
C	0.785091	4.246066	-1.012384
F	0.235262	-1.206012	-2.273032
F	2.383402	1.128415	-1.713851
H	-1.233370	4.550828	-0.303795
H	2.736911	3.672012	-1.743781
F	-1.925122	2.091392	-0.127664
F	1.048604	5.555879	-1.084443
